# Synthesis of substituted 8*H*-benzo[*h*]pyrano[2,3-*f*]quinazolin-8-ones via photochemical 6π-electrocyclization of pyrimidines containing an allomaltol fragment

**DOI:** 10.3762/bjoc.19.58

**Published:** 2023-06-07

**Authors:** Constantine V Milyutin, Andrey Nikolaevich Komogortsev, Boris V Lichitsky, Mikhail E Minyaev, Valeriya G Melekhina

**Affiliations:** 1 N.D. Zelinsky Institute of Organic Chemistry, Russian Academy of Sciences, Leninsky Pr., 47, Moscow, 119991, Russian Federation

**Keywords:** allomaltol, dihydrobenzo[*h*]pyrano[2,3-*f*]quinazolines, 6π-electrocyclization, photocyclization, pyrimidines

## Abstract

For the first time, we elaborated a method for the synthesis of pyrimidines containing an allomaltol unit. The suggested approach is based on the reaction of 2-(1-(dimethylamino)-3-oxo-3-arylprop-1-en-2-yl)-3-hydroxy-6-methyl-4*H*-pyran-4-ones with cyanamide. The photochemical behavior of the obtained pyrimidines was investigated. It was shown that for the hydroxy derivatives the main pathway of phototransformation is a 6π-electrocyclization of the 1,3,5-hexatriene system and subsequent [1,9]-*H* sigmatropic shift leading to dihydrobenzo[*h*]pyrano[2,3-*f*]quinazolines. At the same time, for methylated analogues the photoreaction proceeds in two directions resulting in the formation of a mixture of the corresponding dihydrobenzo[*h*]pyrano[2,3-*f*]quinazolines and polyaromatic products. The obtained dihydro derivatives are stable compounds and do not undergo aromatization upon further UV irradiation. The structures of two of the dihydrobenzo[*h*]pyrano[2,3-*f*]quinazolines were confirmed by X-ray diffraction analysis. Based on the performed studies, a two-stage telescopic method for the synthesis of polyaromatic benzo[*h*]pyrano[2,3-*f*]quinazolines including the initial photocyclization of the starting pyrimidines and the final dehydration was proposed.

## Introduction

Photochemical processes involve absorption of UV light leading to the generation of molecules in the excited state and subsequent chemical transformations [1–2]. After excitation the electron distribution significantly changes leading to substantial alterations in the chemical behavior of the starting compound. Thereby, UV-induced reactions are a useful addition to common thermal processes. In this regard, photochemical transformations have found wide applications in various areas of science and technology [3–5]. For example, the use of photoreactions for the preparation of complex natural products allows reducing the number of synthetic steps [6–9]. Moreover, in some cases photochemical processes can open access to complicated multifunctional systems which are unavailable by other methods. Wherein, usually UV-promoted reactions are environmentally friendly due to the absence of toxic reagents, which is especially perspective in context of green chemistry [9–10]. Thus, studies of the photochemical behavior of organic compounds play an important role in modern chemistry allowing to create novel original synthetic methods.

**Scheme 1:**
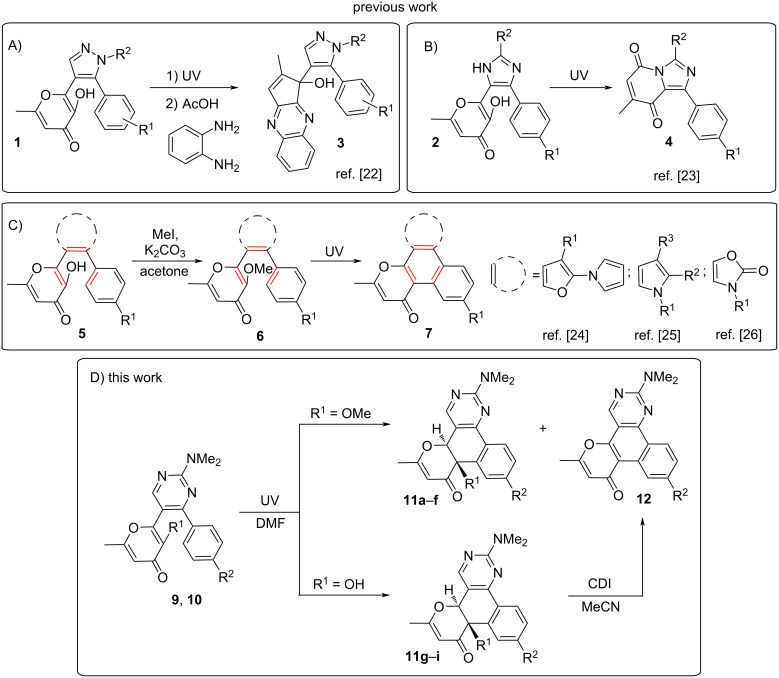
Photochemical behavior of terarylenes containing an allomaltol fragment.

UV-initiated reactions of heterocyclic systems are a significant part of organic photochemistry and there are numerous such transformations for various classes of heterocycles known in the literature [11–15]. Among them, photoreactions of compounds containing the 3-hydroxypyran-4-one (allomaltol) fragment attract considerable attention due to the unique photochemical properties of compounds of this class. Previously it was shown that allomaltol derivatives under UV irradiation undergo contraction of the pyranone ring leading to the formation of α-hydroxy-1,2-diketones. This phototransformation is initiated by an excited state intramolecular proton transfer (ESIPT) process. In most cases the resulting α-hydroxy-1,2-diketones are unstable and their further reactions open access to various original products [16–20]. The photochemical behavior of terarylenes containing an allomaltol fragment deserves special attention. In this case, two types of photoprocesses are possible: a 6π-electrocyclization of the 1,3,5-hexatriene system and the aforementioned ESIPT-induced transformation to α-hydroxy-1,2-diketone [21–25]. However, the direction of the transformation in each case depends on the structure of the bridge fragment. Thus, for example, the 1,3,5-hexatriene cyclization does not occur for pyrazole **1** and imidazole **2** derivatives, and the obtained products are formed exclusively as a result of ESIPT-induced reactions (Scheme 1A and B). At the same time, both directions of phototransformation are realized for terarylenes with furan, pyrrole, and oxazolone-bridge fragments (Scheme 1C). However, the suppression of the ESIPT process in such systems makes it possible to perform the 6π-electrocyclization leading to polyaromatic products. Therefore, it is complicate to predict the photochemical behavior of terarylenes containing a 3-hydroxypyran-4-one unit.

It should be noted that all the products considered above were constructed on the basis of five-membered heterocyclic bridge fragments. At the same time, it is obvious that the ring size can significantly influence the photochemical properties. In this regard, it seemed interesting to obtain terarylenes with a six-membered bridge and to study their behavior under the action of UV light. Continuing our research in the field of terarylenes containing a 3-hydroxy-4-pyranone fragment, in the present communication we investigated the photochemical properties of 2-(4-(4-aryl)-2-(dimethylamino)pyrimidin-5-yl)-6-methyl-4*H*-pyran-4-ones **9** and **10**. It is important to note that the 6π-electrocyclization of 1,3,5-hexatriene systems with a pyrimidine-bridge fragment has been described in the literature [26–28]. Herein, we show that for pyrimidines **9** and **10** containing an allomaltol fragment the main direction of a phototransformation is also the cyclization of the triene system. In this case, condensed dihydropyranone derivatives **11** were obtained along with the expected polyaromatic compounds **12** (Scheme 1D).

## Results and Discussion

The starting terarylenes **9** were obtained based on the reaction of the corresponding enaminones **13** with cyanamide (**14**). It should be noted that a similar approach is described in the literature and involves the condensation of 3-(dimethylamino)-1-phenylprop-2-en-1-one with cyanamide (**14**) in NMP in the presence of *N*-methylmorpholine [29]. However, this reaction led to a mixture of products and the yield of the target pyrimidine did not exceed 10%. It was assumed that the reported conditions were not optimal and the process could be significantly improved. In order to employ the aforementioned approach for the preparation of pyrimidines containing an allomaltol moiety we initially selected the interaction of enaminone **13a** with cyanamide as a model reaction. We varied time and temperature of the process, as well as basic reagents and solvents to identify the optimal conditions and the best results are summarized in Table 1.

**Table 1:** Optimization of the condensation conditions.^a^

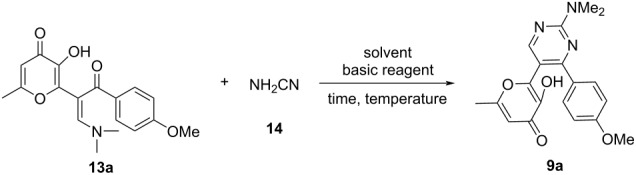					

Entry	Time, h	Solvent	Temperature, °C	Basic reagent	Yield, %

1	16	NMP	100	*N*-methylmorpholine	15
2	16	NMP	100	–	44
3	16	MeCN	reflux	–	52
4	16	toluene	reflux	–	40
5	16	EtOH	reflux	–	79
6	16	dioxane	reflux	–	62
7	16	MeOH	reflux	–	73
8	2	EtOH	reflux	–	80
9	2	EtOH	reflux	*N*-methylmorpholine	22
10	2	EtOH	reflux	Et_3_N	25
11	16	dioxane	rt	–	32
12^b^	16	EtOH	rt	–	–

^a^Reaction conditions: **13a** (1 mmol, 0.33 g), **14** (3 mmol, 0,13 g), solvent (5 mL), time, basic reagent (1 mmol), *T* in °C. ^b^**13a** is not soluble in EtOH at rt.

At first, based on literature data we carried out the reaction in NMP at 100 °C for 16 h in the presence of *N*-methylmorpholine. In this case the target pyrimidine **9a** was obtained in 15% yield (Table 1, entry 1). Next, we repeated the above-mentioned reaction under similar conditions without a basic reagent and the exclusion of the base led to a significant increase in the yield of the target product **9a** (Table 1, entry 2). Next, we tested the reaction in various solvents in the absence of a base (Table 1, entries 3–7) and the best results were obtained using alcoholic media (Table 1, entries 5 and 7). Probably, this is due to the protic nature of these solvents facilitating the studied condensation. Also, we have found that a decrease in the reaction time to 2 h did not affect the yield of pyrimidine **9a** (Table 1, entry 8). It is important to emphasize that the use of bases essentially impaired the efficiency of the method (Table 1, entries 9 and 10). Apparently, the addition of a basic reagent does not influence the main process and the decrease in the yield is connected to side reactions. It is also of note that an elevated reaction temperature is beneficial for the protocol (Table 1, entries 11 and 12). Thus, the highest yield of the target pyrimidine **9a** was obtained in refluxing EtOH for 2 h (Table 1, entry 8).

**Scheme 2:**
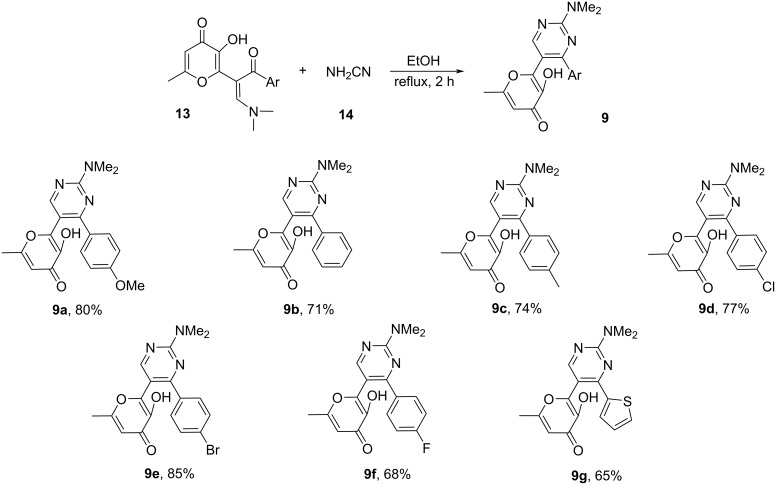
Synthesis of starting compounds **9**. Reaction conditions: **13** (1 mmol), NH_2_CN (**14**, 3 mmol, 0.13 g), EtOH (5 mL), reflux, 2 h.

Hence, optimal conditions were developed for this reaction, allowing for the synthesis of pyrimidines **9** containing an allomaltol unit in good yields (Scheme 2). The process is of a general nature and is suitable for the synthesis of various target products **9** with electron-rich or deficient substituents.

**Scheme 3:**
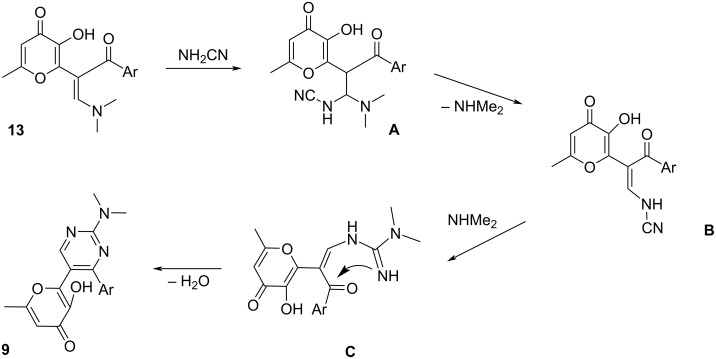
Proposed mechanism for the formation of compounds **9**.

The plausible reaction mechanism for the formation of pyrimidines **9** is presented in Scheme 3. At first, intermediate **A** is formed by Michael addition of cyanamide to enaminone **13**. Further elimination of dimethylamine leads to cyanoenaminone **B**. Next, interaction of the cyano group with dimethylamine results in the formation of guanidine **C**. Finally, the intramolecular cyclization of the guanidine moiety and the carbonyl group leads to the target pyrimidine **9**.

After the general synthetic method for pyrimidines containing the allomaltol fragment had been established, the photochemical behavior of the obtained compounds **9** was investigated.

**Scheme 4:**
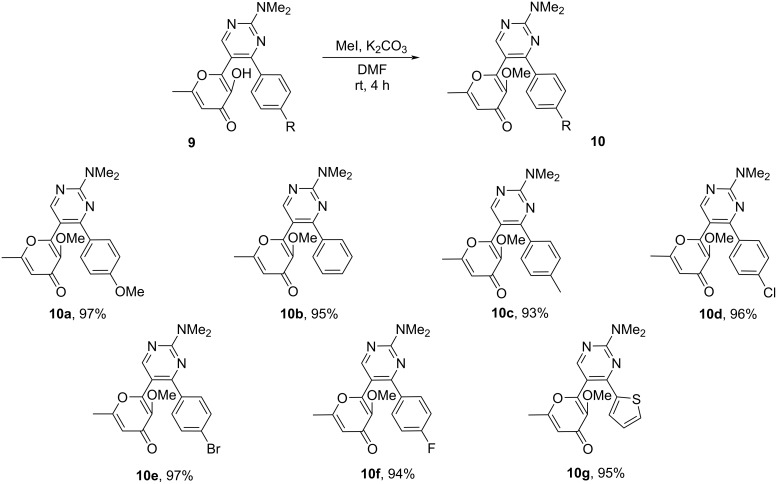
Synthesis of methylated derivatives **10**. Reaction conditions: **9** (1 mmol), MeI (3 mmol, 0.43 g), K_2_CO_3_ (3 mmol, 0.41 g), DMF (5 mL), stirring at rt for 4 h.

It should be noted that we have previously studied the photochemical properties of various terarylenes with a 3-hydroxy-4-pyranone moiety [21–25]. It was shown that UV irradiation of such systems leads to a complex mixture of products, apparently due to the simultaneous occurrence of a 6π-electrocyclization of the hexatriene system and ESIPT-induced contraction of the pyranone ring. At the same time, the blocking of the ESIPT-promoted process via alkylation of the hydroxy group allows one to realize the regiospecific cyclization of the triene system. Based on these previous results, we started the photochemistry study of the considered pyrimidines from the corresponding methylated derivatives of **9**. The products **10** were obtained by alkylation of compounds **9** with methyl iodide in DMF in the presence of K_2_CO_3_ (Scheme 4).

**Figure 1:**
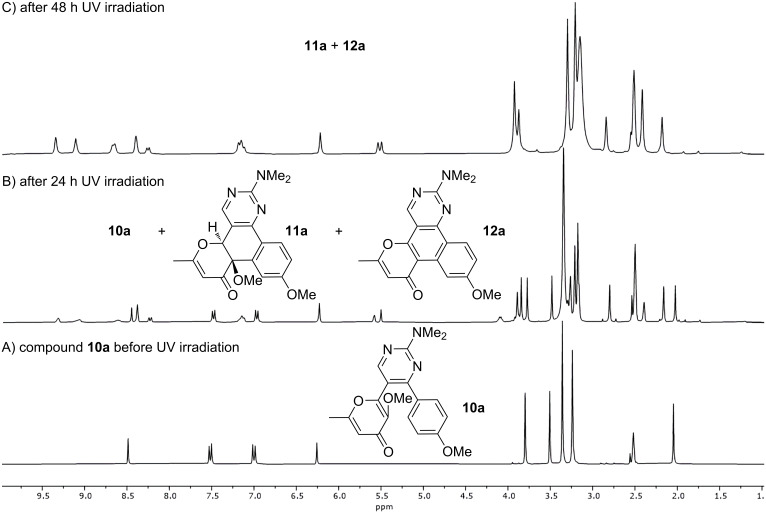
^1^H NMR monitoring of the photoreaction of compound **10a** under UV irradiation (365 nm) in DMSO-*d*_6_ solution.

**Figure 2:**
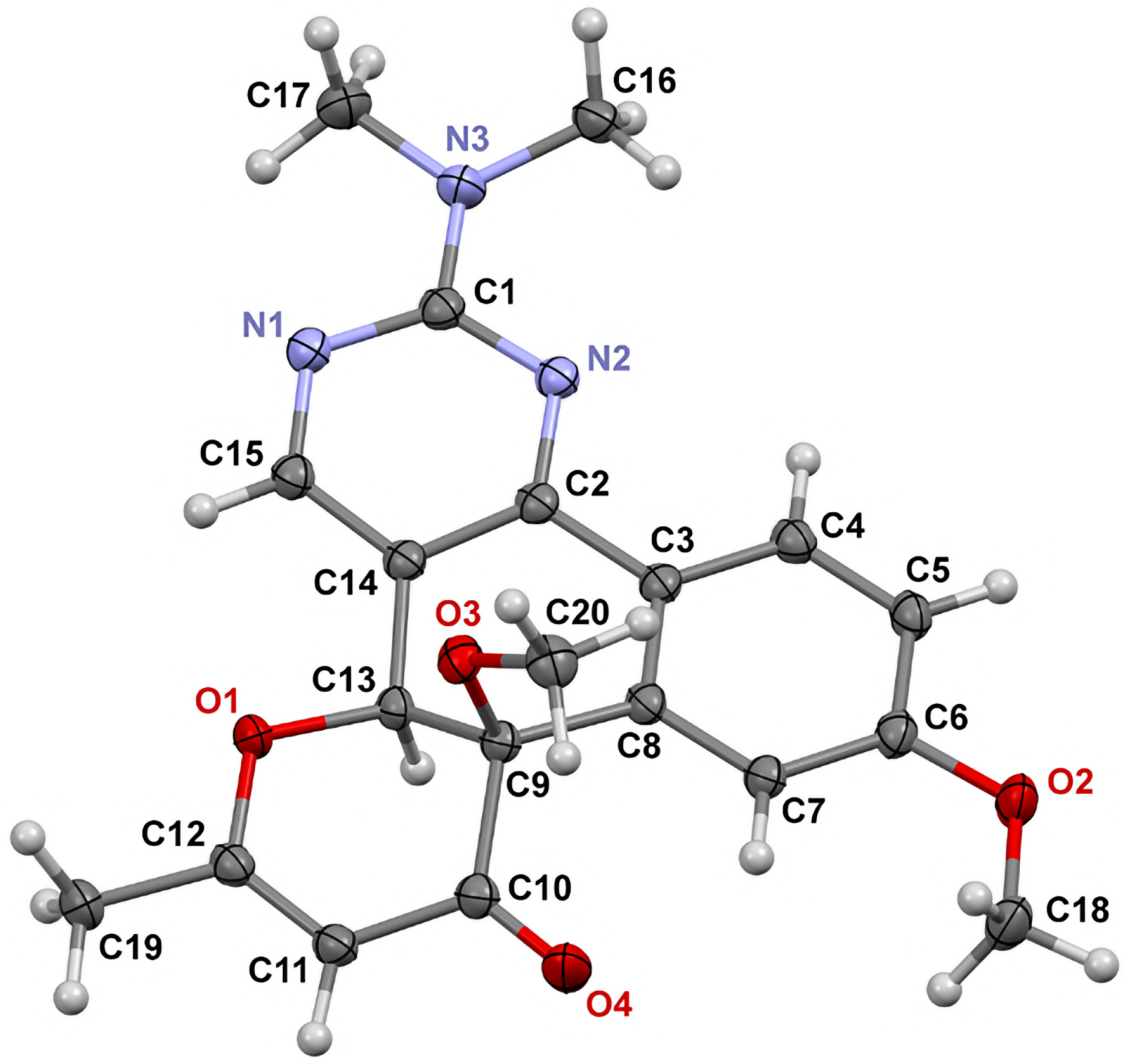
The crystal structure of compound **11a** (one of two polymorph modifications; *p* = 50%), CCDC 2248033.

**Scheme 5:**
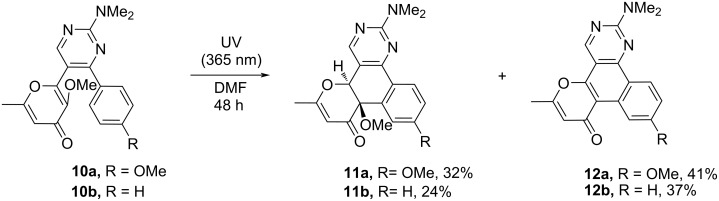
Photochemical synthesis of compounds **11** and **12**.

At first, the photochemical behavior was investigated for the model compound **10a** in DMSO-*d*_6_ solution using ^1^H NMR monitoring (Figure 1). Thus, the NMR spectrum recorded after UV irradiation (365 nm) for 24 h contained signals of protons of two products along with signals of the starting pyrimidine **10a** (Figure 1B). The complete conversion of the starting compound **10a** to the aforementioned mixture was observed after 48 h (Figure 1C). It should be noted that further irradiation of the test sample does not change the ratio of the formed products. In order to obtain the compounds in pure form we repeated the reaction in DMF irradiating at 365 nm for 48 h. As a result, both products could be isolated and characterized using ^1^H, ^13^C NMR spectroscopy and mass spectrometry. Moreover, the structure of product **11a** was also confirmed by single-crystal X-ray diffraction analysis. It might be noted that precipitated crystals of **11a** contained two polymorph modifications of triclinic (*P*1̄ and monoclinic (*P*2_1_/*c*) crystal systems (see Supporting Information File 1 for details); the structure of one modification is shown in Figure 2. Thus, the first obtained compound is the expected polycycle **12a**, while the second one is product **11a** formed as a result of 6π-electrocyclization and subsequent [1,9]-*H* sigmatropic shift (Scheme 5). The considered photoprocess is of a general nature and the formation of a similar mixture of products **11** and **12** is observed upon UV irradiation of all starting compounds **10**. Of note, the substituent in the aromatic ring influences only the ratio of the photoproducts (Table 2).

**Table 2:** Ratio of photoproducts **11** and **12**.^a^

Starting compound	Ratio of photoproducts **11** and **12**

**10a**	1:2
**10b**	1:2
**10c**	1:1.4
**10d**	1:1
**10e**	1:1
**10f**	1:1

^a^Ratio was calculated based on the ^1^H NMR spectrum of the crude reaction mixture.

Based on the structure of compound **11a** we assumed that it could be converted into a polyaromatic product using conventional synthetic methods. However, the use of different systems (TsOH/toluene, HCl/EtOH, DBU/EtOH, MeONa/MeOH) resulted only in the decomposition of the derivative **11a**. Thus, all our attempts towards the regiospecific photochemical conversion of pyrimidines **10** into polycyclic compounds **12** were unsuccessful.

**Scheme 6:**
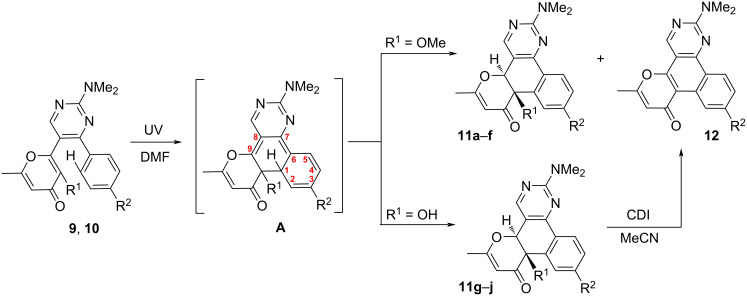
Proposed mechanism for the studied photoreaction.

A plausible mechanism for the considered phototransformation of compounds **9** and **10** is depicted in Scheme 6. At first, pyrimidines **10** undergo a 6π-electrocyclization under UV irradiation resulting in the formation of unstable intermediate **A**, starting from which two directions of further transformation are possible. The most obvious pathway is a simple elimination of a methanol molecule with aromatization of the central benzene ring leading to polycyclic product **12**. In addition to the considered variant thermal suprafacial [1,9]-*H* sigmatropic shift resulting in compound **11** is implemented in this case. This transformation is energetically favorable due to the restoration of aromaticity of the benzene and pyrimidine rings. At the same time, the elimination of the methanol molecule occurs only for intermediate **A** and is not observed for product **11**. It is important to emphasize that earlier, when discussing the mechanisms of the 6π-electrocyclization of various terarylenes accompanied by subsequent elimination of small molecules, we assumed a [1,9]-*H* sigmatropic shift step [23–25,30]. However, in all cases we failed to detect these intermediates even in the reaction mixture using NMR monitoring. However, in the present communication, for the first time, we managed to isolate and fully characterize a product of this class.

**Scheme 7:**
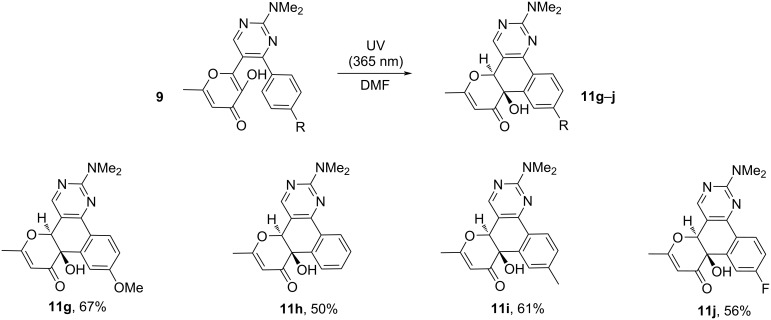
Synthesis of compounds **11g**–**j** starting from pyrimidines **9**. Reaction conditions: **9** (0.5 mmol), DMF (15 mL), irradiation with a Vilber Lourmat VL-6.LM lamp (365 nm, 6 W) for 96 h at 27 °C in a commercial 25 mL-round-bottomed glass flask.

Previously, we have shown that an alternative option for blocking ESIPT-induced processes for allomaltol derivatives is the use of DMF as a solvent [24]. Based on this fact, we assumed that the photoreaction in DMF would also allow the conversion of the starting pyrimidines **9** into the corresponding polycycles **12**. However, the UV irradiation of compound **9a** at 365 nm for 48 hours led only to product **11a** resulting from the aforementioned [1,9]-*H* sigmatropic shift (Scheme 7). In addition, a significant amount of unreacted starting pyrimidine **9a** was present in the reaction mixture. When increasing the process time to 96 h a complete conversion of substrate **9a** could be achieved. Note, the phototransformation in this case is accompanied by side reactions, which are apparently associated with incomplete suppression of ESIPT-induced processes. At the same time, the prolonged irradiation of the reaction mixture did not lead to the conversion of the compound **11a** into the polyaromatic product **12a** and only a slight decrease in the yield as a result of photodecomposition was observed. Thus, the use of the above-mentioned conditions (365 nm, DMF, 96 h) allows one to synthesize products **11** (Scheme 7).

**Figure 3:**
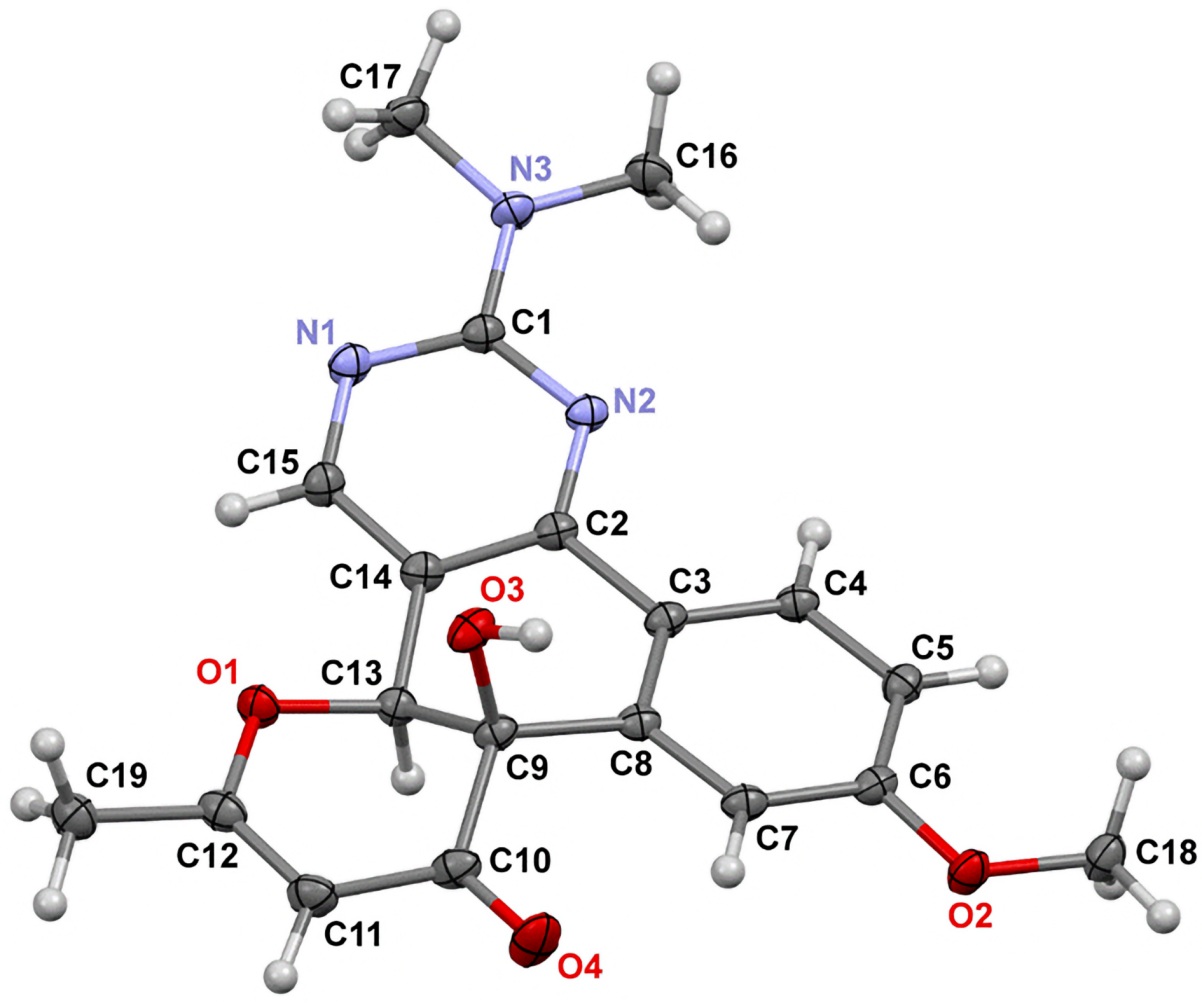
One of crystallographically unique molecules of **11g** (*p* = 50%), CCDC 2248035.

The structures of the obtained products **11g**–**j** were confirmed by ^1^H, ^13^C NMR spectroscopy and high-resolution mass spectrometry. In the ^1^H NMR spectra of the products, characteristic singlets corresponding to the protons of the dihydropyranone fragment in the region δ 5.3–5.4 ppm and the protons of the hydroxy group in the region δ 5.4–5.5 ppm are present. In addition to the aforementioned characterization methods, the crystal structure of **11g**·0.5EtOH was determined by single-crystal X-ray diffraction analysis. Its asymmetric unit contains 12 crystallographically unique molecules of **11g** (*Z*' = 12, *Z* = 24) and 6 independent ethanol molecules. One molecule of **11g** is shown in Figure 3. The other molecules have very similar conformations, but the methoxy group in some molecules exhibits an opposite orientation.

The mechanism of formation of products **11g**–**j** is similar to the presented mechanism above for the methoxy derivatives **11a**–**f** (Scheme 6). However, the key difference is the regioselectivity of the process and the absence of a pathway associated with the direct elimination of a water molecule and the formation of the polyaromatic compound **12**. It can be assumed that for the considered system a [1,9]-*H* sigmatropic shift is a much more preferable process compared to aromatization.

**Scheme 8:**
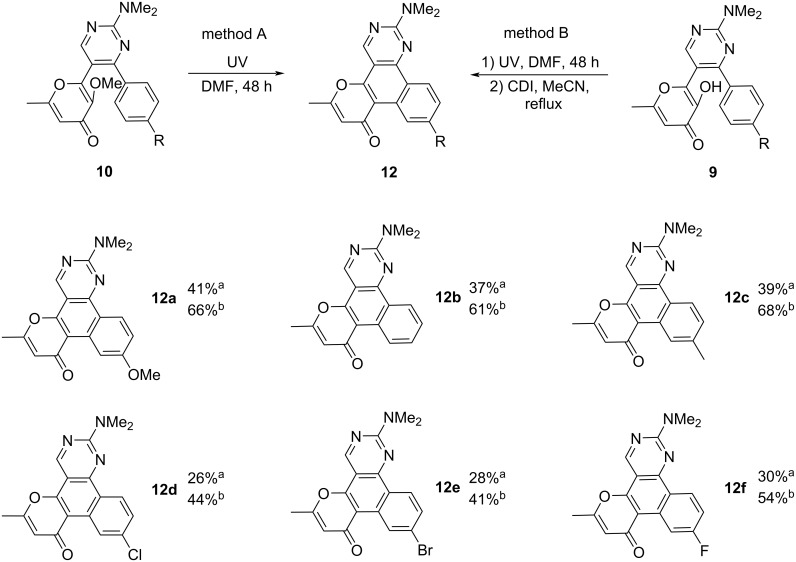
Synthesis of photoproducts **12**. Reaction conditions: method A) **10** (0.5 mmol), DMF (15 mL) irradiation for 48 h; method B) 1) **9** (0.5 mmol), DMF (15 mL), irradiation for 96 h; 2) CDI (1.75 mmol, 0.28 g), MeCN (5 mL), reflux, 2 h.

The above-mentioned photochemical reaction of pyrimidines **10** allows to synthesize polycyclic products **12** (Scheme 8, method A). In this case, the yields of compounds **12** did not exceed 41%, which is associated with low regioselectivity of the process and the simultaneous formation of the products **11a**–**f**. At the same time, we failed to increase the yield of the polycyclic products **12** by additional conversion of the byproducts **11a**–**f**. At this point, we assumed that the conversion of the analogous hydroxy derivatives **11g**–**j** into the polycyclic compounds **12** could be achieved by using suitable dehydrating agents (TsOH/toluene, Ac_2_O/MeCN, SOCl_2_/toluene, CDI/MeCN, POCl_3_/toluene). Gratifyingly, it was shown that the application of 1,1-carbonyldiimidazole (CDI) in acetonitrile allows to convert compound **11g** into the polyaromatic product **12a** in 94% yield (see Scheme S5 in Supporting Information File 1).

Based on the obtained results, we have developed a preparative telescopic method for the synthesis of polycycles **12**. The presented two-step one-pot approach includes the preliminary photoconversion of the starting pyrimidines **9** in DMF to the corresponding hydroxy intermediates **11** and subsequent dehydration using the CDI/acetonitrile system (Scheme 8, method B). This approach allowed to increase the yield of products **12** in comparison with the phototransformation of methylated derivatives **10** (method A in Scheme 8).

It should be mentioned that the presented photoreaction can be carried out for starting compounds with both donor and acceptor substituents in the aryl fragment. However, it is interesting to note that the UV irradiation of thiophene containing pyrimidines **9g** and **10g** leads to a complicated mixture of unidentified products. Probably, this is due to photochemical side processes involving the thiophene ring.

## Conclusion

In summary we have developed an approach for the preparation of pyrimidines with an allomaltol fragment. The suggested method started with the synthesis of pyrimidines from the reaction of 2-(1-(dimethylamino)-3-oxo-3-arylprop-1-en-2-yl)-3-hydroxy-6-methyl-4*H*-pyran-4-ones with cyanamide. Subsequently, the photochemical properties of synthesized pyrimidines were studied. We have demonstrated that a 6π-electrocyclization of the 1,3,5-hexatriene system and subsequent [1,9]-*H* sigmatropic shift leading to dihydrobenzo[*h*]pyrano[2,3-*f*]quinazolines are realized for hydroxy derivatives under UV irradiation. At the same time, for similar allomaltols with a methoxy group the photoprocess results in the formation of a mixture of the polyaromatic compounds and the corresponding dihydrobenzo[*h*]pyrano[2,3-*f*]quinazolines. The prepared dihydro derivatives are stable products and do not convert into polyaromatic benzo[*h*]pyrano[2,3-*f*]quinazolines upon further UV irradiation. The structures of two of the dihydrobenzo[*h*]pyrano[2,3-*f*]quinazolines were confirmed by X-ray diffraction analysis. Based on the obtained results, a two-step one-pot protocol for the preparation of polycyclic benzo[*h*]pyrano[2,3-*f*]quinazolines was elaborated. The method comprises the preliminary photoreaction of the starting compounds and the conclusive dehydration under the action of CDI.

## Supporting Information

File 1 Experimental procedures, characterization data of all products, copies of ^1^H, ^13^C NMR, HRMS spectra of all new compounds, and X-ray crystallographic data.
