# Supplementary material for: Synthesis of substituted 8H-benzo[h]pyrano[2,3-f]quinazolin-8-ones via photochemical 6π-electrocyclization of pyrimidines containing an allomaltol fragment
Source: Beilstein J Org Chem. 2023 Jun 7;19:778–88. doi: 10.3762/bjoc.19.58 (PMC10280060; doi:10.3762/bjoc.19.58)
Supplement: File 1 — Experimental procedures, characterization data of all products, copies of 1H, 13C NMR, HRMS spectra of all new compounds, and X-ray crystallographic data. [file Beilstein_J_Org_Chem-19-778-s001.pdf]

BEILSTEIN JOURNAL OF ORGANIC CHEMISTRY

Supporting Information

for

Synthesis of substituted 8*H*-benzo[*h*]pyrano[2,3-*f*]quinazolin-8-ones via photochemical 6π-electrocyclization of pyrimidines containing an allomaltol fragment

Constantine V. Milyutin, Andrey N. Komogortsev, Boris V. Lichitsky, Mikhail E. Minyaev and Valeriya G. Melekhina

Beilstein J. Org. Chem. **2023,** *19,* 778–788. doi:10.3762/bjoc.19.58

Experimental procedures, characterization data of all products, copies of ^{1}H, ^{13}C NMR, HRMS spectra of all new compounds, and X-ray crystallographic data

License and Terms: This is a supporting information file under the terms of the Creative Commons Attribution License (https://creativecommons.org/licenses/by/4.0). Please note that the reuse, redistribution and reproduction in particular requires that the author(s) and source are credited and that individual graphics may be subject to special legal provisions.
The license is subject to the *Beilstein Journal of Organic Chemistry* terms and conditions: (https://www.beilstein-journals.org/bjoc/terms)

Table of contents

1. General information S2

2. Characterization data of starting compounds **9** and **10** S5

3. Characterization data of photoproducts **11** and **12**. S8

4. ^{1}H and ^{13}C NMR spectra for starting compounds **9** and **10** S11

5. ^{1}H and ^{13}C NMR spectra for photoproducts **11** and **12** S25

6. X-ray crystallographic data and refinement details S37

7. References S43

1. General information

General information. Unless otherwise stated, all starting chemicals were commercially available and were used as received. The starting compounds **13** were prepared to a procedure described in the literature.[1] NMR spectra were recorded with Bruker AM 300 (300 MHz), Bruker AV 400 (400 MHz), Bruker DRX 500 (500 MHz), and Bruker AV 600 (600 MHz) spectrometers in DMSO-d_6 and $CDCl_3$. Chemical shifts (ppm) are given relative to solvent signals (DMSO-d_6: 2.50 ppm (^1H NMR) and 39.52 ppm (^{13}C NMR); $CDCl_3$: 7.26 ppm (^1H NMR) and 77.16 ppm (^{13}C NMR)). High-resolution mass spectra (HRMS) were obtained on a Bruker micrOTOF II instrument using electrospray ionization (ESI). The melting points were determined on a Kofler hot stage. A magnetic stirrer IKA C-MAG HS 7 was used for the reactions that require heating.

UV irradiation was carried out with a Vilber Lourmat VL-6.LM lamp (365 nm). Photochemical reactions were performed in commercial 25 mL round-bottomed glass flasks at 27 °C. The distance from the light source to the irradiation vessel was 4 cm.

General experimental procedure for the synthesis of pyrimidines 9.

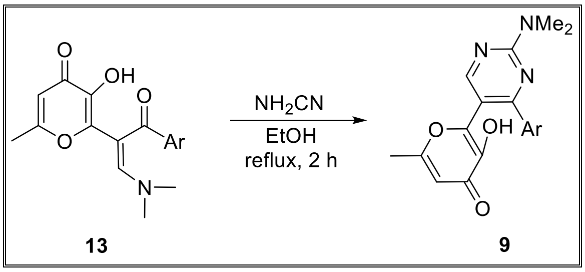

Scheme S1.

A mixture of compound **13** (1 mmol) and cyanamide (3 mmol, 0.13 g) in EtOH (5 mL) was refluxed for 2 h. The reaction mixture was cooled to room temperature, and the formed precipitate was filtered off and washed with EtOH (3 × 5 mL).

Experimental procedure for the synthesis of methylated derivatives 10.

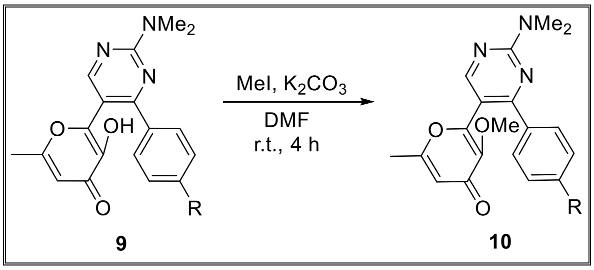

Scheme S2.

A mixture of the corresponding pyrimidine **9** (1 mmol), K_2CO_3 (3 mmol, 0.41 g) and MeI (3 mmol, 0.43 g) in DMF (5 mL) was stirred for 4 h at room temperature. Then, the solvent was removed under reduced pressure, H_2O (20 mL) was added to the residue, and the mixture was left standing overnight. The resulting precipitate was filtered off and washed with H_2O (3 × 10 mL).

Experimental procedure for the photochemical synthesis of compounds 11a,b and 12a,b from pyrimidines 10a,b.

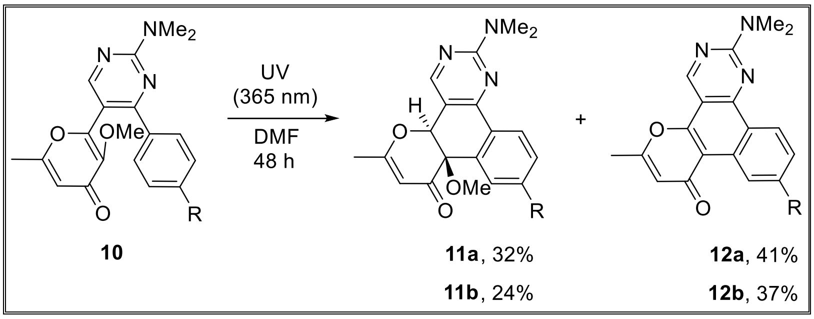

Scheme S3.

A solution of compound **10a**,**b** (0.5 mmol) in DMF (15 mL) was irradiated in a commercial 25 mL round-bottomed glass flask with a Vilber Lourmat VL-6.LM (365 nm, 6 W) for 48 h. Then, the solvent was removed under reduced pressure and the residue was recrystallized from MeCN (3 mL). The product **12a**,**b** was filtered off and washed with MeCN (3 × 5 mL). After the separation of compound **12a**,**b**, the saturated solution was concentrated in vacuo and the precipitate was recrystallized from EtOH (2 mL). The obtained product **11a**,**b** was filtered off and washed with EtOH (3 × 3 mL).

General experimental procedure for the synthesis of photoproducts 11g–j from 9.

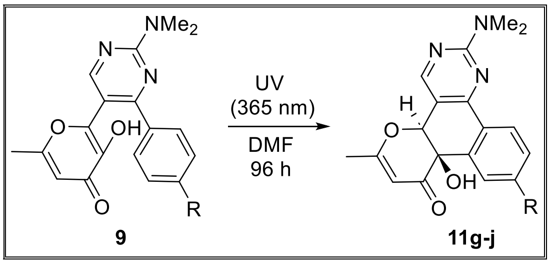

Scheme S4.

A solution of the corresponding compound **9** (0.5 mmol) in DMF (15 mL) was irradiated in a commercial 25 mL round-bottomed glass flask with a Vilber Lourmat VL-6.LM (365 nm, 6 W) for 96 h. Then, the solvent was removed under reduced pressure and the residue was recrystallized from EtOH (3 mL) for compounds **11g**,**i**. Compounds **11h** and **11j** were purified by column chromatography on silica gel (*n*-hexane/EtOAc 1:3).

Experimental procedure for the synthesis of photoproduct 12a from compounds 11g

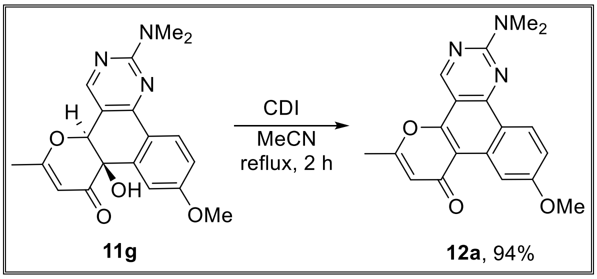

Scheme S5.

The mixture of compound **11g** (1 mmol, 0.35 g) and CDI (3 mmol, 0.49 g) in MeCN (6 mL) was refluxed for 2 h. The reaction mixture was cooled to room temperature and the resulting precipitate was filtered off and washed MeCN (3 × 10 mL).

Experimental procedure for the synthesis of photoproducts 12 from compounds 10 (method A).

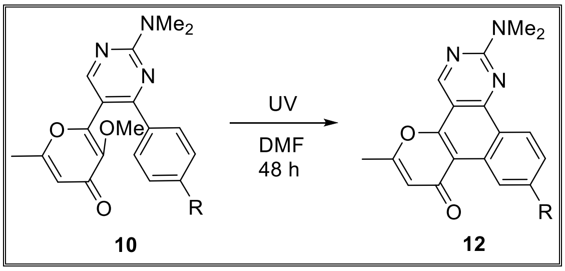

Scheme S6.

A solution of the corresponding compound **10** (0.5 mmol) in DMF (15 mL) was irradiated in a commercial 25 mL round-bottomed glass flask with a Vilber Lourmat VL-6.LM (365 nm, 6 W) for 48 h. Then, the solvent was removed under reduced pressure and the residue was recrystallized from MeCN (5 mL). The obtained product **12** was filtered off and washed with MeCN (3 × 5 mL).

General experimental procedure for the synthesis of photoproducts 12 from compounds 9 (method B).

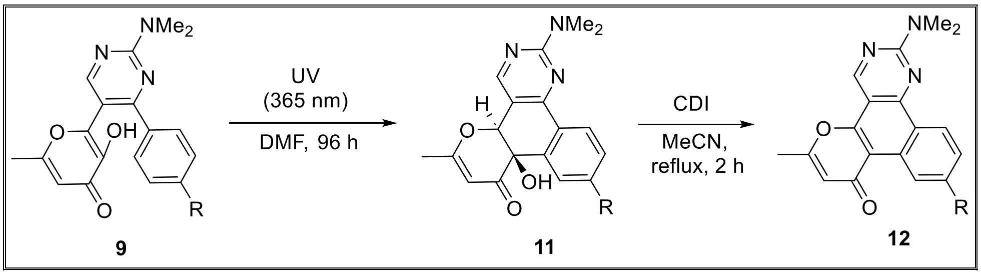

Scheme S7.

A solution of the corresponding compound **9** (0.5 mmol) in DMF (15 mL) was irradiated in a commercial 25-mL round-bottomed glass flask with a Vilber Lourmat VL-6.LM (365 nm, 6 W) for 96 h. Then, the solvent was removed under reduced pressure. To the obtained residue CDI (1.75 mmol, 0.28 g) and MeCN (5 mL) were added, and the obtained solution was refluxed for 2 h. The resulting solution was cooled to room temperature and the formed precipitate was filtered off and washed MeCN (3 × 10 mL).

2. Characterization data of starting compounds 9 and 10

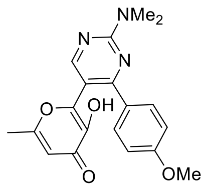

*2-(2-(Dimethylamino)-4-(4-methoxyphenyl)pyrimidin-5-yl)-3-hydroxy-6-methyl-4H-pyran-4-one (**9a**)*

White powder; yield 80% (0.28 g); mp 236-238 °C. ^{1}H NMR (300 MHz, DMSO-d_6) δ 8.93 (br.s, 1H), 8.49 (s, 1H), 7.49 (d, *J* = 8.8 Hz, 2H), 6.95 (d, *J* = 8.8 Hz, 2H), 6.24 (s, 1H), 3.77 (s, 3H), 3.22 (s, 6H), 1.98 (s, 3H). ^{13}C NMR (75 MHz, DMSO-d_6) δ 173.58, 164.45, 163.00, 161.34, 160.51, 159.64, 145.26, 142.10, 130.55, 129.46, 113.56, 111.25, 109.66, 55.24, 36.63, 19.03. HRMS (ESI-TOF) *m/z*: [M+H]$^+$ Calcld for $C_{19}H_{20}N_3O_4$: 354.1454; Found: 354.1468.

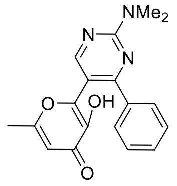

*2-(2-(Dimethylamino)-4-phenylpyrimidin-5-yl)-3-hydroxy-6-methyl-4H-pyran-4-one (**9b**)*

White powder; yield 71% (0.23 g); mp 175-177 °C. ^{1}H NMR (300 MHz, DMSO-d_6) δ 8.99 (s, 1H), 8.57 (s, 1H), 7.54 – 7.32 (m, 5H), 6.20 (s, 1H), 3.22 (s, 6H), 1.87 (s, 3H). ^{13}C NMR (75 MHz, DMSO-d_6) δ 173.59, 164.32, 163.86, 161.34, 159.64, 144.84, 142.16, 138.54, 129.46, 128.12, 127.78, 111.19, 110.19, 36.70, 18.86. HRMS (ESI-TOF) *m/z*: [M+H]$^+$ Calcld for $C_{18}H_{18}N_3O_3$: 324.1348; Found: 324.1345.

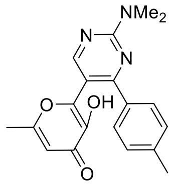

*2-(2-(Dimethylamino)-4-(p-tolyl)pyrimidin-5-yl)-3-hydroxy-6-methyl-4H-pyran-4-one (**9c**)*

White powder; yield 74% (0.25 g); mp 234-236 °C. ^{1}H NMR (300 MHz, DMSO-d_6) δ 8.93 (br.s, 1H), 8.52 (s, 1H), 7.40 (d, *J* = 7.9 Hz, 2H), 7.20 (d, *J* = 7.9 Hz, 2H), 6.21 (s, 1H), 3.21 (s, 6H), 2.32 (s, 3H), 1.93 (s, 3H). ^{13}C NMR (75 MHz, DMSO-d_6) δ 173.52, 164.35, 163.65, 161.34, 159.55, 145.04, 142.10, 139.21, 135.60, 128.62, 127.73, 111.15, 109.98, 36.61, 20.80, 18.90. HRMS (ESI-TOF) *m/z*: [M+H]$^+$ Calcld for $C_{19}H_{20}N_3O_3$: 338.1504; Found: 338.1516.

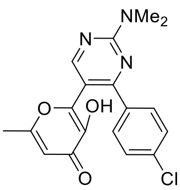

*2-(4-(4-Chlorophenyl)-2-(dimethylamino)pyrimidin-5-yl)-3-hydroxy-6-methyl-4H-pyran-4-one (**9d**)*

White powder; yield 77% (0.27 g); mp 204-206 °C. ^{1}H NMR (300 MHz, DMSO-d_6) δ 8.99 (br.s, 1H), 8.57 (s, 1H), 7.51 (d, *J* = 8.8 Hz, 2H), 7.47 (d, *J* = 8.9 Hz, 2H), 6.22 (s, 1H), 3.22 (s, 6H), 1.96 (s, 3H). ^{13}C NMR (75 MHz, DMSO-d_6) δ 173.63, 164.59, 162.67, 161.34, 159.79, 144.60, 142.15, 137.34, 134.43, 129.67, 128.30, 111.30, 110.11, 36.75, 18.99. HRMS (ESI-TOF) *m/z*: [M+H]$^+$ Calcld for $C_{18}H_{17}ClN_3O_3$: 358.0958; Found: 358.0969.

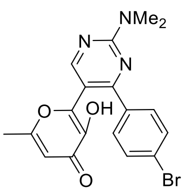

*2-(4-(4-Bromophenyl)-2-(dimethylamino)pyrimidin-5-yl)-3-hydroxy-6-methyl-4H-pyran-4-one (**9e**)*

White powder; yield 85% (0.34 g); mp 211-213 °C. ^{1}H NMR (300 MHz, DMSO-d_6) δ 8.98 (br.s, 1H), 8.57 (s, 1H), 7.61 (d, *J* = 8.3 Hz, 2H), 7.44 (d, *J* = 8.3 Hz, 2H), 6.22 (s, 1H), 3.22 (s, 6H), 1.96 (s, 3H). ^{13}C NMR (75 MHz, DMSO-d_6) δ 173.54, 164.43, 162.64, 161.28, 159.78, 144.46, 142.13, 137.69, 131.18, 129.87, 123.11, 111.29, 110.04, 36.69, 18.95. HRMS (ESI-TOF) *m/z*: [M+H]$^+$ Calcld for $C_{18}H_{17}BrN_3O_3$: 402.0453; Found: 402.0447.

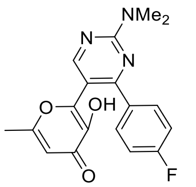

*2-(2-(Dimethylamino)-4-(4-fluorophenyl)pyrimidin-5-yl)-3-hydroxy-6-methyl-4H-pyran-4-one (**9f**)*

Grey powder; yield 68% (0.23 g); mp 136-138 °C. ^{1}H NMR (300 MHz, DMSO-d_6) δ 8.99 (s, 1H), 8.56 (s, 1H), 7.62 – 7.49 (m, 2H), 7.32 – 7.18 (m, 2H), 6.22 (s, 1H), 3.22 (s, 6H), 1.95 (s, 3H). ^{13}C NMR (75 MHz, DMSO-d_6) δ 173.57, 164.46, 162.9 (d, *J* = 241.4 Hz), 161.30 161.22, 159.73, 144.68, 142.14, 134.89 (d, *J* = 3.1 Hz), 130.13 (d, *J* = 8.6 Hz), 115.13 (d, *J* = 21.7 Hz), 111.24, 110.03, 36.69, 18.95. HRMS (ESI-TOF) *m/z*: [M+H]$^+$ Calcld for $C_{18}H_{17}FN_3O_3$: 342.1254; Found: 342.1270.

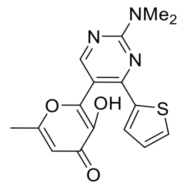

*2-(2-(Dimethylamino)-4-(thiophen-2-yl)pyrimidin-5-yl)-3-hydroxy-6-methyl-4H-pyran-4-one (**9g**)*

Grey powder; yield 65% (0.21 g); mp 217-219 °C. ^{1}H NMR (300 MHz, DMSO-d_6) δ 9.10 (br.s, 1H), 8.40 (s, 1H), 7.75 (d, *J* = 4.9 Hz, 1H), 7.21 – 7.15 (m, 1H), 7.14 – 7.07 (m, 1H), 6.38 (s, 1H), 3.21 (s, 6H), 2.21 (s, 3H). ^{13}C NMR (75 MHz, DMSO-d_6) δ 173.76, 165.15, 161.08, 160.45, 156.16, 144.38, 143.23, 141.67, 130.87, 128.76, 128.46, 111.79, 107.56, 36.53, 19.27. HRMS (ESI-TOF) *m/z*: [M+H]$^+$ Calcld for $C_{16}H_{16}N_3O_3S$: 330.0912; Found: 330.0929.

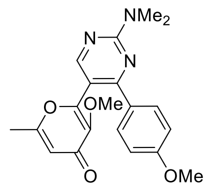

*2-(2-(Dimethylamino)-4-(4-methoxyphenyl)pyrimidin-5-yl)-3-methoxy-6-methyl-4H-pyran-4-one (**10a**)*

White powder; yield 97% (0.36 g); mp 140-142 °C. ^{1}H NMR (300 MHz, DMSO-d_6) δ 8.47 (s, 1H), 7.50 (d, *J* = 8.3 Hz, 2H), 6.98 (d, *J* = 8.4 Hz, 2H), 6.24 (s, 1H), 3.78 (s, 3H), 3.49 (s, 3H), 3.22 (s, 6H), 2.03 (s, 3H). ^{13}C NMR (75 MHz, DMSO-d_6) δ 174.72, 164.74, 163.37, 161.44, 160.71, 159.36, 154.69, 143.69, 130.29, 129.57, 114.32, 113.72, 109.33, 58.85, 55.29, 36.65, 18.83. HRMS (ESI-TOF) *m/z*: [M+H]$^+$ Calcld for $C_{20}H_{22}N_3O_4$: 368.1610; Found: 368.1601.

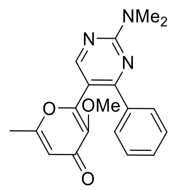

*2-(2-(Dimethylamino)-4-phenylpyrimidin-5-yl)-3-methoxy-6-methyl-4H-pyran-4-one (**10b**)*

Brown powder; yield 95% (0.32 g); mp 103-105 °C. ^{1}H NMR (300 MHz, DMSO-d_6) δ 8.54 (s, 1H), 7.55 – 7.38 (m, 5H), 6.21 (s, 1H), 3.49 (s, 3H), 3.23 (s, 6H), 1.95 (s, 3H). ^{13}C NMR (126 MHz, DMSO-d_6) δ 174.95, 164.90, 164.42, 161.57, 159.49, 154.45, 143.82, 138.33, 129.90, 128.43, 127.99, 114.33, 109.91, 59.01, 36.83, 18.79. HRMS (ESI-TOF) *m/z*: [M+H]$^+$ Calcld for $C_{19}H_{20}N_3O_3$: 338.1505; Found: 338.1512.

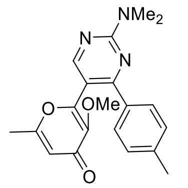

*2-(2-(Dimethylamino)-4-(p-tolyl)pyrimidin-5-yl)-3-methoxy-6-methyl-4H-pyran-4-one (**10c**)*

White powder; yield 93% (0.33 g); mp 127-129 °C. ^{1}H NMR (300 MHz, DMSO-d_6) δ 8.50 (s, 1H), 7.41 (d, *J* = 7.6 Hz, 2H), 7.23 (d, *J* = 7.8 Hz, 2H), 6.22 (s, 1H), 3.48 (s, 3H), 3.23 (s, 6H), 2.33 (s, 3H), 1.99 (s, 3H). ^{13}C NMR (75 MHz, DMSO-d_6) δ 174.79, 164.77, 161.50, 159.33, 154.54, 143.74, 139.64, 135.38, 128.86, 127.88, 114.26, 109.70, 96.37, 58.89, 36.70, 20.87, 18.77. HRMS (ESI-TOF) *m/z*: [M+H]$^+$ Calcld for $C_{20}H_{22}N_3O_3$: 352.1661; Found: 352.1660.

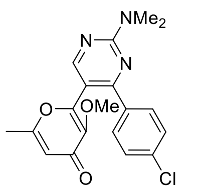

*2-(4-(4-Chlorophenyl)-2-(dimethylamino)pyrimidin-5-yl)-3-methoxy-6-methyl-4H-pyran-4-one (**10d**)*

Pale yellow powder; yield 96% (0.36 g); mp 102-104 °C. ^{1}H NMR (300 MHz, DMSO-d_6) δ 8.55 (s, 1H), 7.58 – 7.46 (m, 4H), 6.23 (s, 1H), 3.52 (s, 3H), 3.23 (s, 6H), 1.99 (s, 3H). ^{13}C NMR (75 MHz, DMSO-d_6) δ 174.61, 164.60, 162.88, 161.36, 159.53, 153.76, 143.70, 136.97, 134.57, 129.66, 128.37, 114.26, 109.61, 58.92, 36.65, 18.67. HRMS (ESI-TOF) *m/z*: $[M+H]^+$ Calcld for $C_{19}H_{19}ClN_3O_3$: 372.1114; Found: 372.1120.

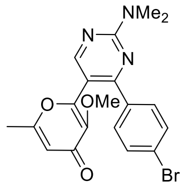

*2-(4-(4-Bromophenyl)-2-(dimethylamino)pyrimidin-5-yl)-3-methoxy-6-methyl-4H-pyran-4-one (**10e**)*

Pale yellow powder; yield 97% (0.4 g); mp 105-107 °C. ^{1}H NMR (300 MHz, DMSO-d_6) δ 8.55 (s, 1H), 7.64 (d, *J* = 8.4 Hz, 2H), 7.45 (d, *J* = 8.5 Hz, 2H), 6.23 (s, 1H), 3.52 (s, 3H), 3.22 (s, 6H), 1.99 (s, 3H). ^{13}C NMR (75 MHz, DMSO-d_6) δ 174.64, 164.62, 162.99, 161.37, 159.57, 153.78, 143.70, 137.38, 131.33, 129.92, 123.32, 114.31, 109.59, 58.94, 36.67, 18.70. HRMS (ESI-TOF) *m/z*: $[M+H]^+$ Calcld for $C_{19}H_{19}BrN_3O_3$: 416.0610; Found: 416.0609.

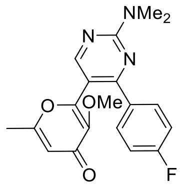

*2-(2-(Dimethylamino)-4-(4-fluorophenyl)pyrimidin-5-yl)-3-methoxy-6-methyl-4H-pyran-4-one (**10f**)*

Pale yellow powder; yield 94% (0.33 g); mp 123-125 °C. ^{1}H NMR (300 MHz, DMSO-d_6) δ 8.53 (s, 1H), 7.64 – 7.53 (m, 2H), 7.34 – 7.22 (m, 2H), 6.23 (s, 1H), 3.52 (s, 3H), 3.23 (s, 6H), 1.99 (s, 3H). ^{13}C NMR (75 MHz, DMSO-d_6) δ 174.70, 164.7, 163.01, 162.6 (d, *J* = 247.6 Hz), 161.4, 159.54, 158.12, 154.03, 143.74, 134.62 (d, *J* = 3.0 Hz), 130.26 (d, *J* = 8.8 Hz), 115.34 (d, *J* = 21.8 Hz), 114.31, 109.62, 58.95, 36.70, 18.74. HRMS (ESI-TOF) *m/z*: $[M+H]^+$ Calcld for $C_{19}H_{19}FN_3O_3$: 356.1410; Found: 356.1410.

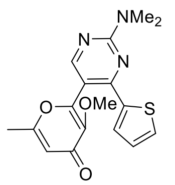

*2-(2-(Dimethylamino)-4-(thiophen-2-yl)pyrimidin-5-yl)-3-methoxy-6-methyl-4H-pyran-4-one (**10g**)*

Pale yellow powder; yield 95% (0.33 g); mp 112-114 °C. ^{1}H NMR (300 MHz, DMSO-d_6) δ 8.43 (s, 1H), 7.78 (d, *J* = 4.9 Hz, 1H), 7.21 – 7.10 (m, 2H), 6.39 (s, 1H), 3.58 (s, 3H), 3.22 (s, 6H), 2.20 (s, 3H). ^{13}C NMR (126 MHz, DMSO-d_6) δ 175.11, 165.68, 161.32, 160.05, 156.52, 154.38, 145.07, 141.46, 131.18, 128.99, 128.70, 114.95, 107.44, 59.57, 36.70, 19.15. HRMS (ESI-TOF) *m/z*: $[M+H]^+$ Calcld for $C_{17}H_{18}N_3O_3S$: 344.1069; Found: 344.1070.

3. Characterization data of photoproducts 11 and 12.

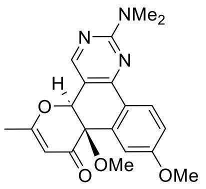

2-*(Dimethylamino)-8a,10-dimethoxy-6-methyl-4b,8a-dihydro-8H-benzo[h]pyrano[2,3-f]quinazolin-8-one **(11a)***

Pale yellow powder; yield 32% (0.06 g); mp 144-146 °C. ^{1}H NMR (300 MHz, DMSO-d_6) δ 8.43 (s, 1H), 8.41 (d, *J* = 2.7 Hz, 1H), 8.28 (d, *J* = 8.7 Hz, 1H), 7.16 (dd, *J* = 8.7, 2.7 Hz, 1H), 5.65 (s, 1H), 5.52 (s, 1H), 3.85 (s, 3H), 3.19 (s, 6H), 2.82 (s, 3H), 2.17 (s, 3H). ^{13}C NMR (75 MHz, DMSO-d_6) δ 188.70, 172.07, 161.84, 160.45, 156.25, 153.71, 133.96, 127.59, 125.49, 116.82, 114.35, 110.08, 104.18, 78.97, 69.69, 55.45, 51.67, 36.69, 20.05. HRMS (ESI-TOF) *m/z*: $[M+H]^+$ Calcld for $C_{20}H_{22}N_3O_4$: 368.1610; Found: 368.1623.

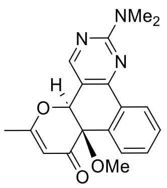

*2-(Dimethylamino)-8a-methoxy-6-methyl-4b,8a-dihydro-8H-benzo[h]pyrano[2,3-f]quinazolin-8-one (**11b**)*

Pale yellow powder; yield 24% (0.04 g); mp 128-130 °C ^{1}H NMR (300 MHz, $CDCl_3$) δ 8.89 (d, *J* = 7.0 Hz, 1H), 8.61 (s, 1H), 8.44 (d, *J* = 7.3 Hz, 1H), 7.58 – 7.44 (m, 2H), 5.48 (s, 1H), 5.45 (s, 1H), 3.29 (s, 6H), 2.95 (s, 3H), 2.20 (s, 3H). ^{13}C NMR (75 MHz, $CDCl_3$) δ 189.40, 171.78, 162.36, 157.34, 154.34, 133.69, 132.43, 131.16, 130.33, 129.85, 126.40, 110.46, 105.06, 80.11, 70.66, 52.66, 37.35, 20.73. HRMS (ESI-TOF) *m/z*: $[M+H]^+$ Calcld for $C_{19}H_{20}N_3O_3$: 338.1505; Found: 338.1527.

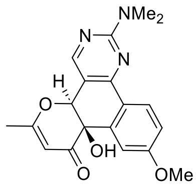

*2-(Dimethylamino)-8a-hydroxy-10-methoxy-6-methyl-4b,8a-dihydro-8H-benzo[h]pyrano[2,3-f]quinazolin-8-one (**11g**)*

White powder; yield 67% (0.12 g); mp 189-191 °C. ^{1}H NMR (300 MHz, DMSO-d_6) δ 8.52 – 8.39 (m, 2H), 8.24 (d, *J* = 8.6 Hz, 1H), 7.09 (dd, *J* = 8.7, 2.7 Hz, 1H), 6.28 (s, 1H), 5.47 (s, 1H), 5.41 (s, 1H), 3.84 (s, 3H), 3.20 (s, 6H), 2.16 (s, 3H). ^{13}C NMR (75 MHz, DMSO-d_6) δ 191.28, 171.51, 161.76, 160.93, 156.49, 153.77, 138.63, 126.99, 124.97, 114.85, 113.94, 110.16, 104.25, 79.31, 65.19, 55.35, 36.69, 20.06. HRMS (ESI-TOF) *m/z*: $[M+H]^+$ Calcld for $C_{19}H_{20}N_3O_4$: 354.1453; Found: 354.1455.

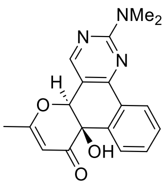

*2-(Dimethylamino)-8a-hydroxy-6-methyl-4b,8a-dihydro-8H-benzo[h]pyrano[2,3-f]quinazolin-8-one (**11h**)*

Yellow powder; yield 50% (0.08 g); mp 163-165 °C. ^{1}H NMR (300 MHz, $CDCl_3$) δ 8.92 – 8.80 (m, 1H), 8.47 (s, 1H), 8.39 – 8.30 (m, 1H), 7.60 – 7.43 (m, 2H), 5.49 (s, 1H), 5.33 (s, 1H), 3.05 (s, 6H), 2.20 (s, 3H). ^{13}C NMR (75 MHz, $CDCl_3$) δ 190.94, 172.04, 136.15, 131.78, 129.66, 129.43, 126.26, 110.46, 105.08, 79.73, 66.78, 37.28, 29.82, 20.77. HRMS (ESI-TOF) *m/z*: $[M+H]^+$ Calcld for $C_{18}H_{18}N_3O_3$: 324.1348; Found: 324.1331.

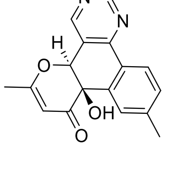

*2-(Dimethylamino)-8a-hydroxy-6,10-dimethyl-4b,8a-dihydro-8H-benzo[h]pyrano[2,3-f]quinazolin-8-one (**11i**)*

Yellow powder; yield 61% (0.1 g); mp 221-223 °C. ^{1}H NMR (300 MHz, DMSO-d_6) δ 8.64 (s, 1H), 8.48 (s, 1H), 8.21 (d, *J* = 7.9 Hz, 1H), 7.34 (d, *J* = 7.9 Hz, 1H), 6.23 (s, 1H), 5.47 (s, 1H), 5.44 (s, 1H), 3.21 (s, 6H), 2.39 (s, 3H), 2.16 (s, 3H). ^{13}C NMR (75 MHz, DMSO-d_6) δ 191.22,

171.35, 161.81, 156.99, 154.05, 140.37, 136.86, 129.73, 129.45, 125.27, 110.72, 104.32, 79.38, 65.47, 36.70, 21.62, 20.04. HRMS (ESI-TOF) *m/z*: $[M+H]^+$ Calcld for $C_{19}H_{20}N_3O_3$: 338.1505; Found: 338.1526.

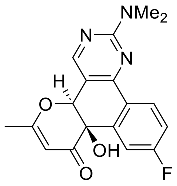

*2-(Dimethylamino)-10-fluoro-8a-hydroxy-6-methyl-4b,8a-dihydro-8H-benzo[h]pyrano[2,3-f]quinazolin-8-one (**11j**)*

Yellow powder; yield 56% (0.1 g); mp 156-158 °C. ^{1}H NMR (300 MHz, $CDCl_3$) δ 8.64 (dd, *J* = 10.9, 2.7 Hz, 1H), 8.39 (s, 1H), 8.32 (dd, *J* = 8.8, 6.0 Hz, 1H), 7.19 – 7.11 (m, 1H), 5.47 (s, 1H), 5.27 (s, 1H), 3.00 (s, 6H), 2.19 (s, 3H). ^{13}C NMR (75 MHz, $CDCl_3$) δ 190.67, 172.28, 164.34 (d, *J* = 251.2 Hz), 161.55, 156.64, 154.61, 138.63 (d, *J* = 9.4 Hz), 128.66, 128.28 (d, *J* = 8.9 Hz), 117.08 (d, *J* = 25.3 Hz), 116.51 (d, *J* = 21.8 Hz), 109.34, 104.80, 79.53, 66.14, 37.09, 20.70. HRMS (ESI-TOF) *m/z*: $[M+H]^+$ Calcld for $C_{18}H_{17}FN_3O_3$: 342.1253; Found: 342.1241.

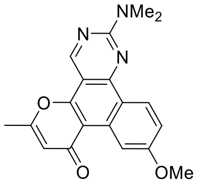

*2-(Dimethylamino)-10-methoxy-6-methyl-8H-benzo[h]pyrano[2,3-f]quinazolin-8-one (**12a**)*

Pale yellow powder; yield 66% (0.11 g); mp 221-223 °C. ^{1}H NMR (300 MHz, $CDCl_3$) δ 9.51 (d, *J* = 2.6 Hz, 1H), 9.20 (s, 1H), 8.79 (d, *J* = 9.0 Hz, 1H), 7.16 (dd, *J* = 9.0, 2.6 Hz, 1H), 6.23 (s, 1H), 4.01 (s, 3H), 3.37 (s, 6H), 2.42 (s, 3H). ^{13}C NMR (151 MHz, $CDCl_3$) δ 179.21, 162.47, 162.29, 155.82, 155.21, 154.61, 134.49, 126.09, 121.03, 116.87, 114.22, 111.40, 108.24, 105.83, 55.69, 37.50, 19.72. HRMS (ESI-TOF) *m/z*: $[M+H]^+$ Calcld for $C_{19}H_{18}N_3O_3$: 336.1348; Found: 336.1348.

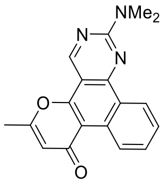

*2-(Dimethylamino)-6-methyl-8H-benzo[h]pyrano[2,3-f]quinazolin-8-one (**12b**)*

White powder; yield 61% (0.09 g); mp 259-261 °C. ^{1}H NMR (300 MHz, $CDCl_3$) δ 9.96 (d, *J* = 8.5 Hz, 1H), 9.32 (s, 1H), 9.00 (dd, *J* = 8.1, 1.6 Hz, 1H), 7.83 – 7.74 (m, 1H), 7.67 – 7.58 (m, 1H), 6.29 (s, 1H), 3.42 (s, 6H), 2.45 (s, 3H). ^{13}C NMR (75 MHz, $CDCl_3$) δ 179.09, 162.25, 161.11, 155.63, 155.36, 155.15, 132.53, 131.59, 127.56, 127.34, 126.75, 124.51, 114.43, 111.93, 106.92, 37.52, 19.70. HRMS (ESI-TOF) *m/z*: $[M+H]^+$ Calcld for $C_{18}H_{16}N_3O_2$: 306.1242; Found: 306.1259.

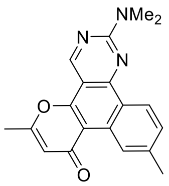

*2-(Dimethylamino)-6,10-dimethyl-8H-benzo[h]pyrano[2,3-f]quinazolin-8-one (**12c**)*

Pale yellow powder; yield 68% (0.11 g); mp 252-254 °C. ^{1}H NMR (300 MHz, $CDCl_3$) δ 9.79 (s, 1H), 9.35 (s, 1H), 8.91 (d, *J* = 8.3 Hz, 1H), 7.45 (dd, *J* = 8.3, 1.8 Hz, 1H), 6.30 (s, 1H), 3.42 (s, 6H), 2.61 (s, 3H), 2.45 (s, 3H). ^{13}C NMR (126 MHz, $CDCl_3$) δ 179.30, 162.13, 161.50, 155.91, 155.67, 155.01, 142.03, 132.56, 128.29, 127.32, 125.23, 124.39, 123.75, 114.39, 111.74, 106.63, 37.44, 22.57, 19.69. HRMS (ESI-TOF) *m/z*: $[M+H]^+$ Calcld for $C_{19}H_{18}N_3O_2$: 320.1399; Found: 320.1381.

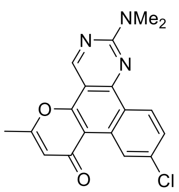

*10-Chloro-2-(dimethylamino)-6-methyl-8H-benzo[h]pyrano[2,3-f]quinazolin-8-one (**12d**)*

White powder; yield 44% (0.07 g); mp > 300 °C. ^{1}H NMR (300 MHz, $CDCl_3$) δ 10.04 (s, 1H), 9.37 (s, 1H), 8.93 (d, *J* = 8.8 Hz, 1H), 7.57 (d, *J* = 8.8 Hz, 1H), 6.31 (s, 1H), 3.42 (s, 6H), 2.47 (s, 3H). ^{13}C NMR (126 MHz, $CDCl_3$) δ 178.78, 162.46, 161.56, 156.16, 156.03,

154.59, 138.02, 133.43, 127.29, 126.94, 125.90, 125.80, 114.39, 110.93, 106.81, 37.48, 19.75. HRMS (ESI-TOF) *m/z*: $[M+H]^+$ Calcld for $C_{18}H_{15}ClN_3O_2$: 340.0852; Found: 340.0839.

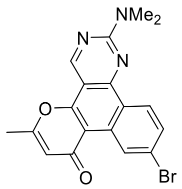

*10-Bromo-2-(dimethylamino)-6-methyl-8H-benzo[h]pyrano[2,3-f]quinazolin-8-one (**12e**)*

White powder; yield 41% (0.08 g); mp > 300 °C. ^{1}H NMR (300 MHz, $CDCl_3$) δ 10.21 (d, *J* = 2.1 Hz, 1H), 9.37 (s, 1H), 8.86 (d, *J* = 8.7 Hz, 1H), 7.72 (dd, *J* = 8.7, 2.1 Hz, 1H), 6.30 (s, 1H), 3.42 (s, 6H), 2.47 (s, 3H). ^{13}C NMR (126 MHz, $CDCl_3$) δ 178.71, 162.49, 161.40, 156.05, 155.91, 154.75, 133.66, 130.10, 130.00, 126.85, 126.10, 126.00, 114.41, 110.86, 106.84, 37.52, 19.76. HRMS (ESI-TOF) *m/z*: $[M+H]^+$ Calcld for $C_{18}H_{15}BrN_3O_2$: 384.0347; Found: 384.0342.

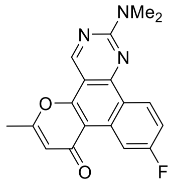

*2-(Dimethylamino)-10-fluoro-6-methyl-8H-benzo[h]pyrano[2,3-f]quinazolin-8-one (**12f**)*

White powder; yield 54% (0.09 g); mp 255-257 °C. ^{1}H NMR (300 MHz, DMSO-d_6) δ 9.54 (d, *J* = 13.0 Hz, 1H), 9.25 (s, 1H), 8.91 – 8.81 (m, 1H), 7.55 – 7.40 (m, 1H), 6.31 (s, 1H), 3.33 (s, 6H), 2.45 (s, 3H). ^{13}C NMR (126 MHz, DMSO-d_6) δ 177.23, 163.24 (d, *J* = 245.9 Hz), 162.88, 160.62, 156.29, 155.79, 155.16, 153.04, 133.24 (d, *J* = 12.6 Hz), 126.53 (d, *J* = 9.9 Hz), 123.09, 114.52 (d, *J* = 23.6 Hz), 112.94, 111.21 (d, *J* = 26.0 Hz), 105.55, 36.48, 18.48. HRMS (ESI-TOF) *m/z*: $[M+H]^+$ Calcld for $C_{18}H_{15}FN_3O_2$: 324.1148; Found: 324.1163.

4. NMR ^{1}H and ^{13}C spectra for starting compounds 9 and 10

^{1}H NMR spectrum (300 MHz) of **9a** in DMSO-d_6

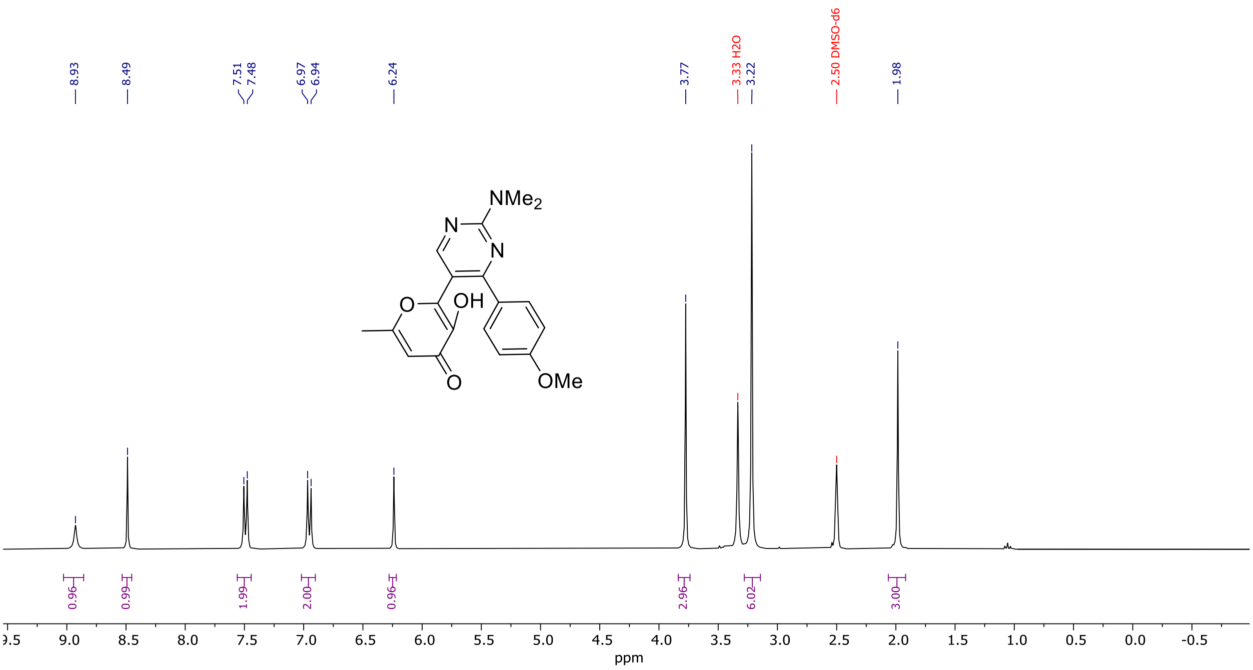

^{13}C {^{1}H} NMR spectrum (75 MHz) of **9a** in DMSO-d_6

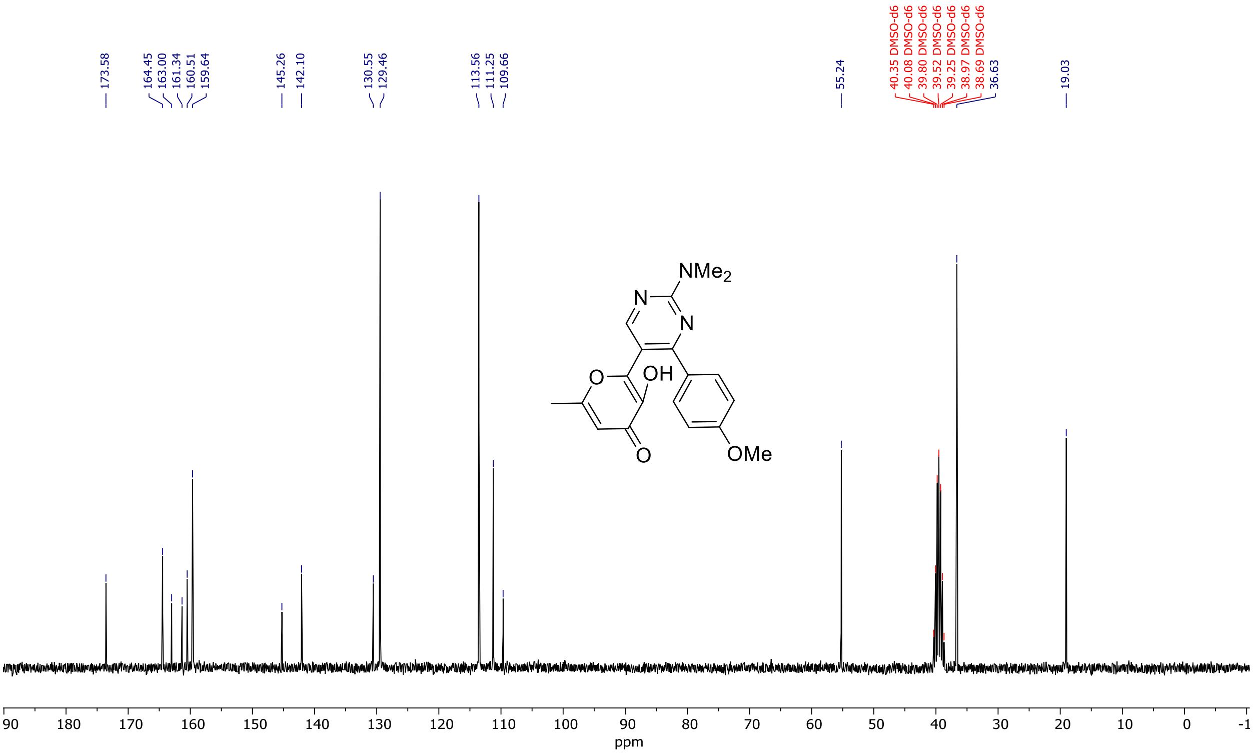

^{1}H NMR spectrum (300 MHz) of **9b** in DMSO-d_6

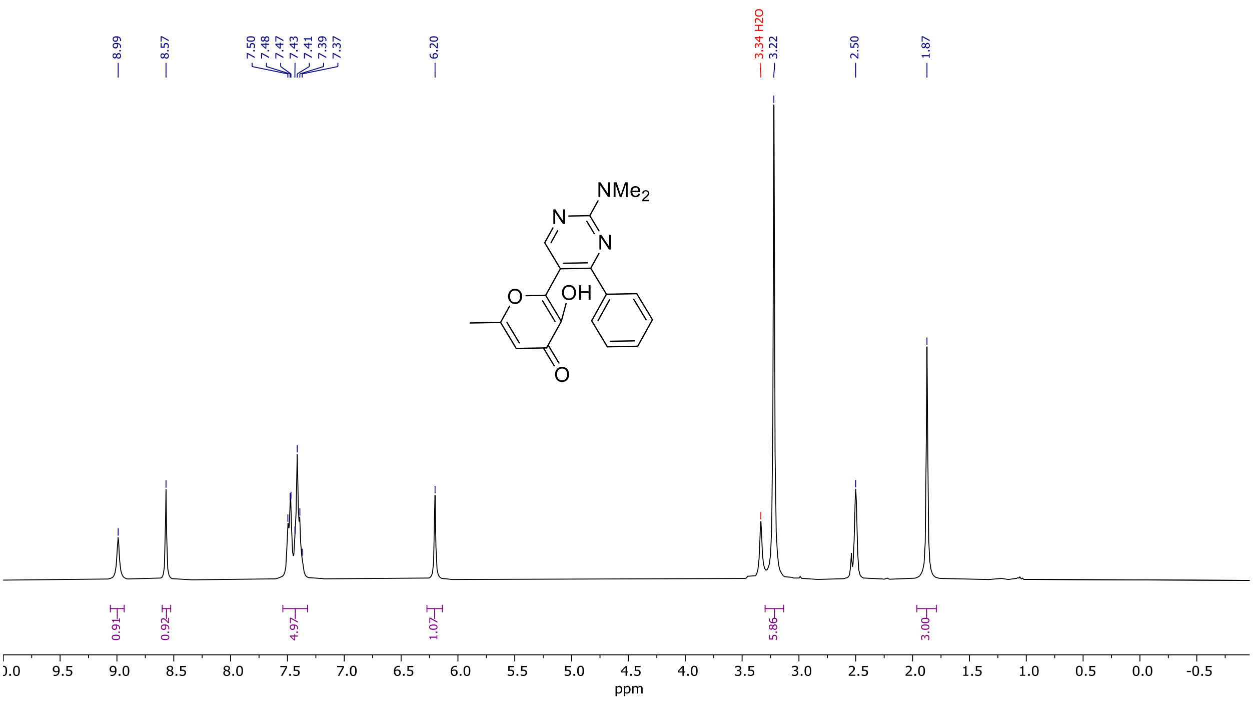

^{13}C {^{1}H} NMR spectrum (75 MHz) of **9b** in DMSO-d_6

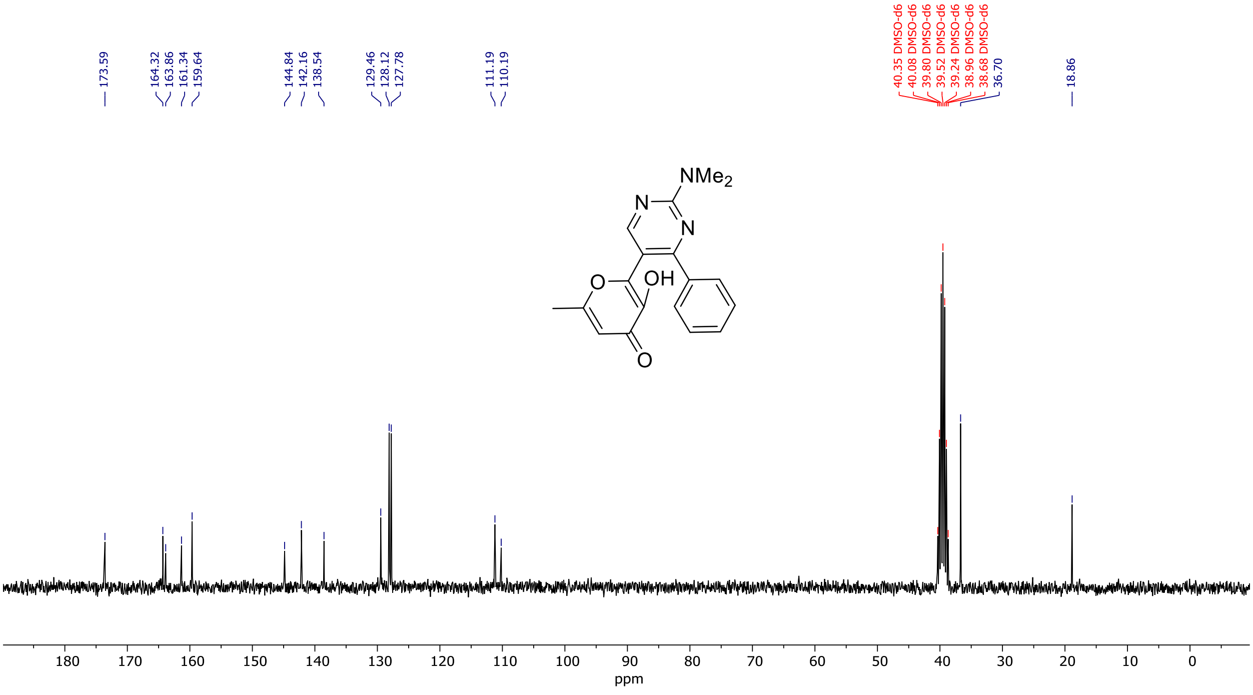

^{1}H NMR spectrum (300 MHz) of **9c** in DMSO-d_6

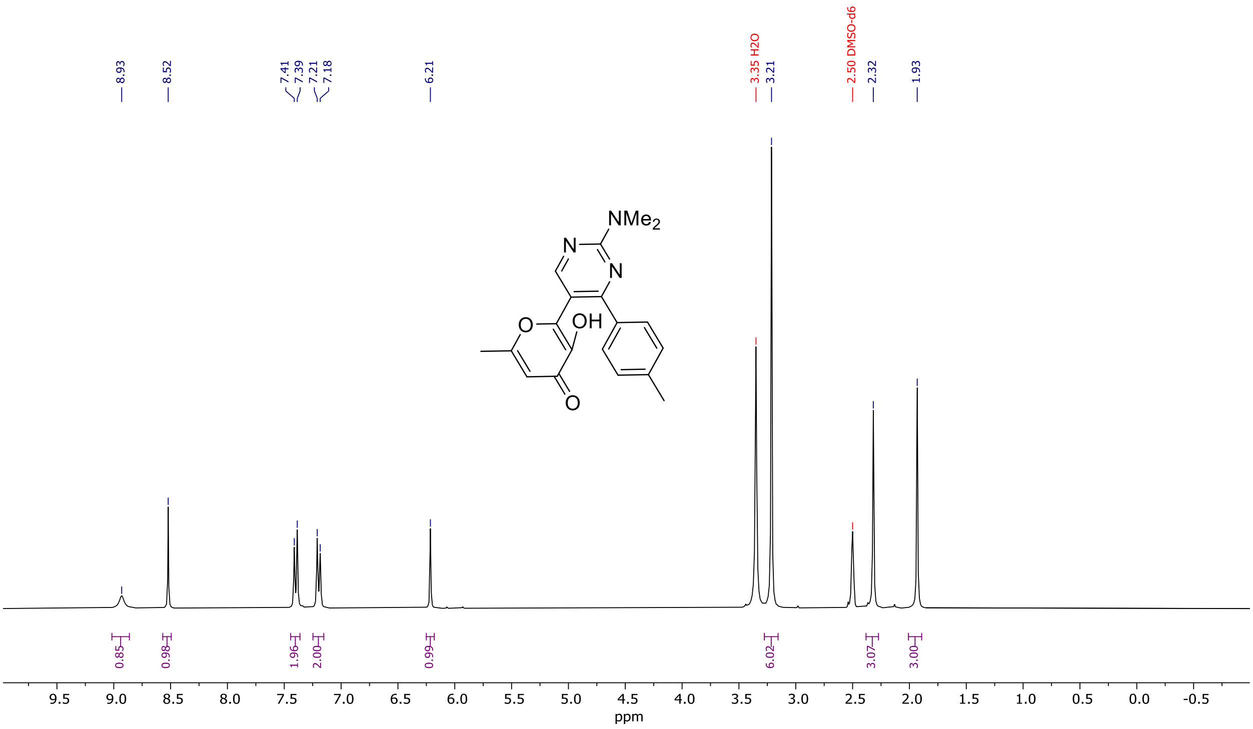

^{13}C {^{1}H} NMR spectrum (75 MHz) of **9c** in DMSO-d_6

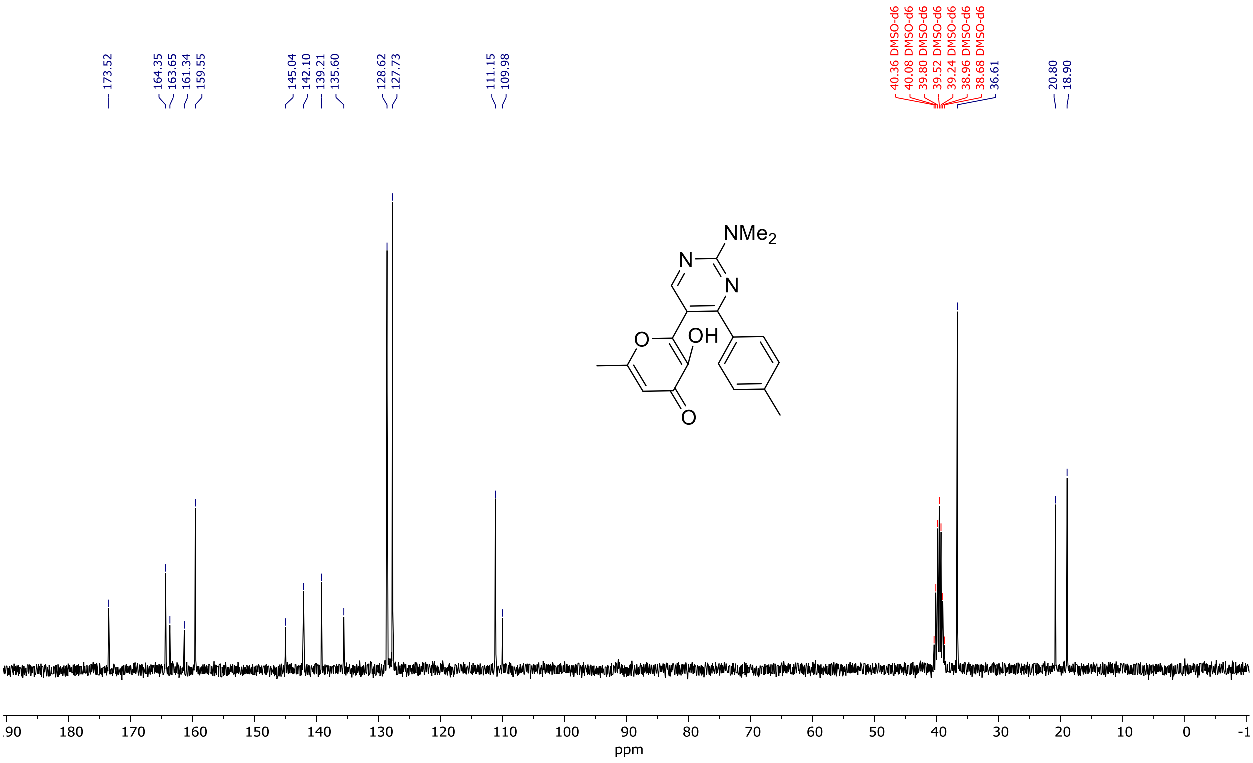

^{1}H NMR spectrum (300 MHz) of **9d** in DMSO-d_6

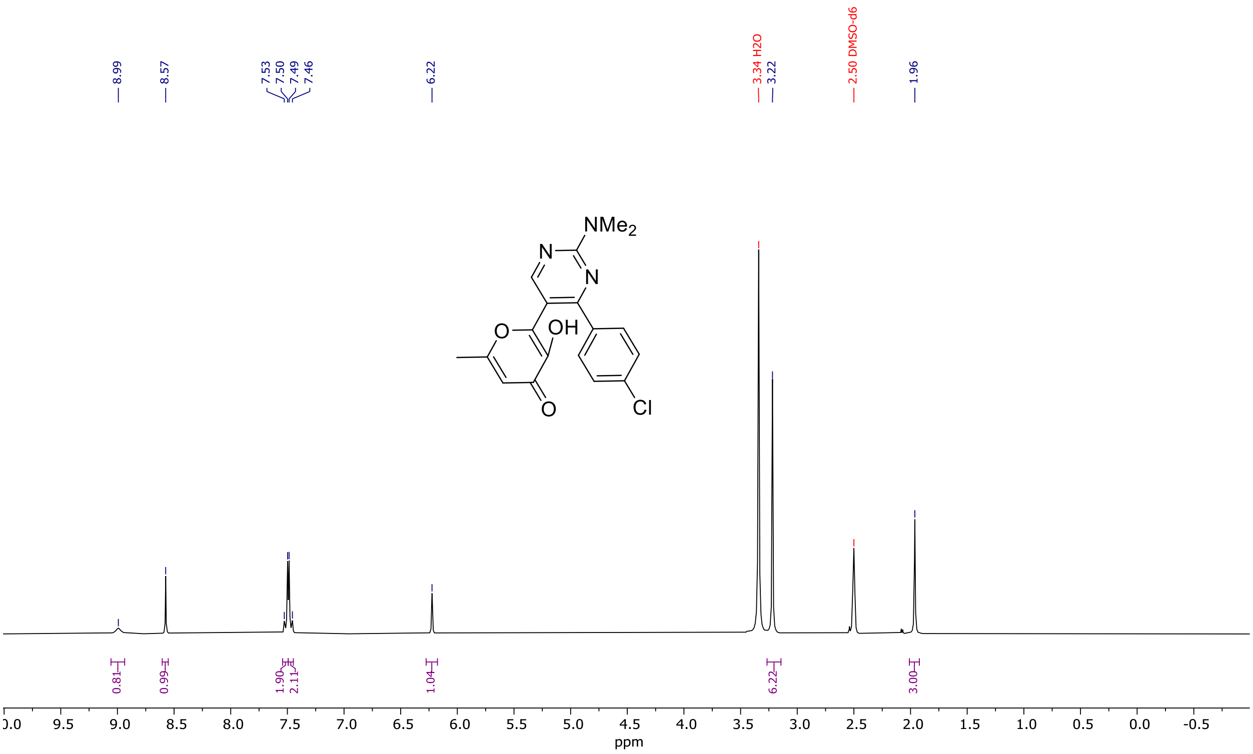

^{13}C {^{1}H} NMR spectrum (75 MHz) of **9d** in DMSO-d_6

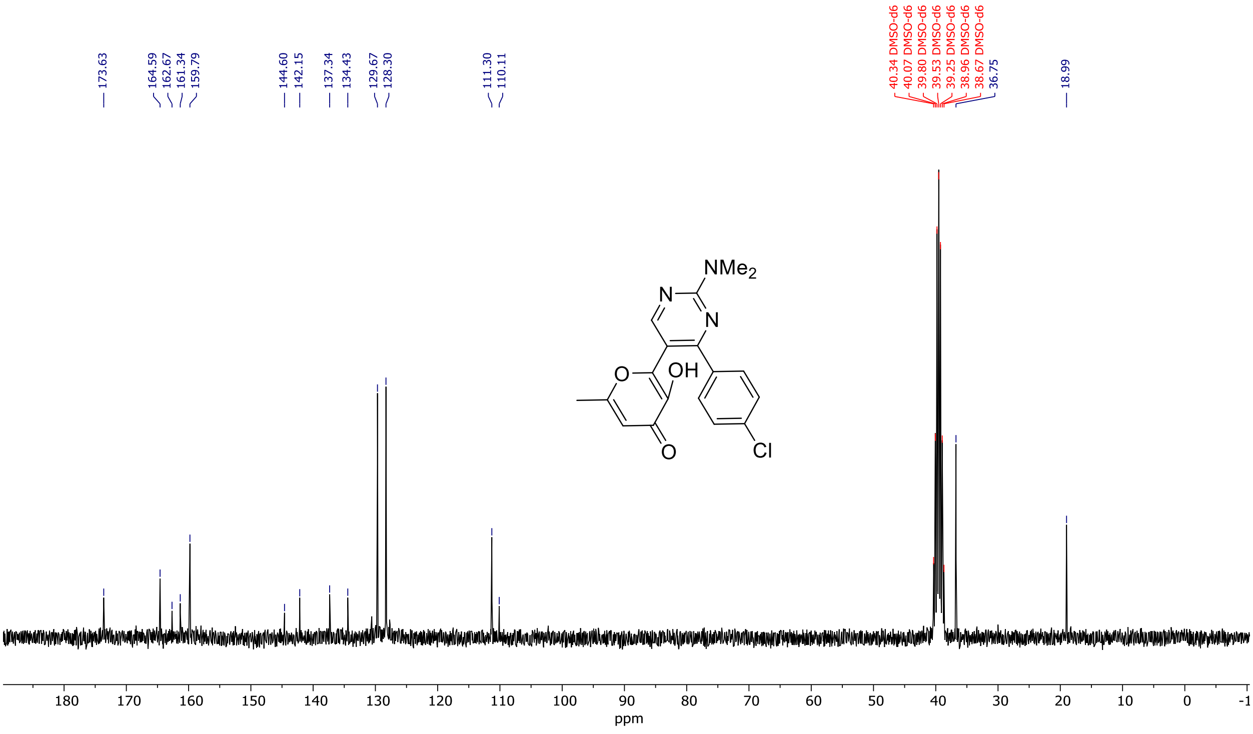

^{1}H NMR spectrum (300 MHz) of **9e** in DMSO-d_6

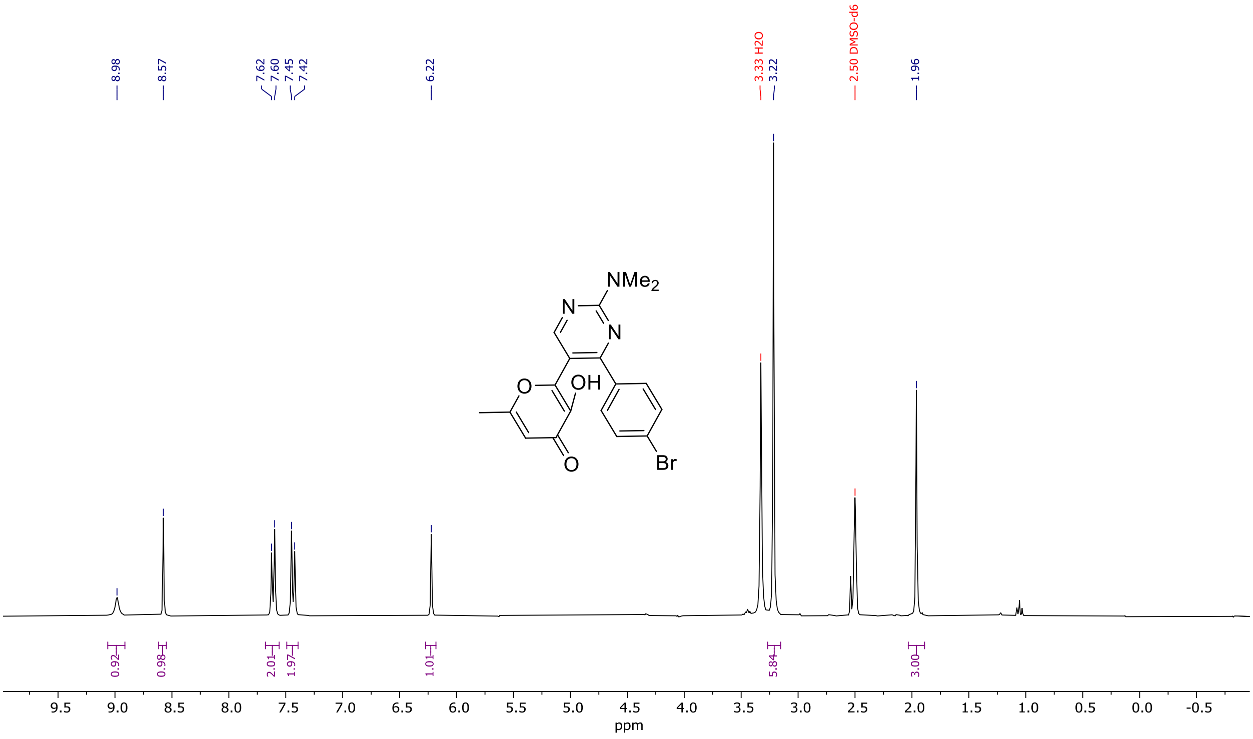

^{13}C {^{1}H} NMR spectrum (75 MHz) of **9e** in DMSO-d_6

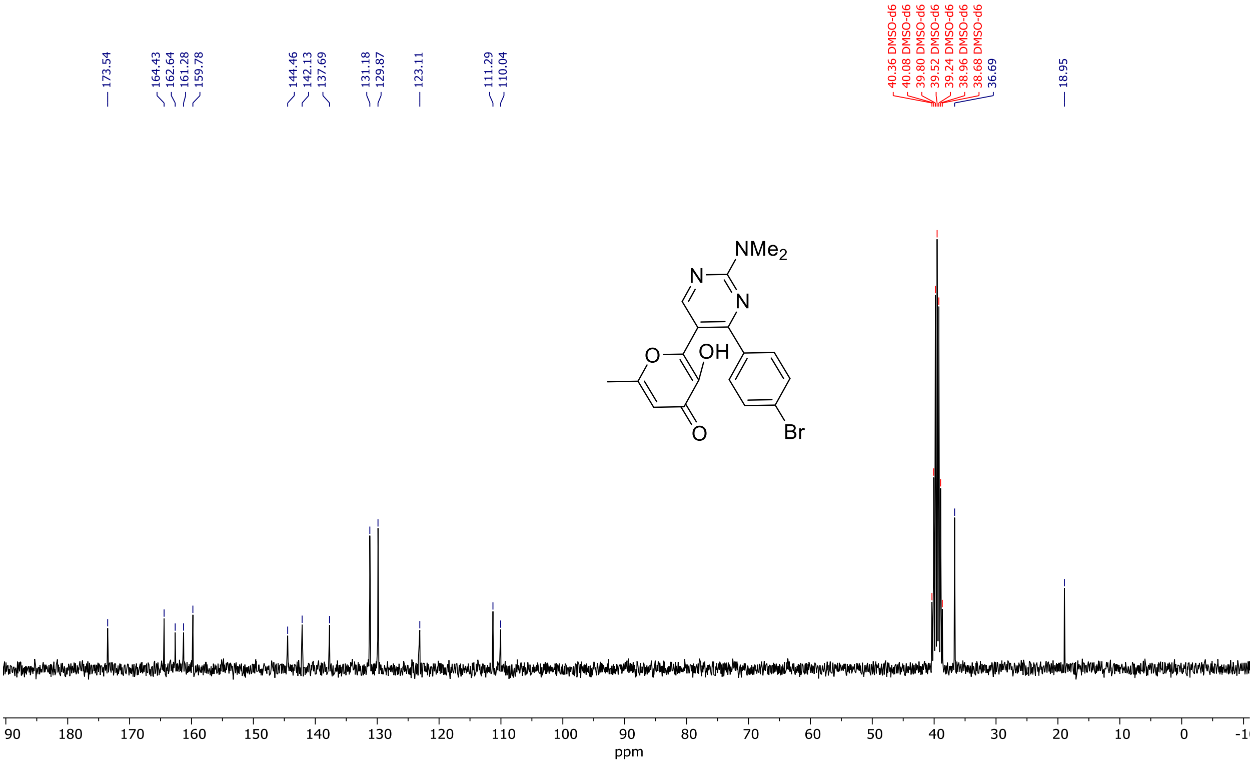

^{1}H NMR spectrum (300 MHz) of **9f** in DMSO-d_6

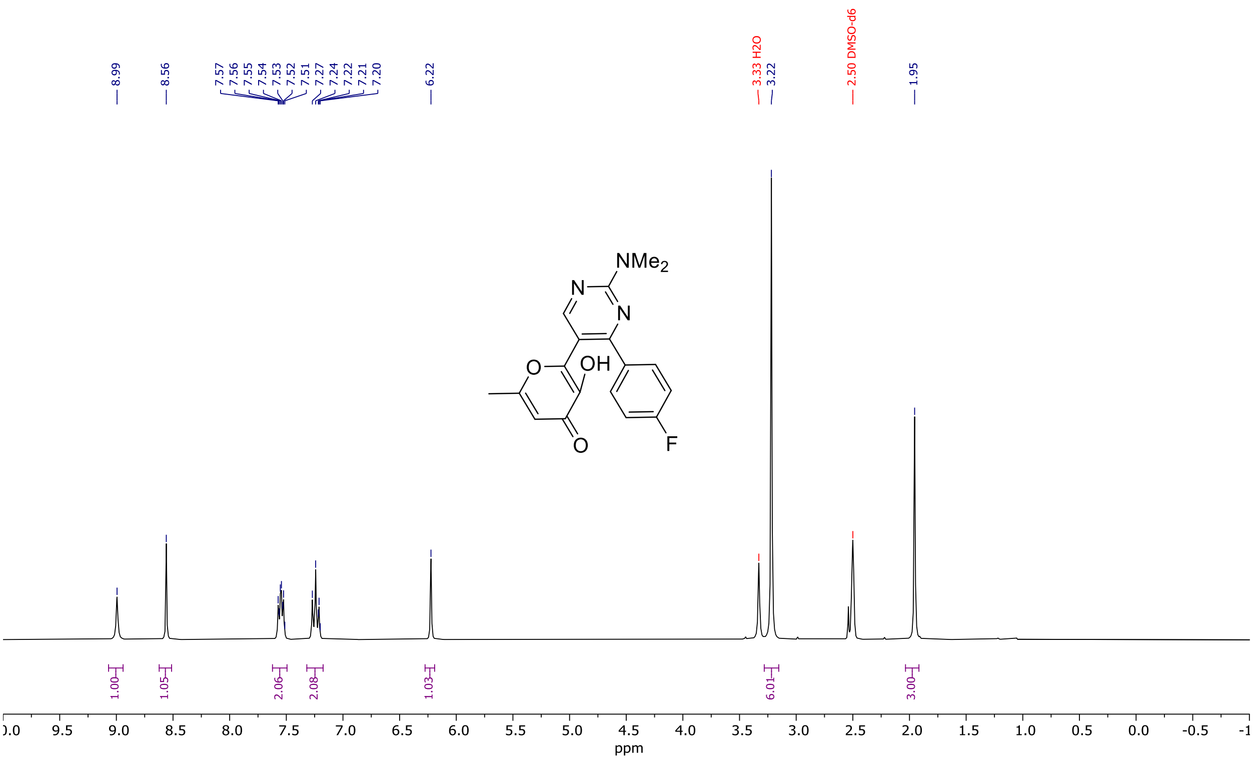

^{13}C {^{1}H} NMR spectrum (75 MHz) of **9f** in DMSO-d_6

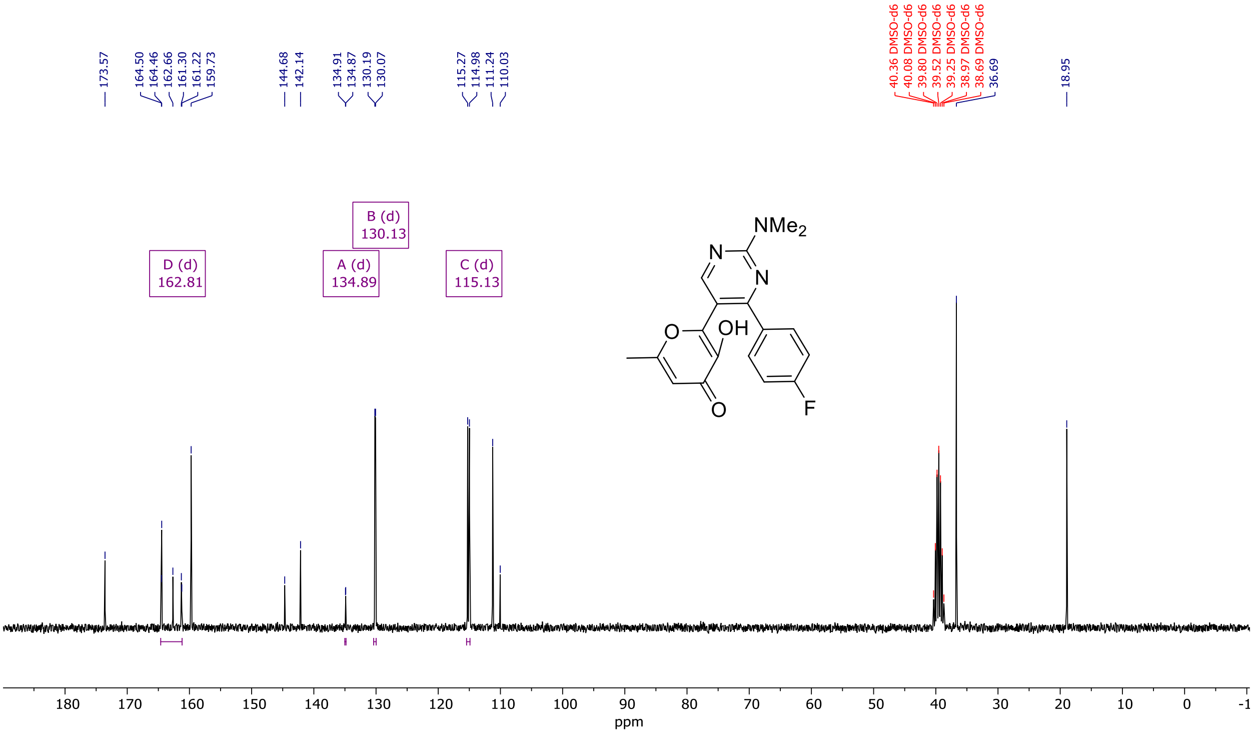

^{1}H NMR spectrum (300 MHz) of **9g** in DMSO-d_6

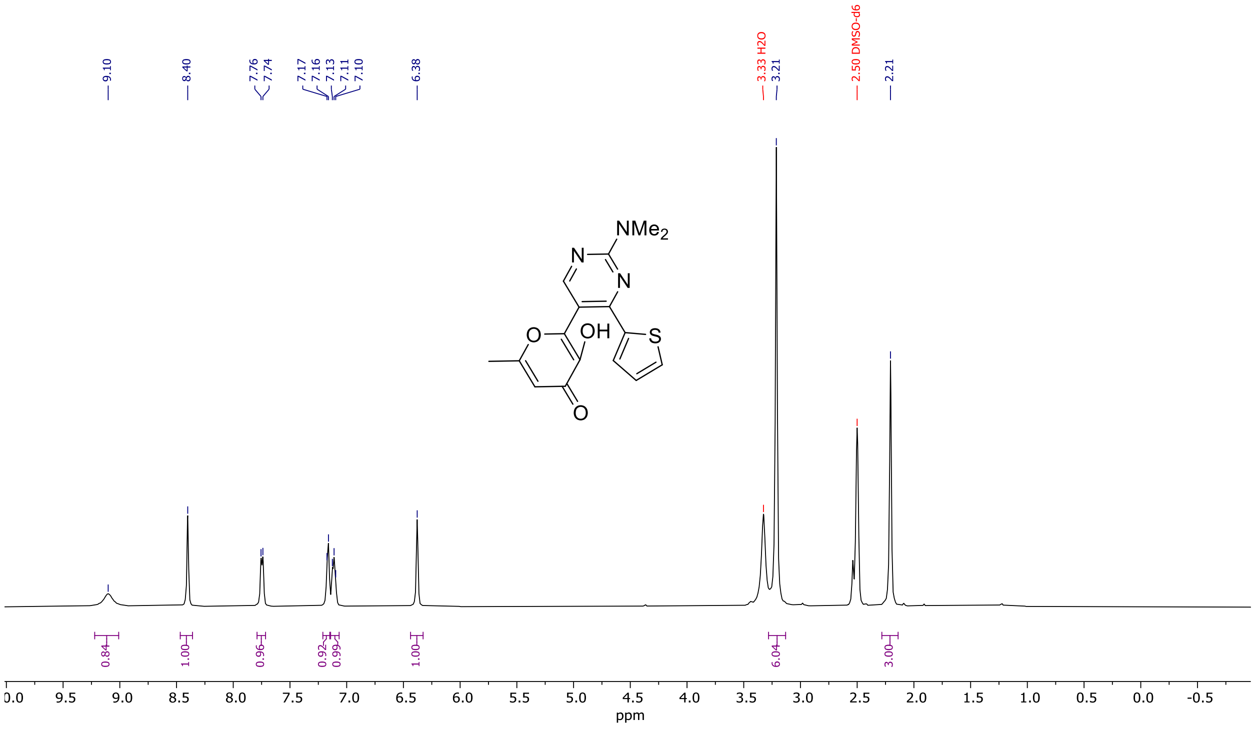

^{13}C {^{1}H} NMR spectrum (75 MHz) of **9g** in DMSO-d_6

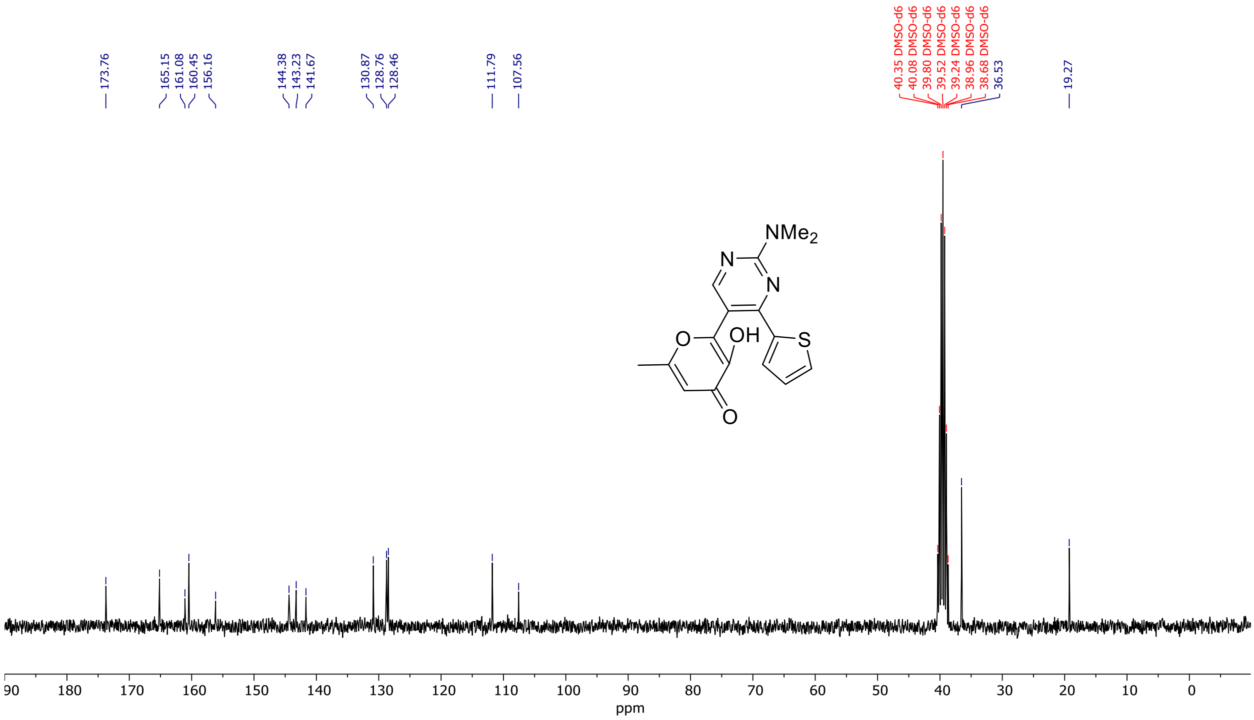

^{1}H NMR spectrum (300 MHz) of **10a** in DMSO-d_6

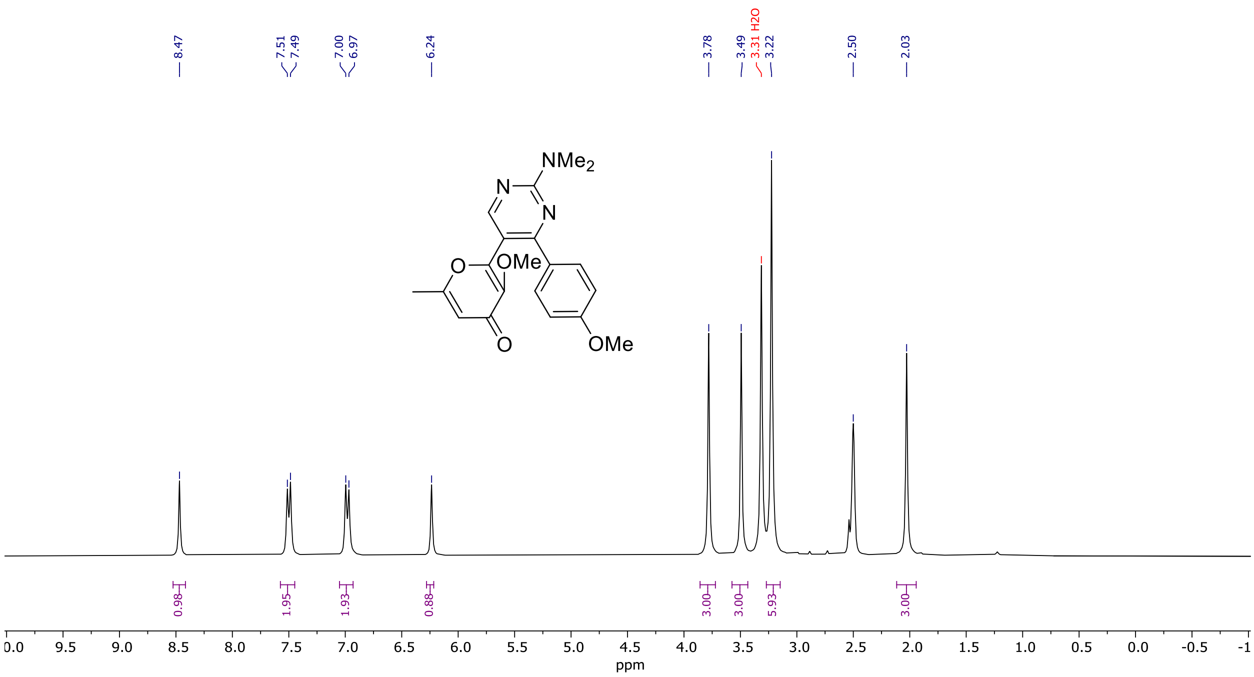

^{13}C {^{1}H} NMR spectrum (126 MHz) of **10a** in DMSO-d_6

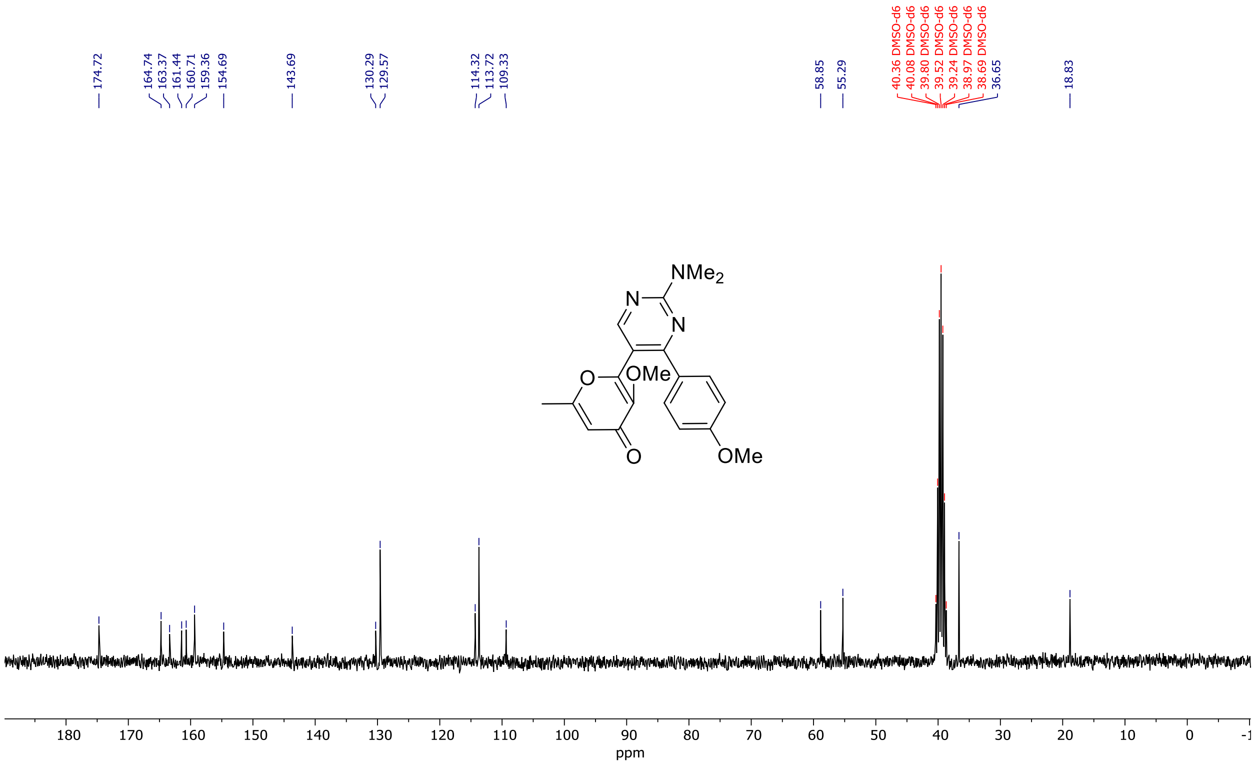

^{1}H NMR spectrum (300 MHz) of **10b** in DMSO-d_6

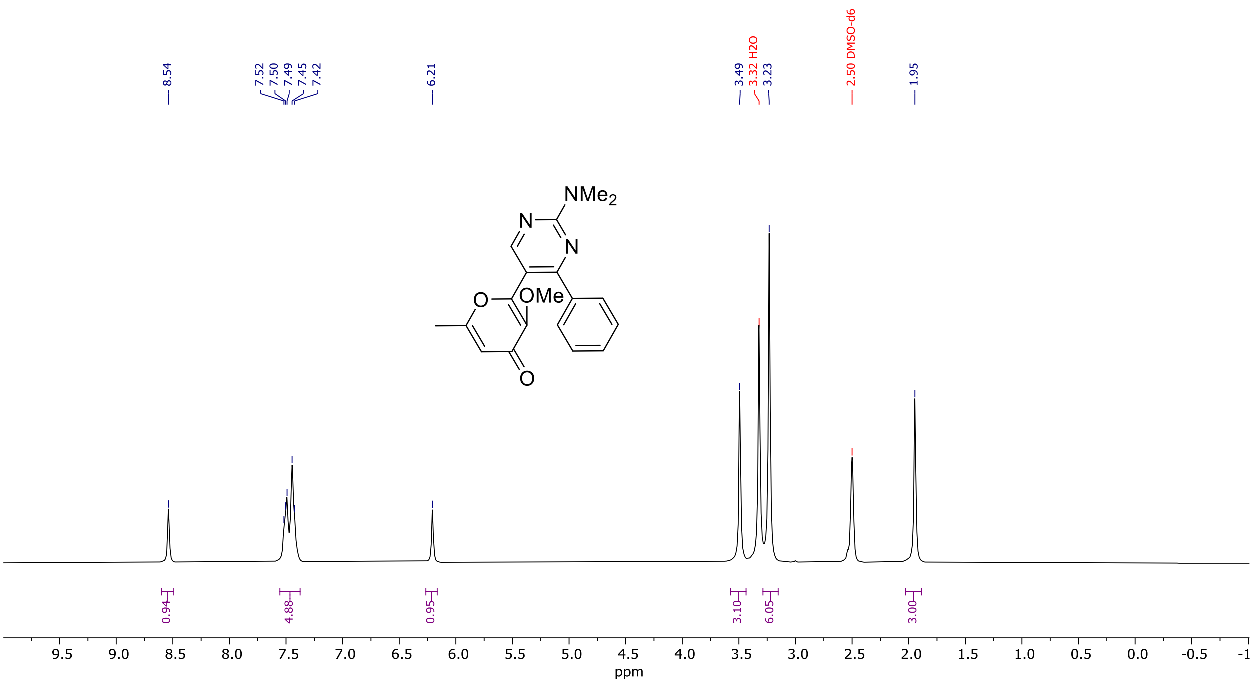

^{13}C {^{1}H} NMR spectrum (126 MHz) of **10b** in DMSO-d_6

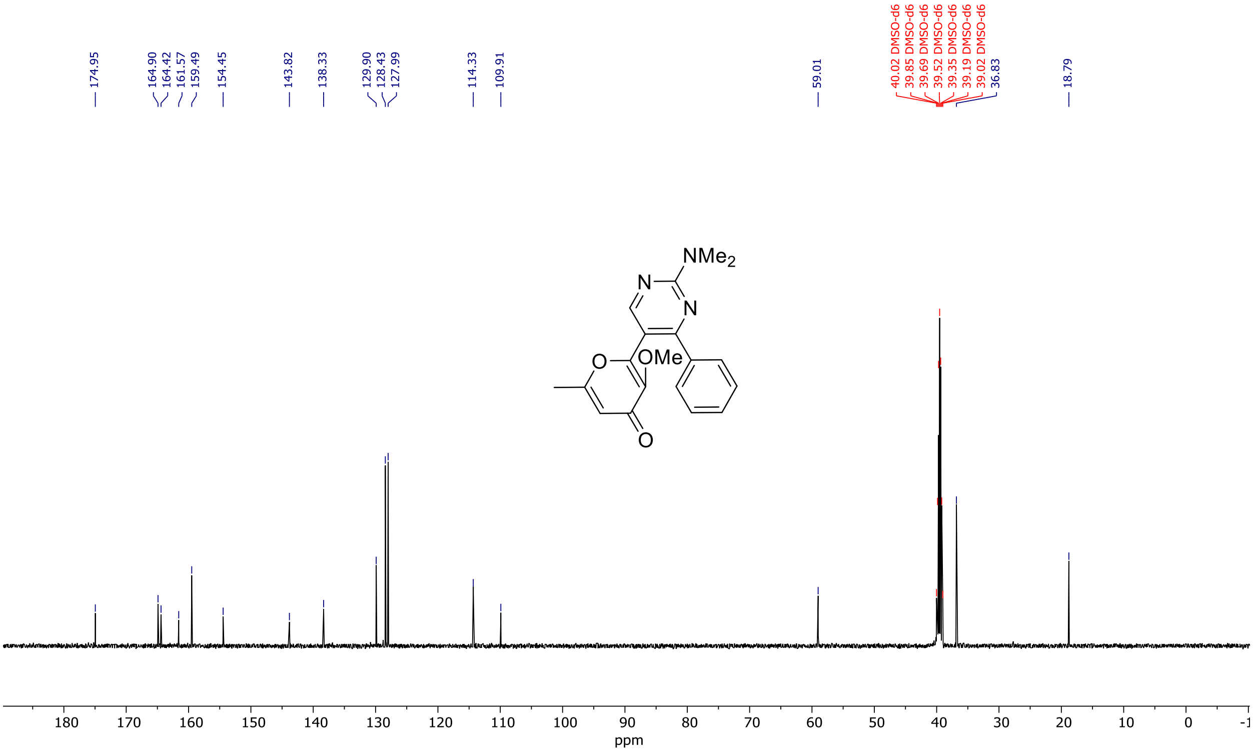

^{1}H NMR spectrum (300 MHz) of **10c** in DMSO-d_6

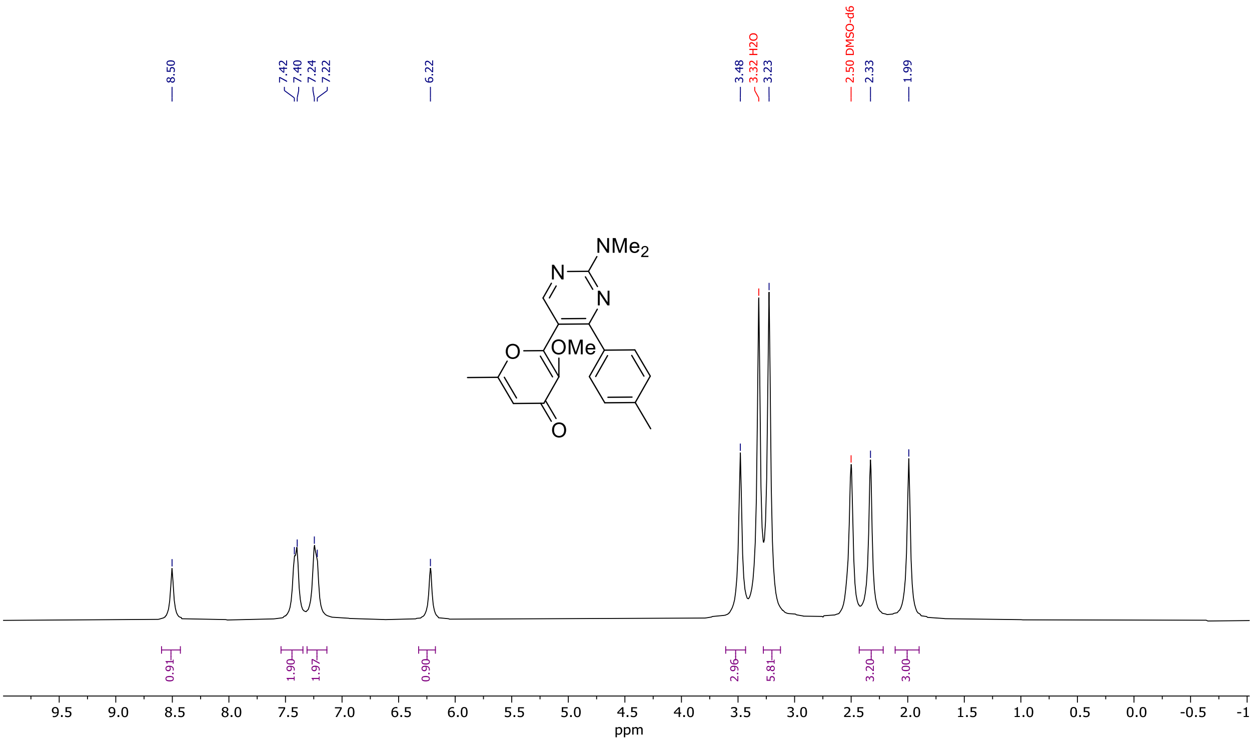

^{13}C {^{1}H} NMR spectrum (75 MHz) of **10c** in DMSO-d_6

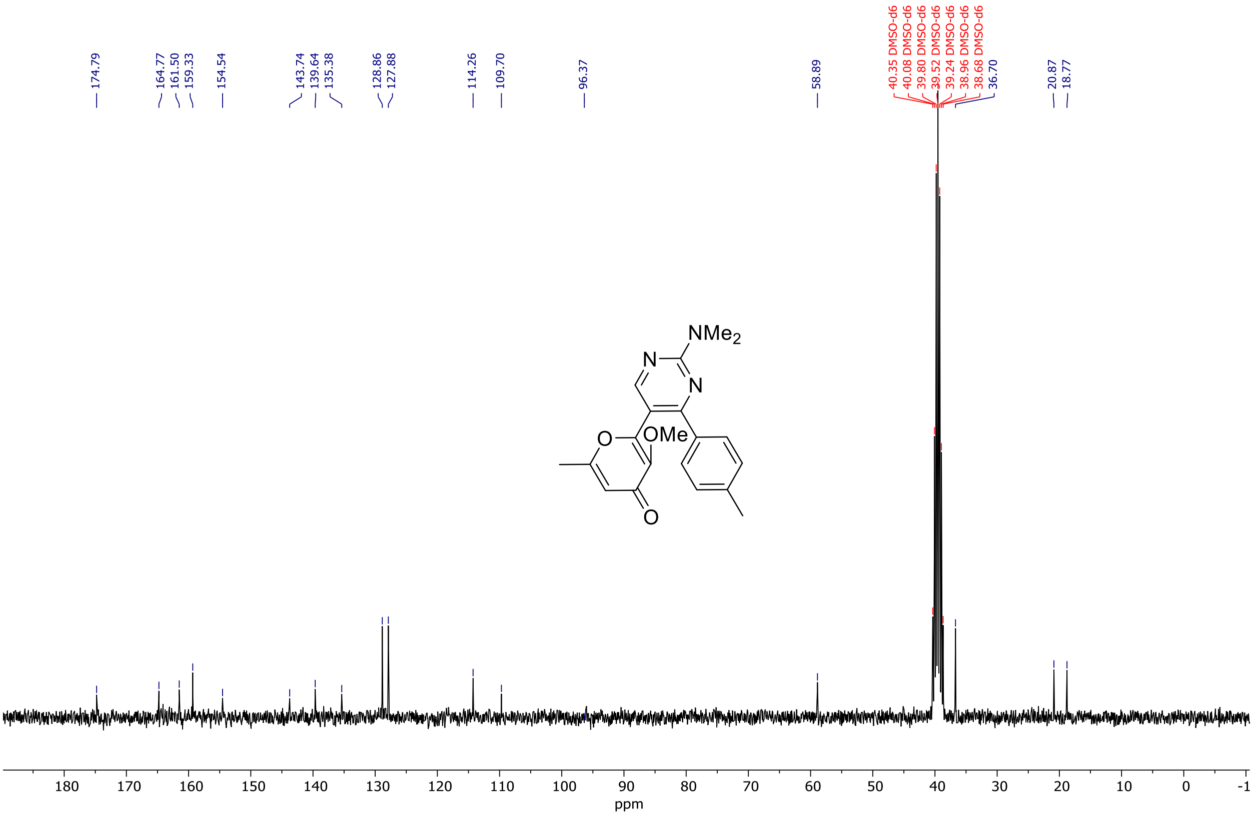

^{1}H NMR spectrum (300 MHz) of **10d** in DMSO-d_6

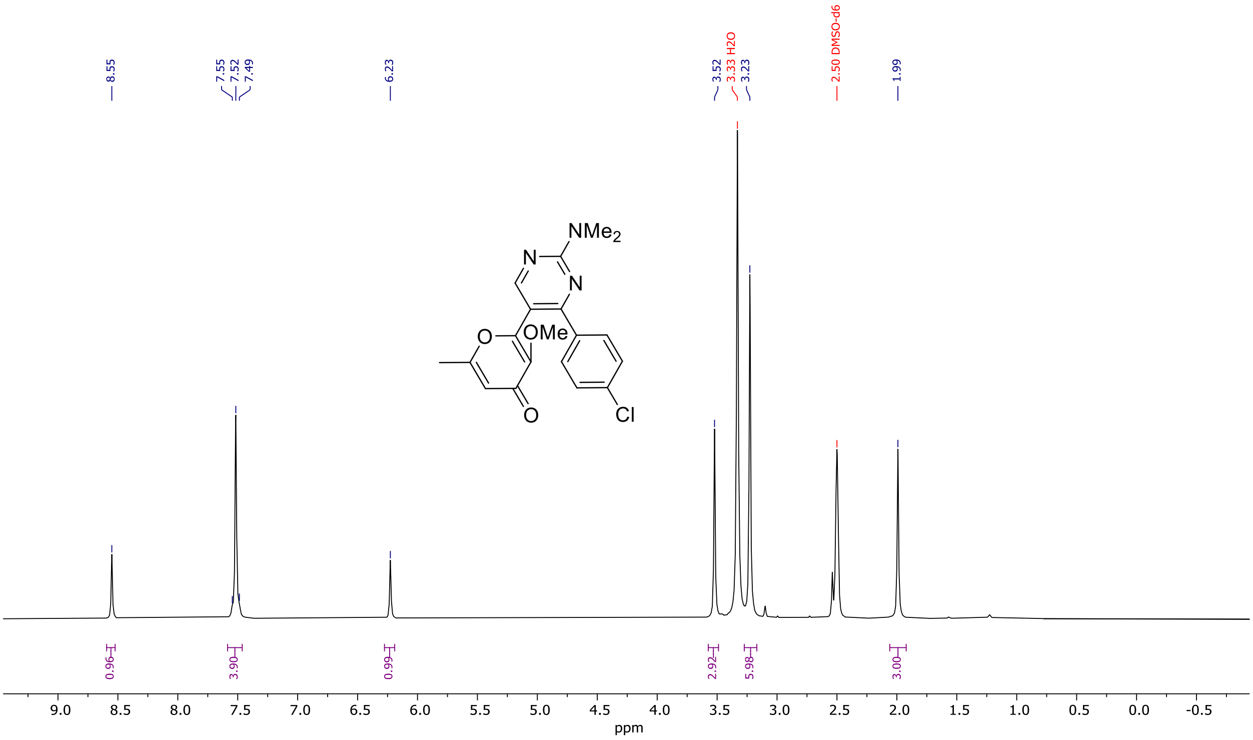

$^{13}C\ \{^{1}H\}$ NMR spectrum (75 MHz) of **10d** in DMSO-d_6

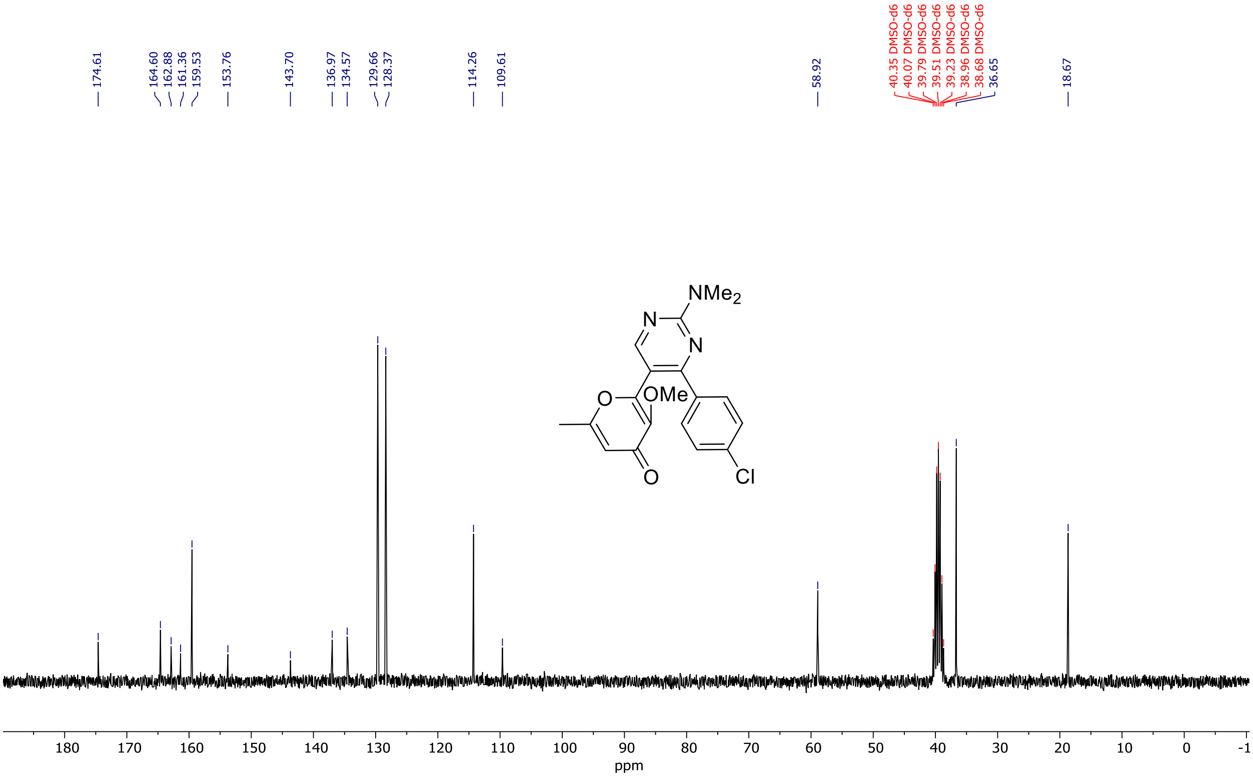

^{1}H NMR spectrum (300 MHz) of **10e** in DMSO-d_6

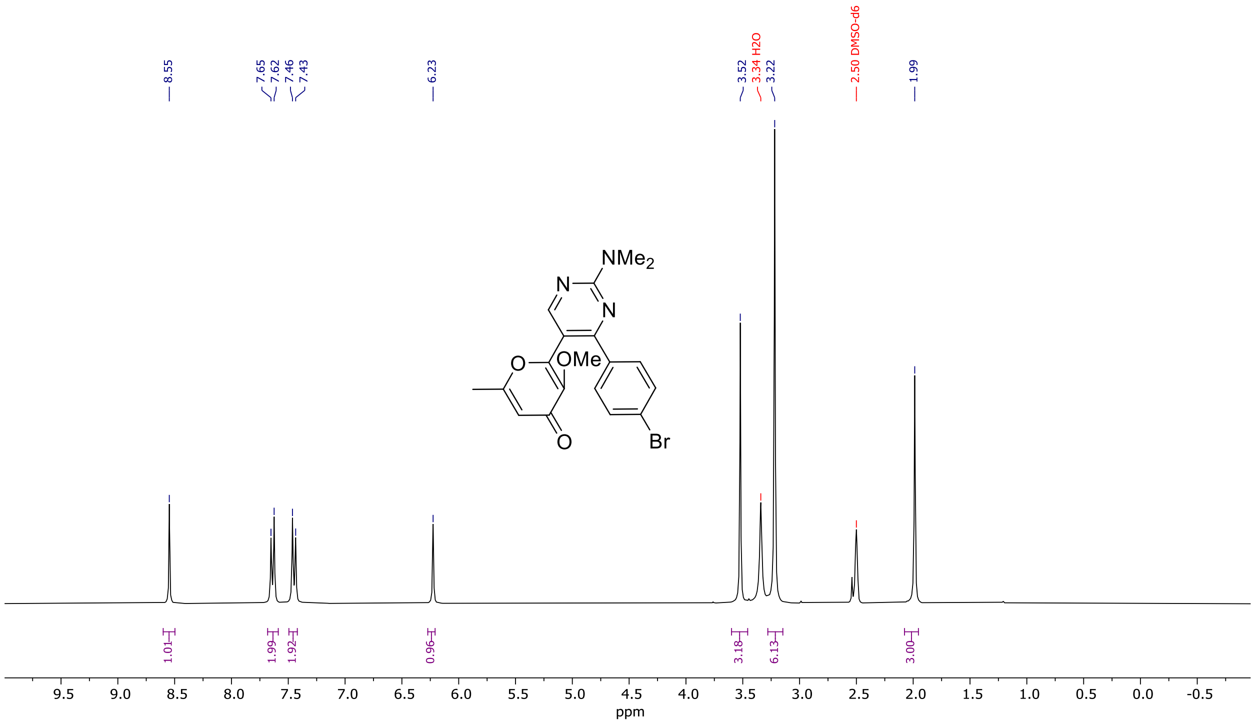

$^{13}C\{^{1}H\}$ NMR spectrum (75 MHz) of **10e** in DMSO-d_6

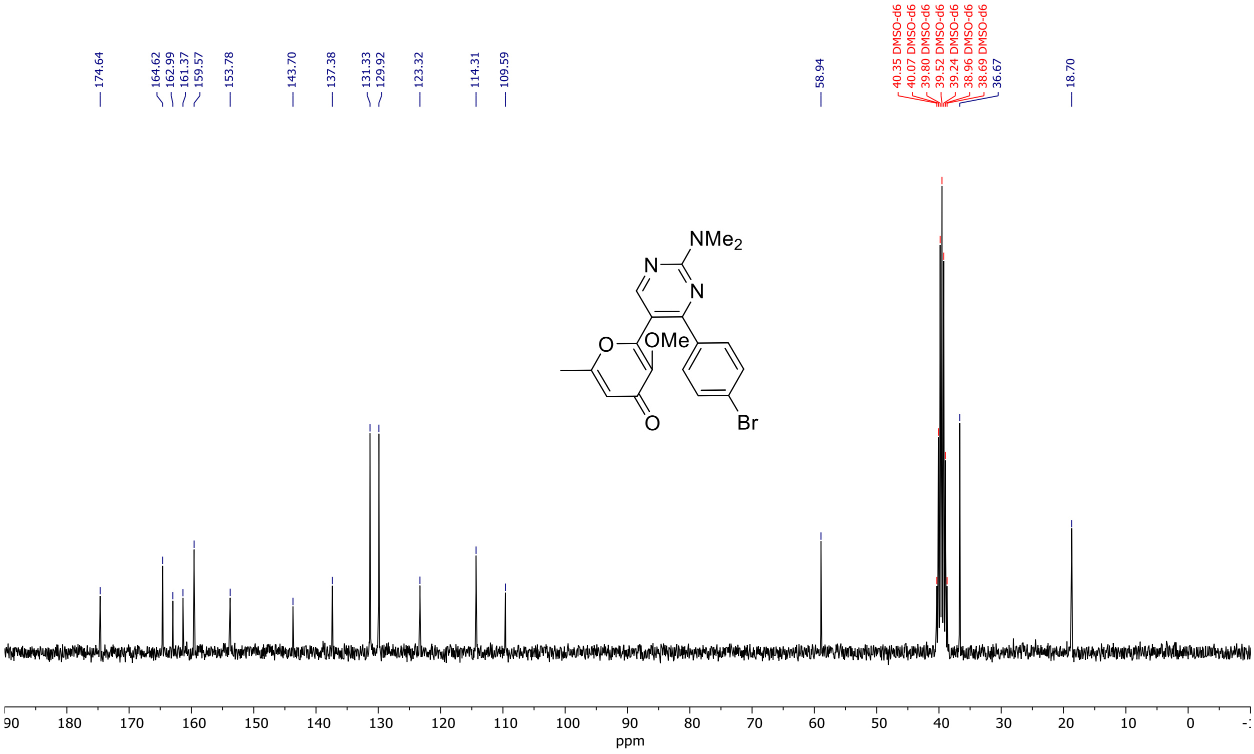

^{1}H NMR spectrum (300 MHz) of **10f** in DMSO-d_6

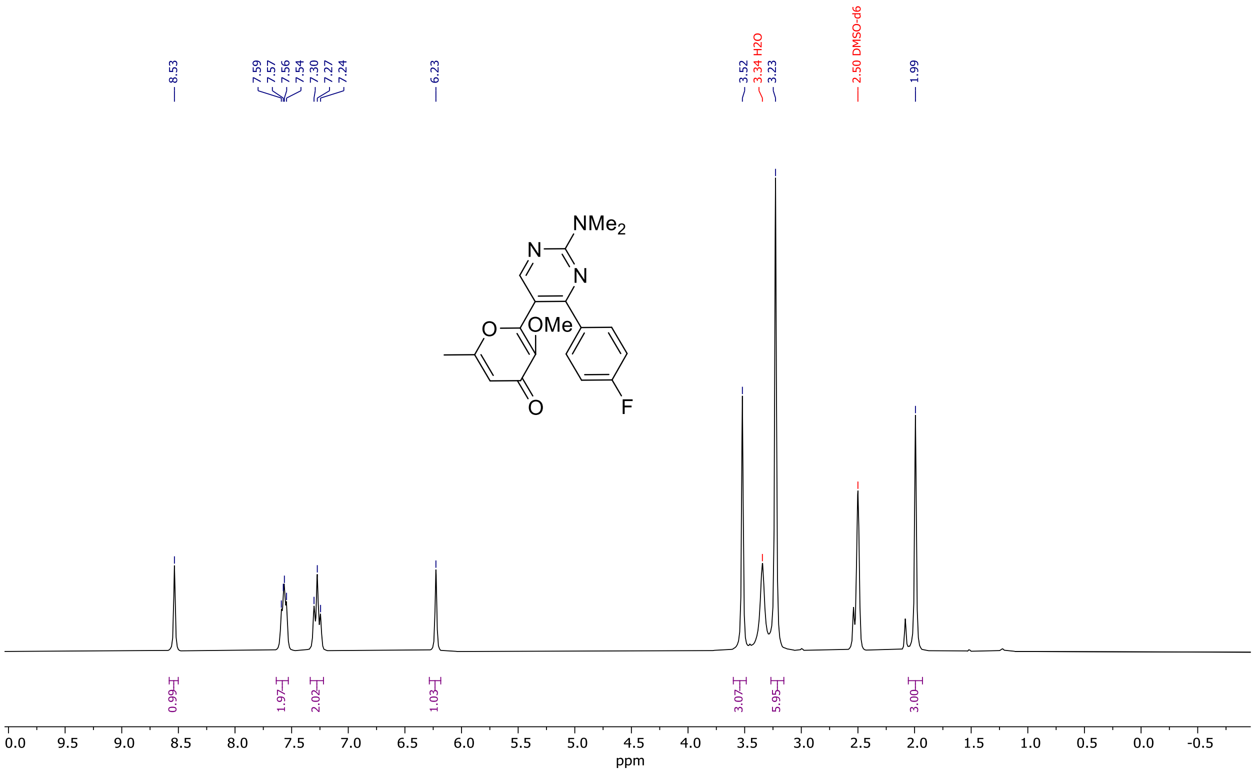

^{13}C {^{1}H} NMR spectrum (75 MHz) of **10f** in DMSO-d_6

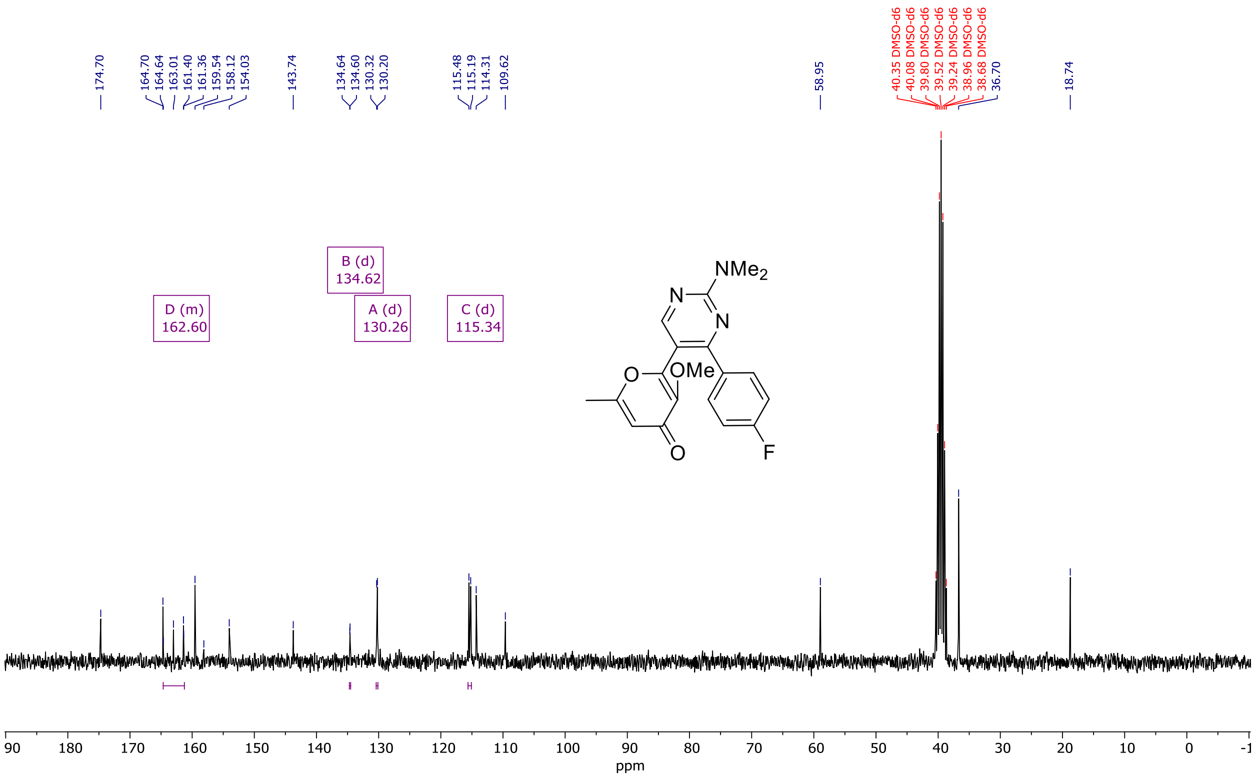

^{1}H NMR spectrum (300 MHz) of **10g** in DMSO-d_6

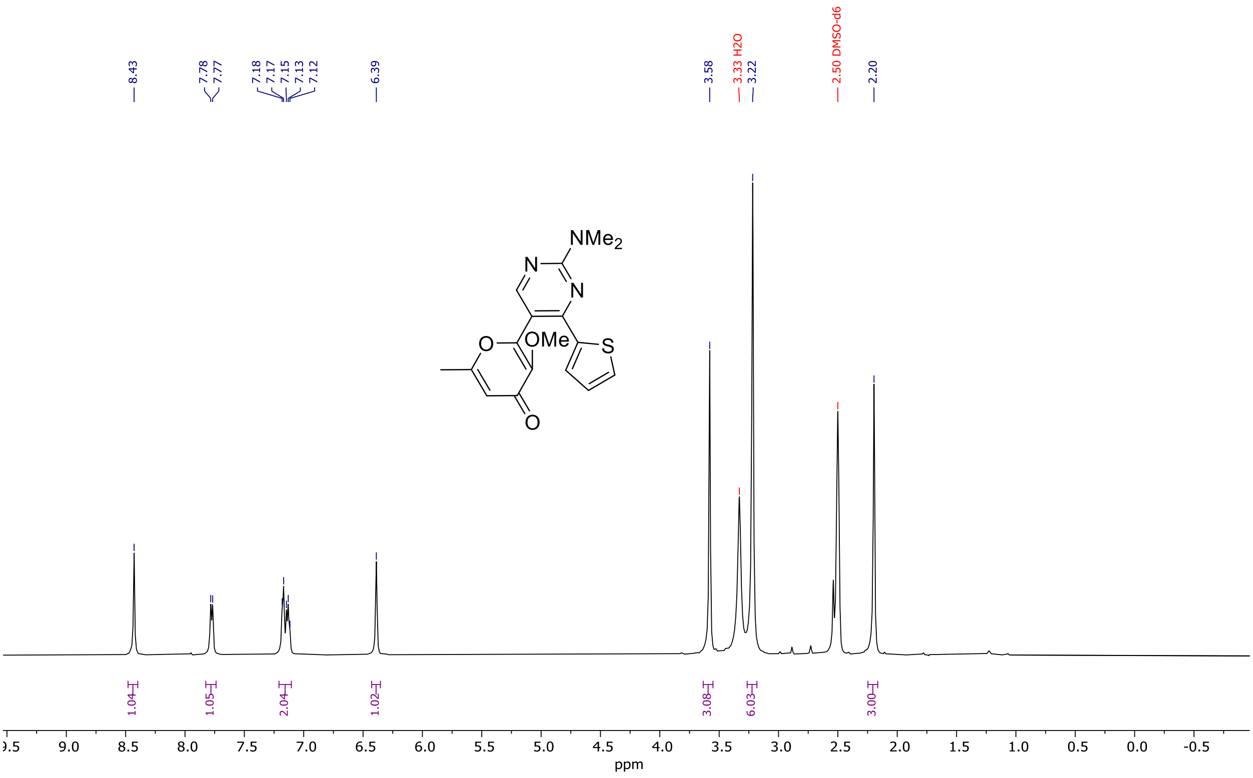

^{13}C {^{1}H} NMR spectrum (75 MHz) of **10g** in DMSO-d_6

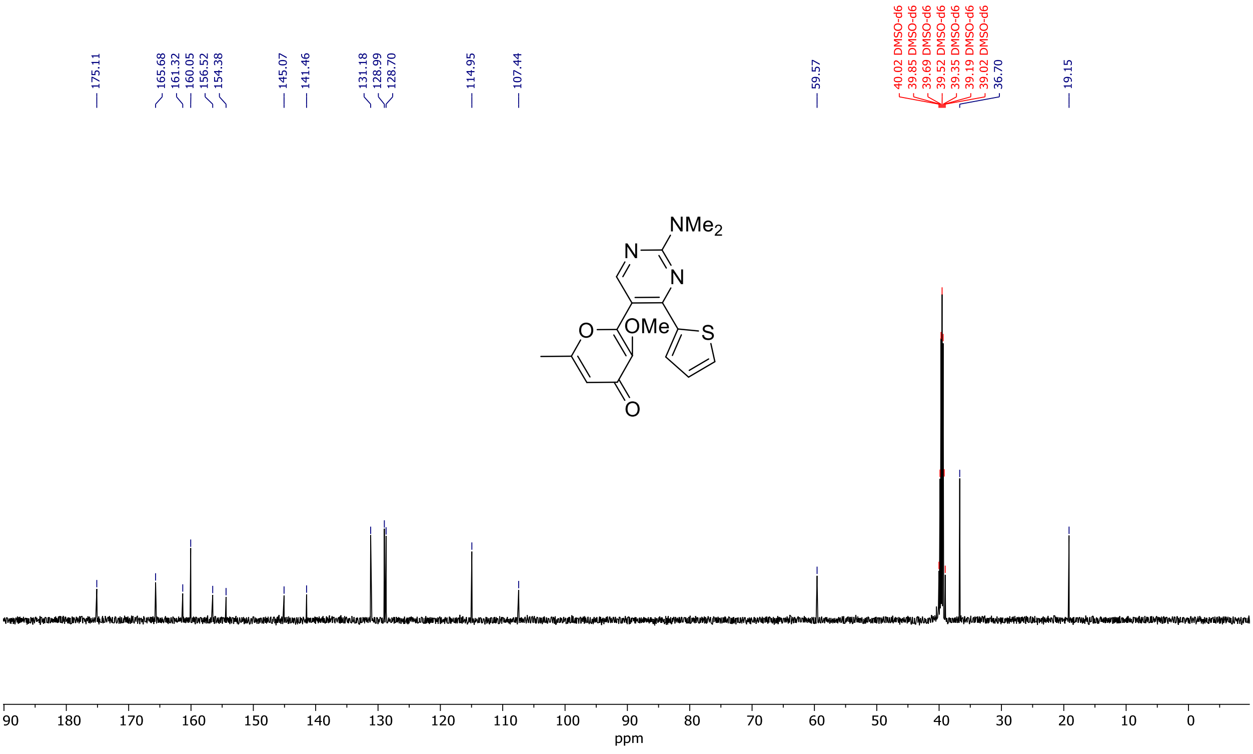

5. NMR ^{1}H and ^{13}C spectra for photoproducts 11 and 12

^{1}H NMR spectrum (300 MHz) of **11a** in DMSO-d_6

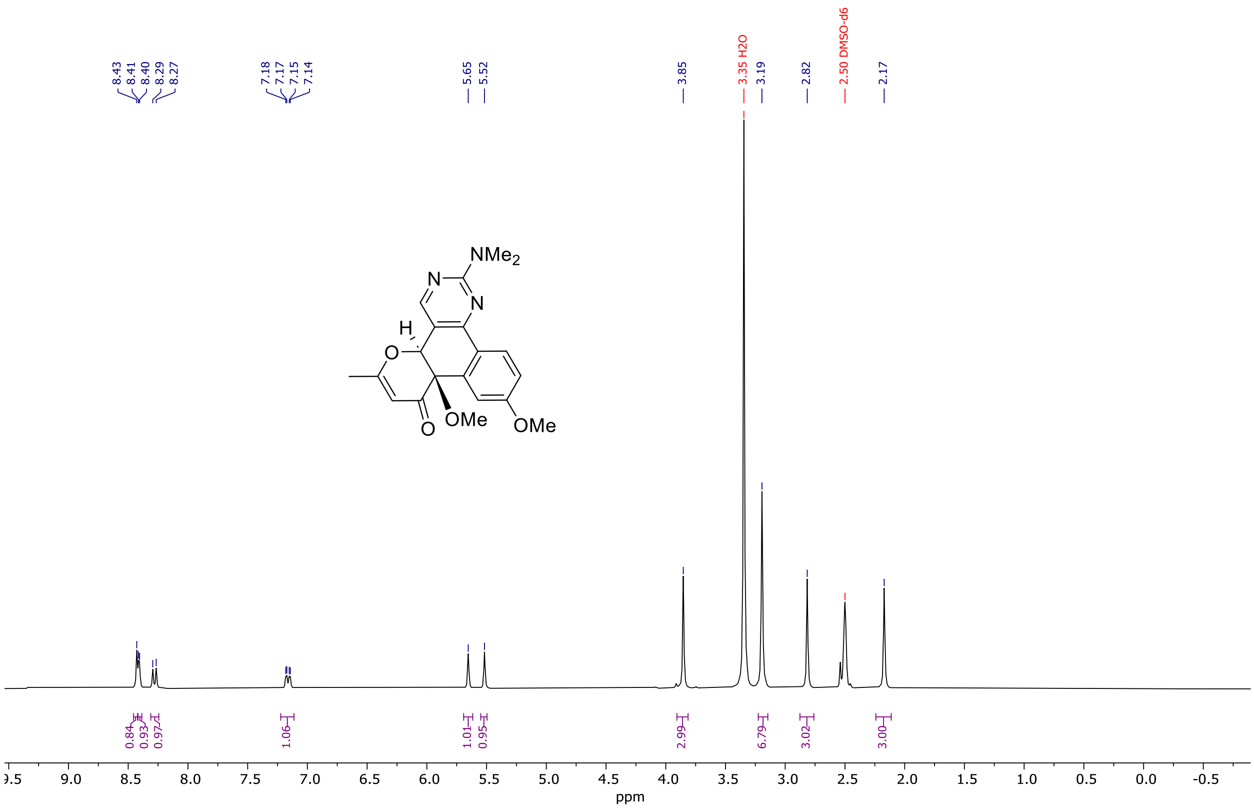

^{13}C {^{1}H} NMR spectrum (75 MHz) of **11a** in DMSO-d_6

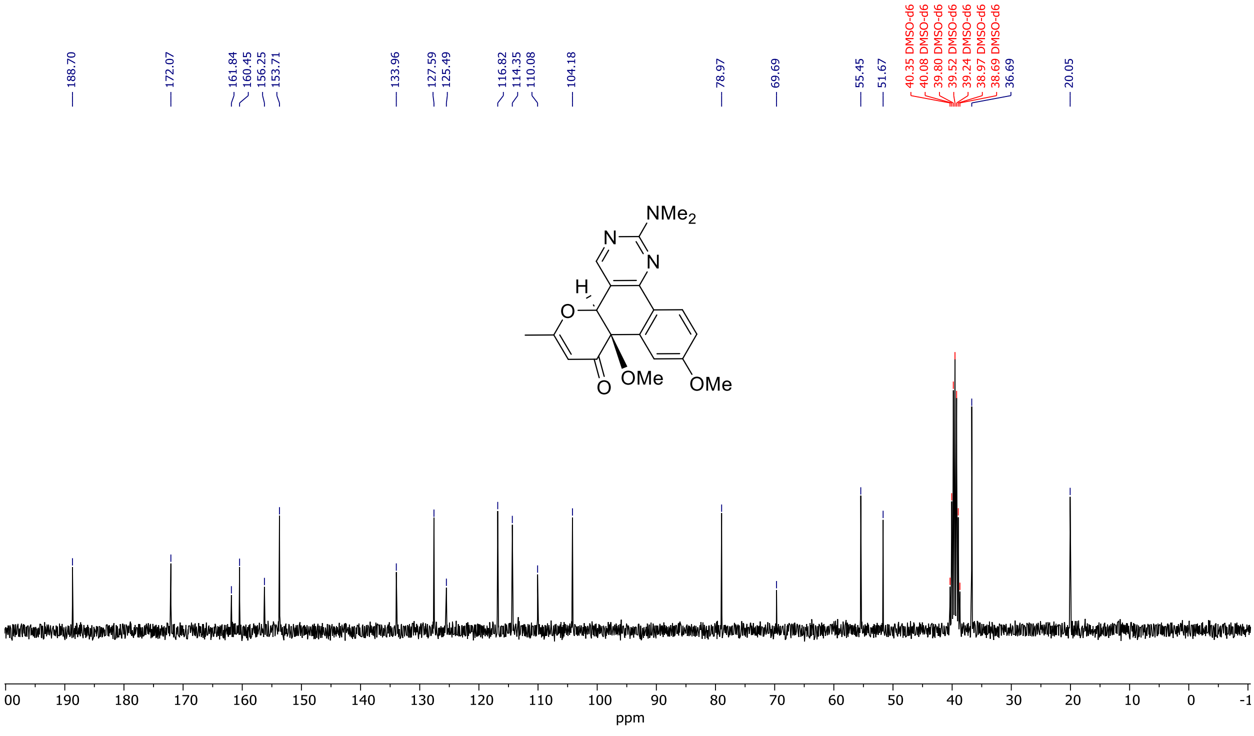

^{1}H NMR spectrum (300 MHz) of **11b** in $CDCl_3$

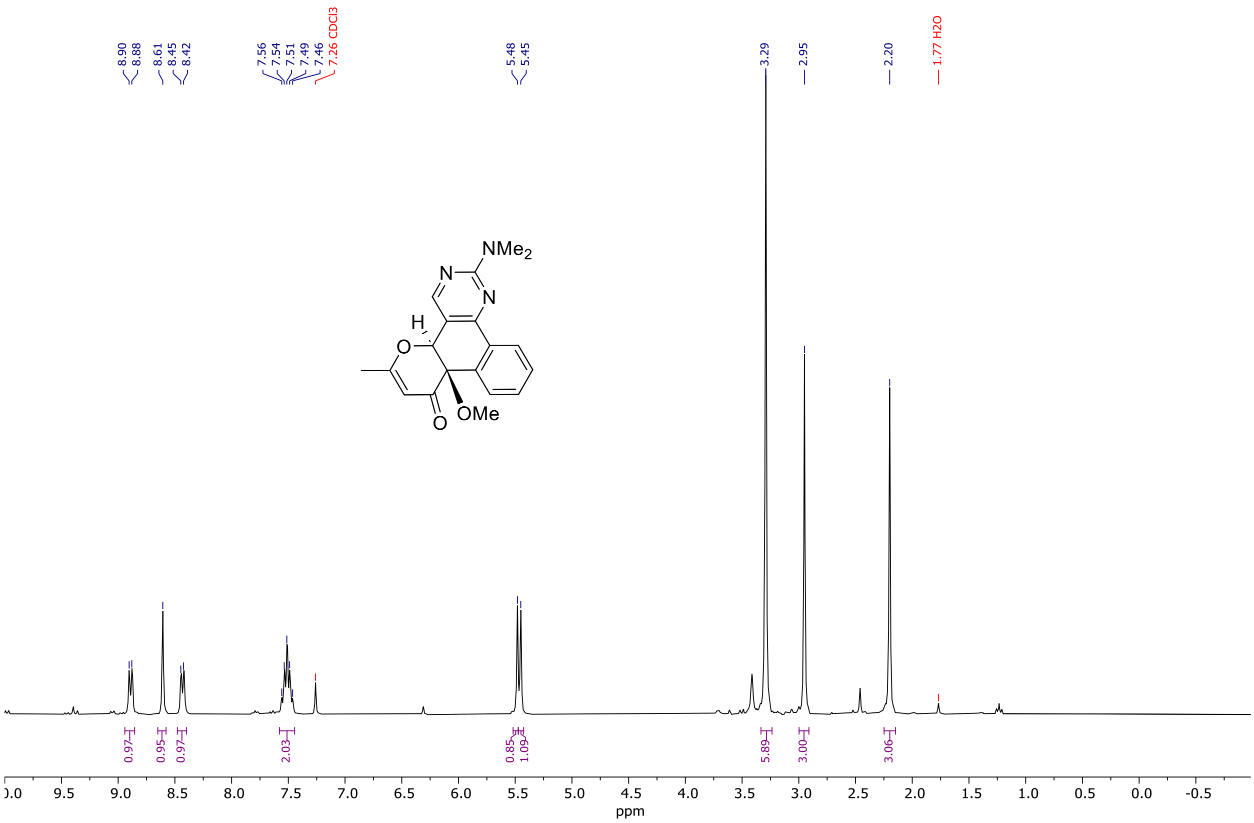

^{13}C {^{1}H} NMR spectrum (75 MHz) of **11b** in $CDCl_3$

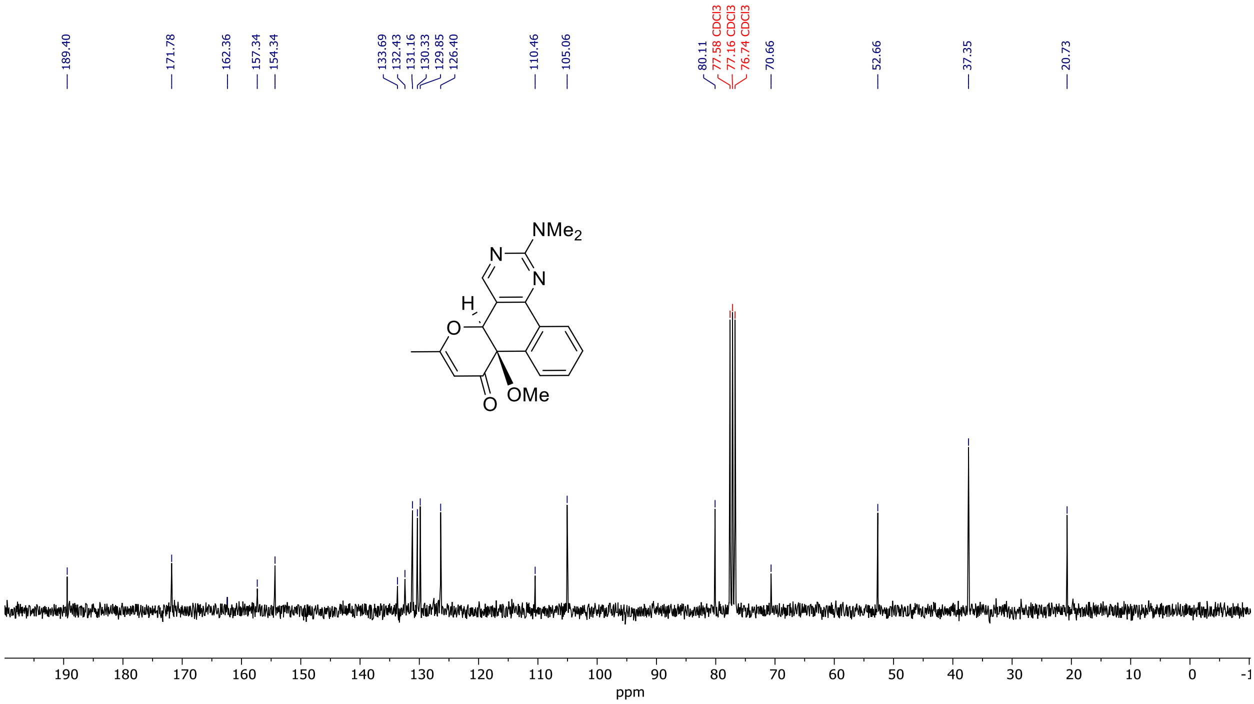

^{1}H NMR spectrum (300 MHz) of **11g** in DMSO-d_6

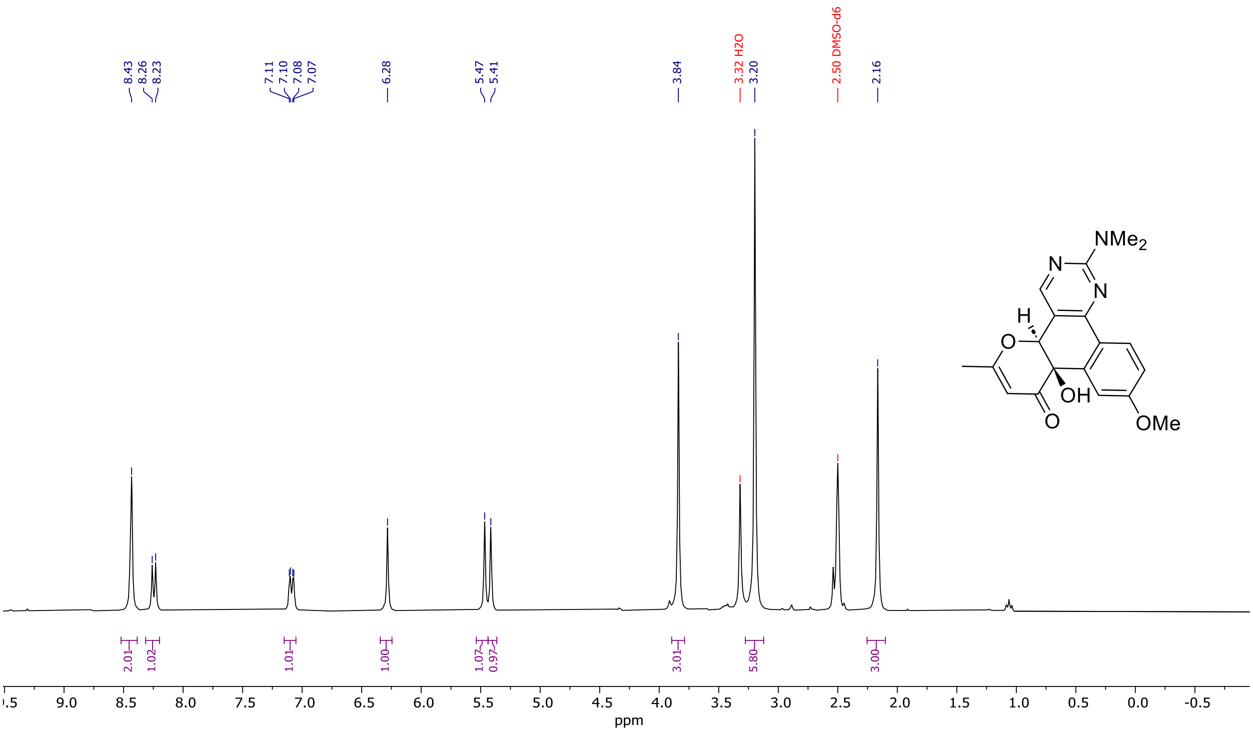

^{13}C {^{1}H} NMR spectrum (75 MHz) of **11g** in DMSO-d_6

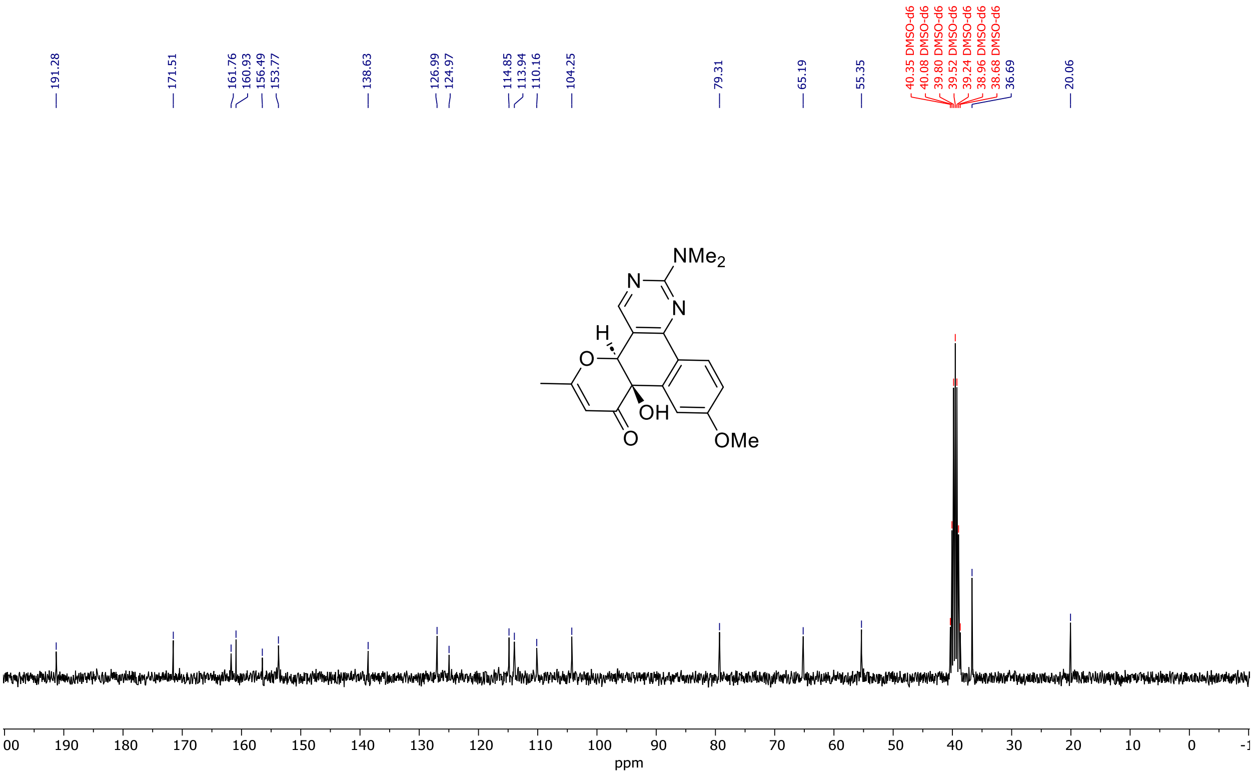

^{1}H NMR spectrum (300 MHz) of **11h** in $CDCl_3$

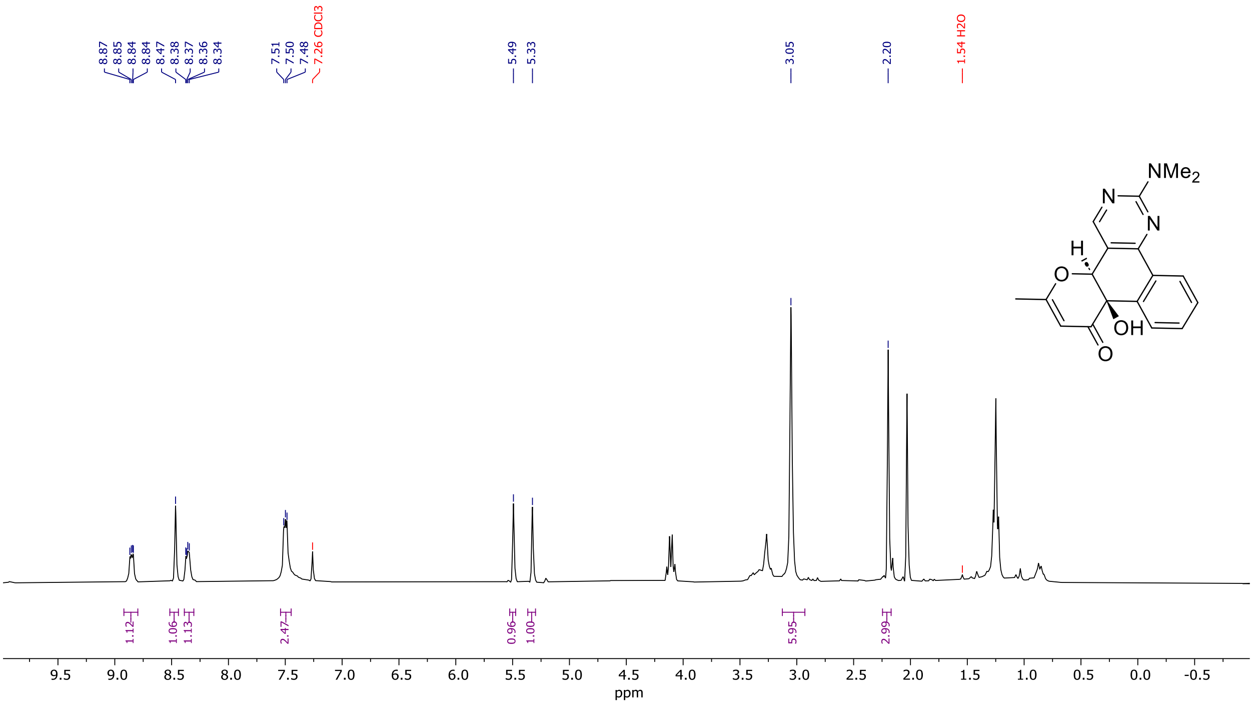

^{13}C {^{1}H} NMR spectrum (75 MHz) of **11h** in $CDCl_3$

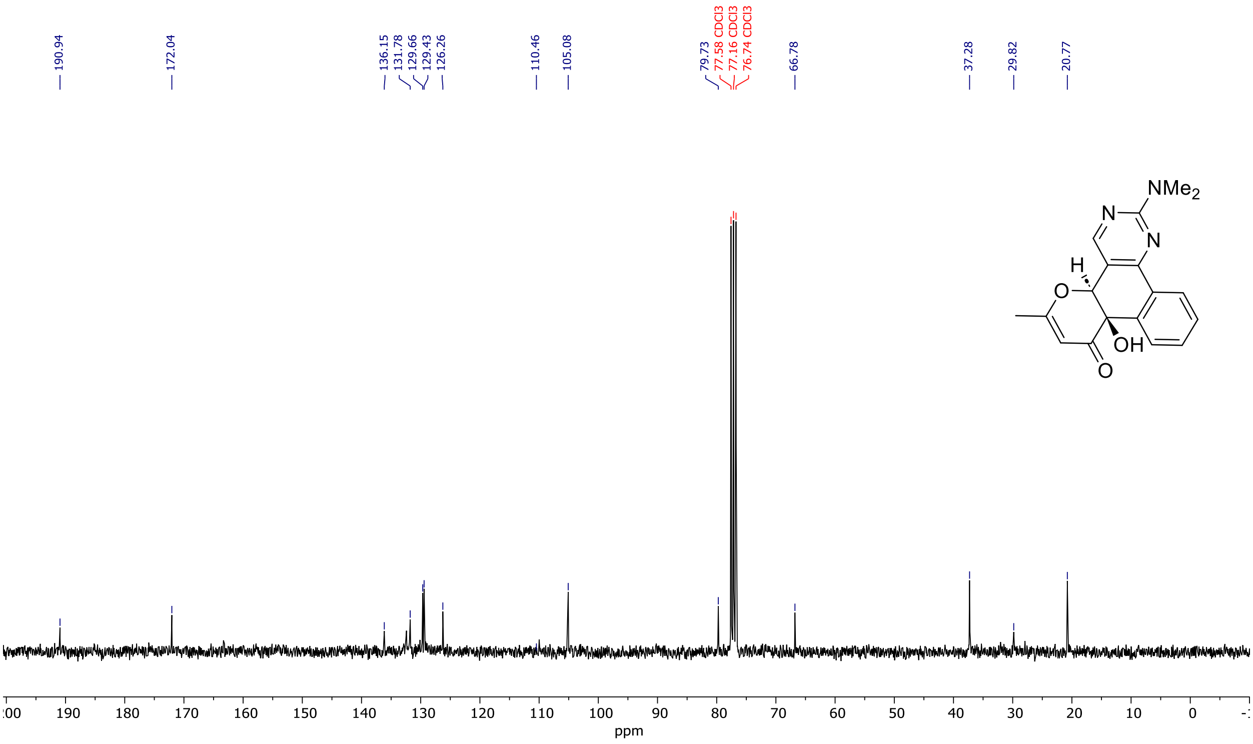

^{1}H NMR spectrum (300 MHz) of **11i** in DMSO-d_6

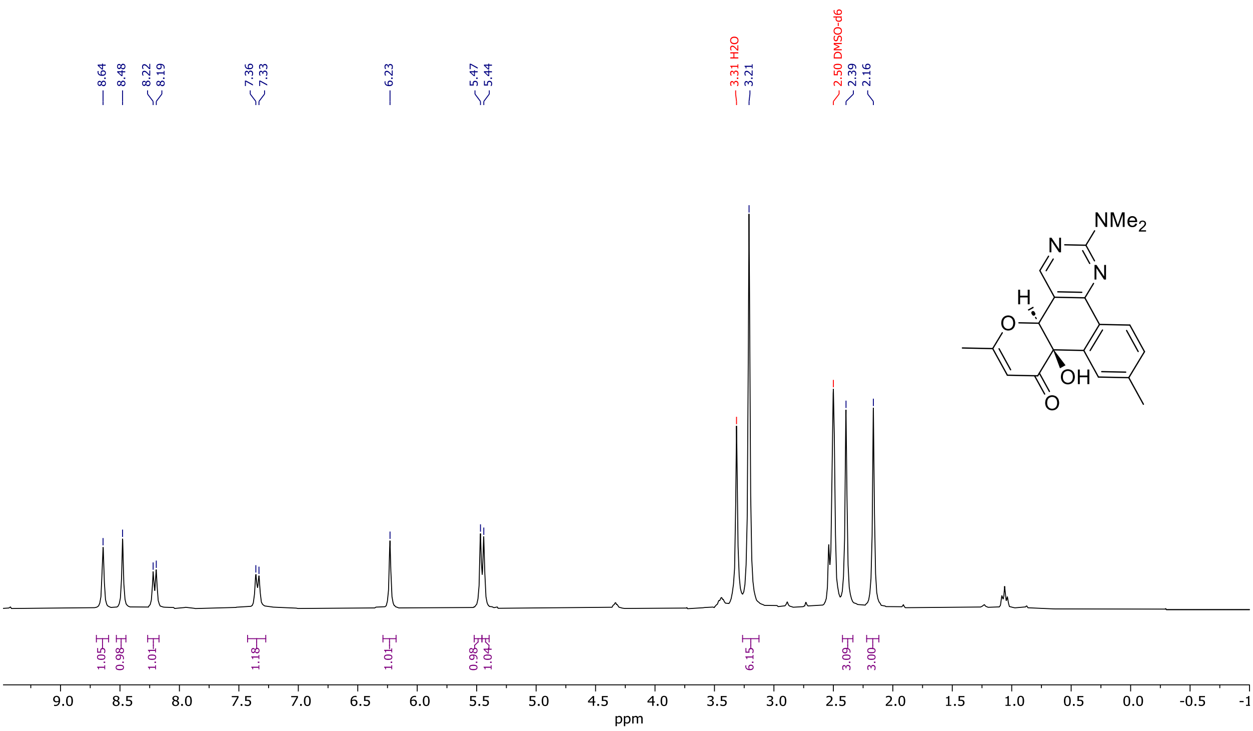

^{13}C {^{1}H} NMR spectrum (75 MHz) of **11i** in DMSO-d_6

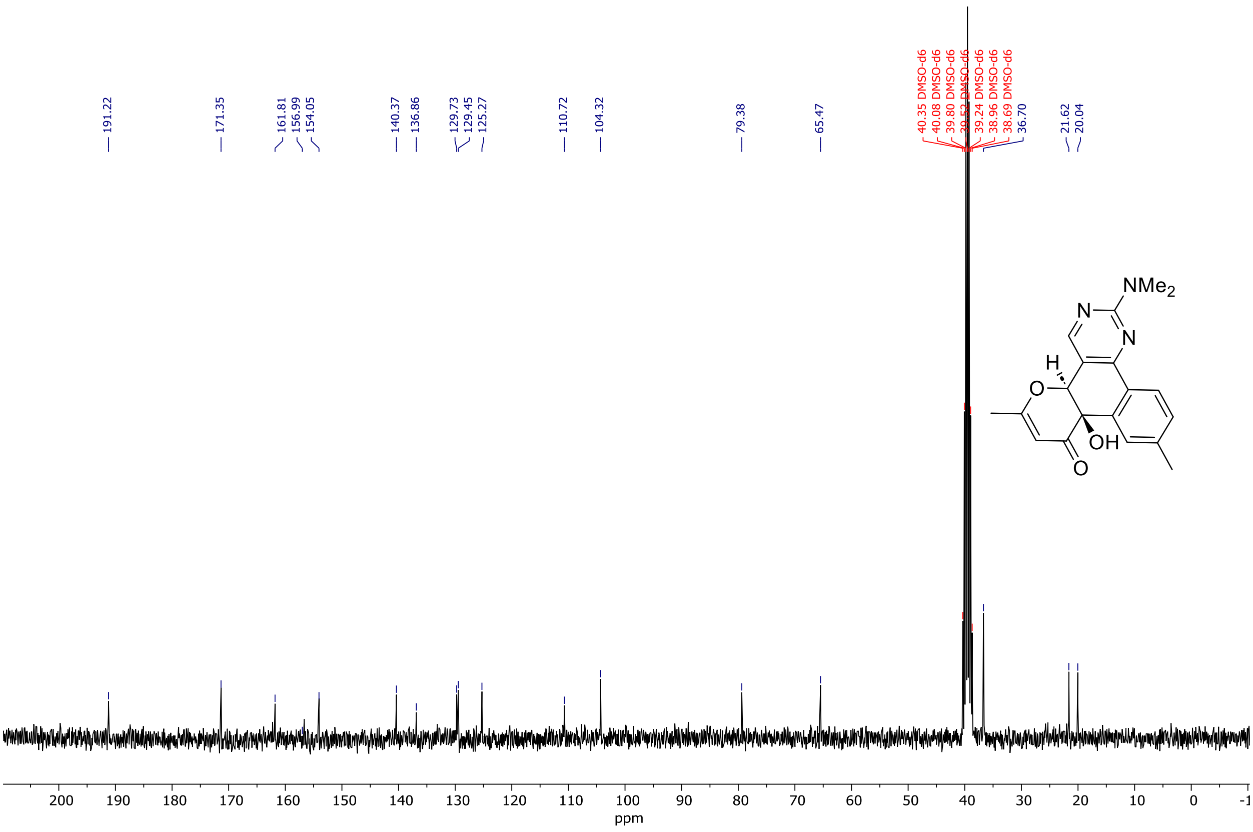

^{1}H NMR spectrum (300 MHz) of **11j** in $CDCl_3$

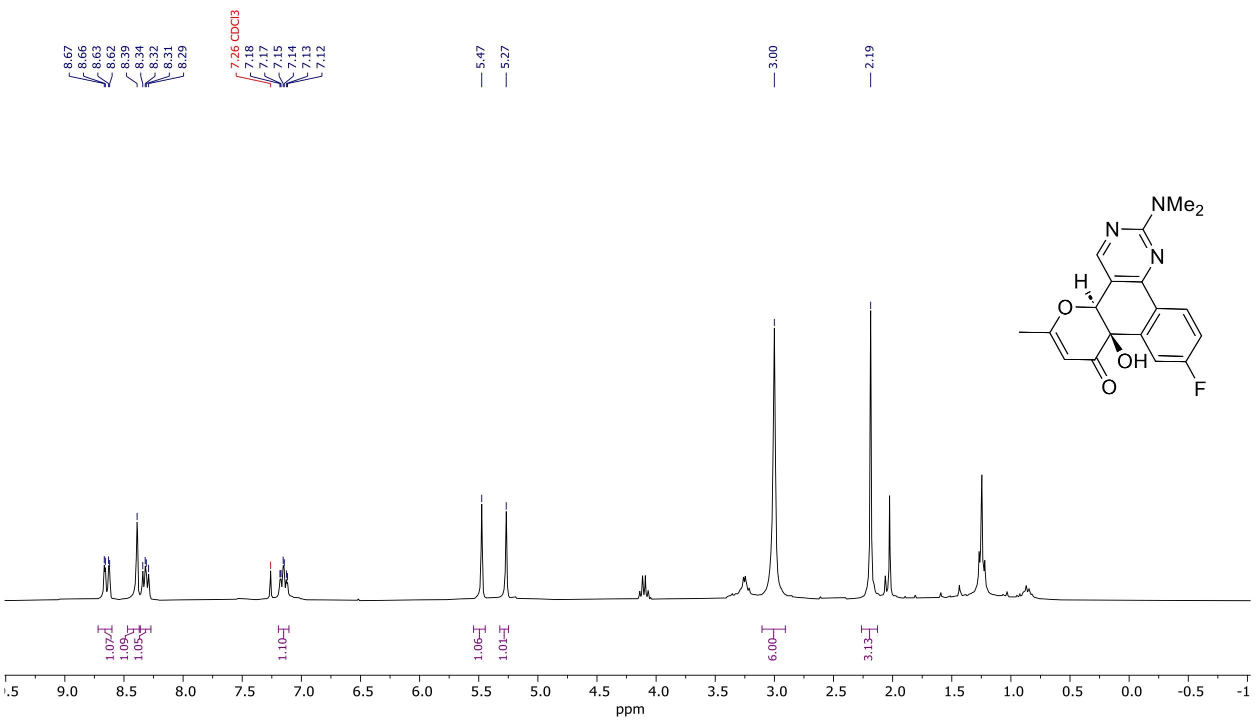

^{13}C {^{1}H} NMR spectrum (75 MHz) of **11j** in $CDCl_3$

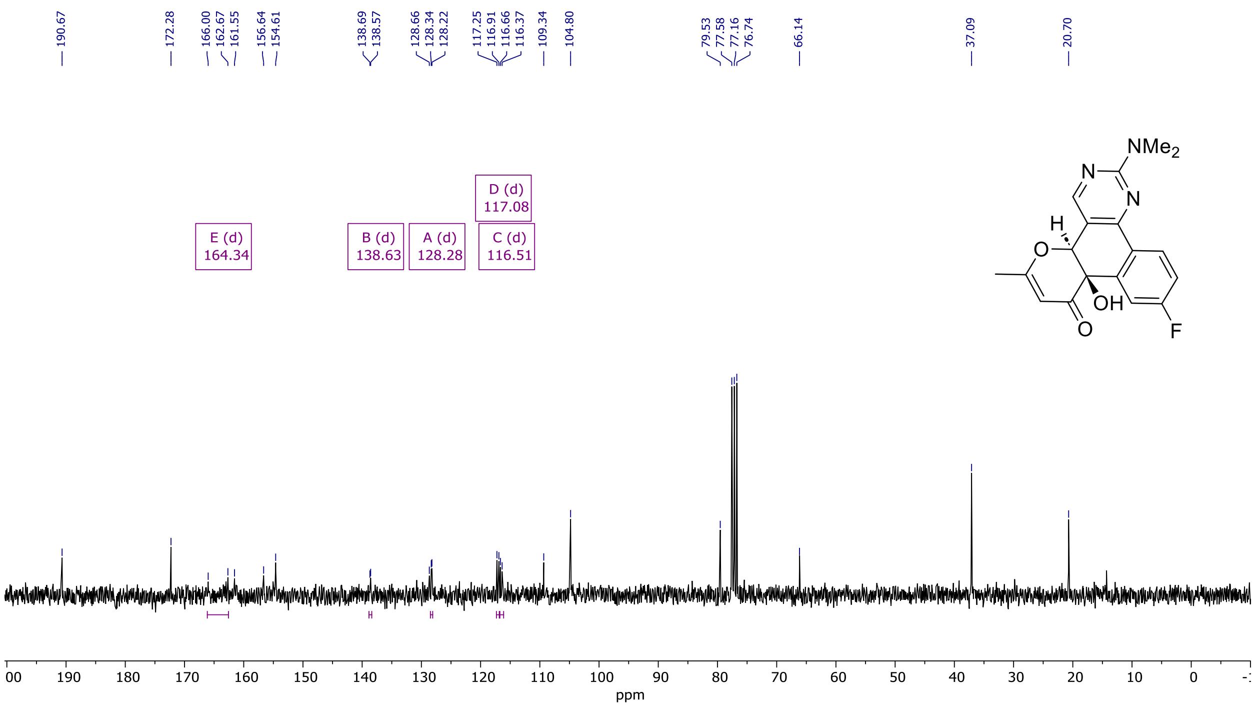

^{1}H NMR spectrum (300 MHz) of **12a** in $CDCl_3$

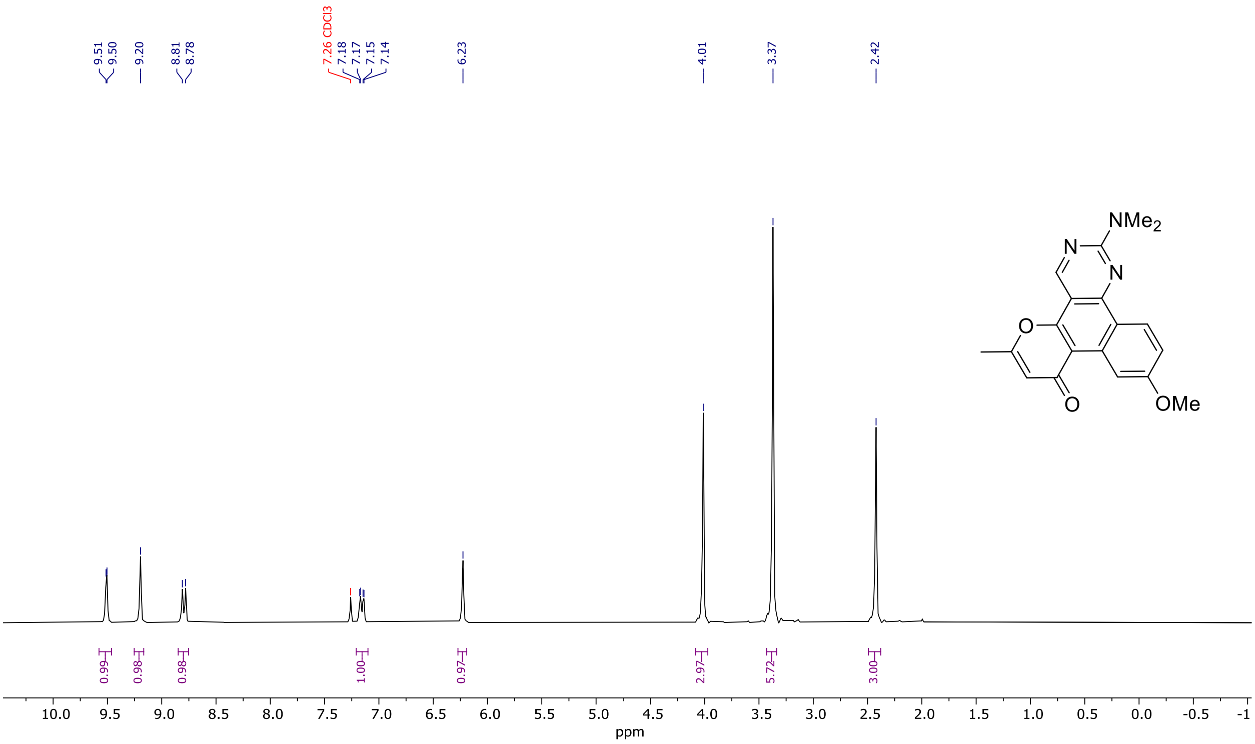

^{13}C {^{1}H} NMR spectrum (151 MHz) of **12a** in $CDCl_3$

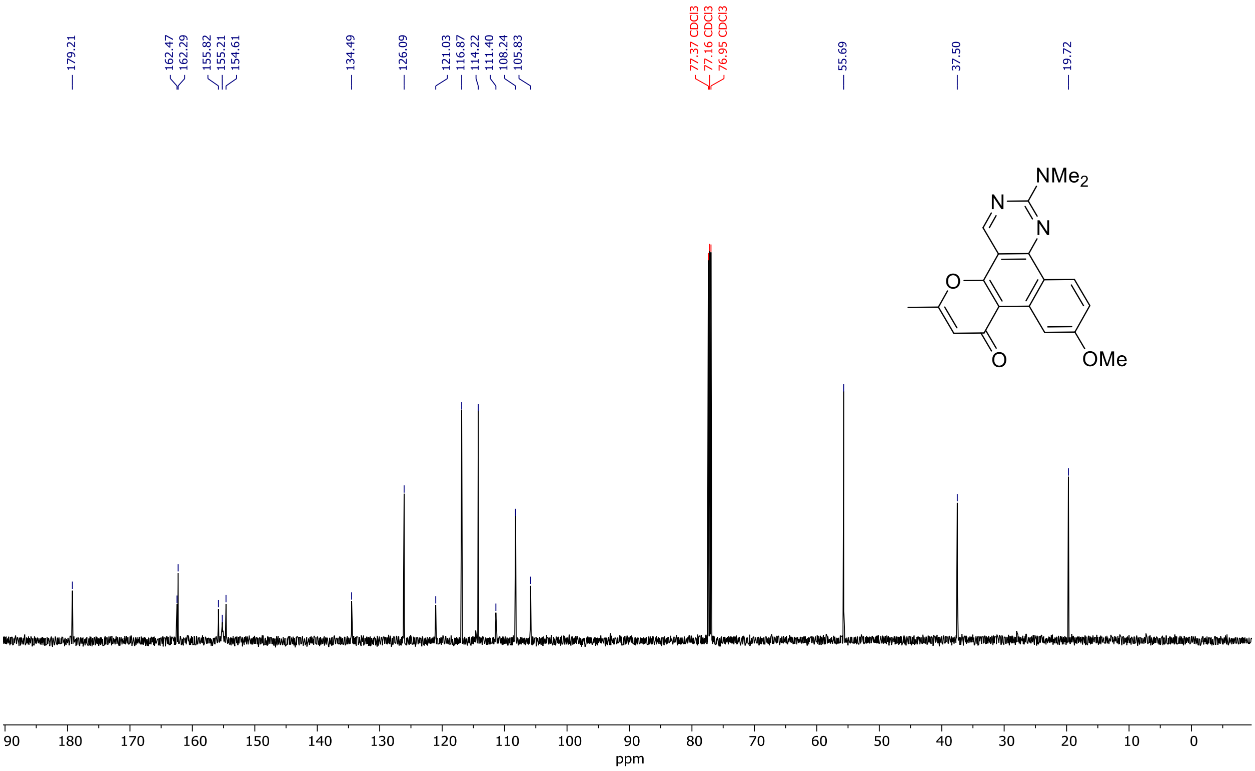

^{1}H NMR spectrum (300 MHz) of **12b** in $CDCl_3$

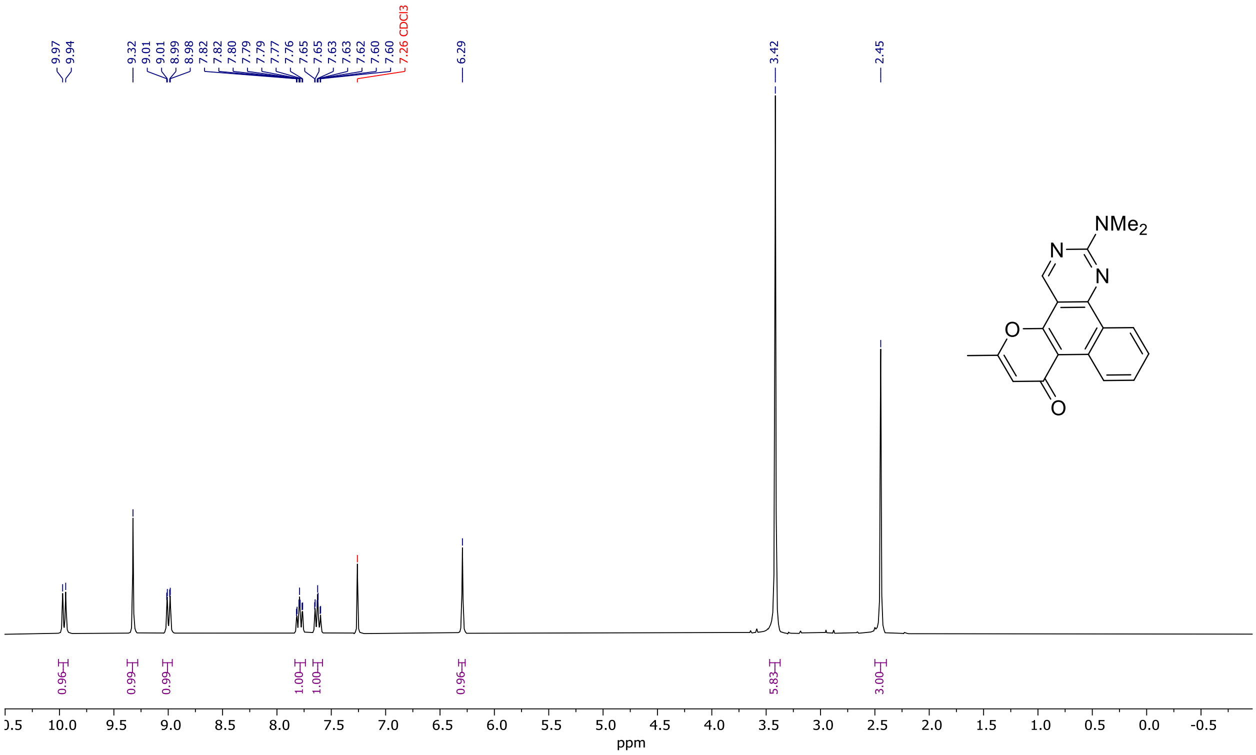

$^{13}C\ \{^{1}H\}$ NMR spectrum (75 MHz) of **12b** in $CDCl_3$

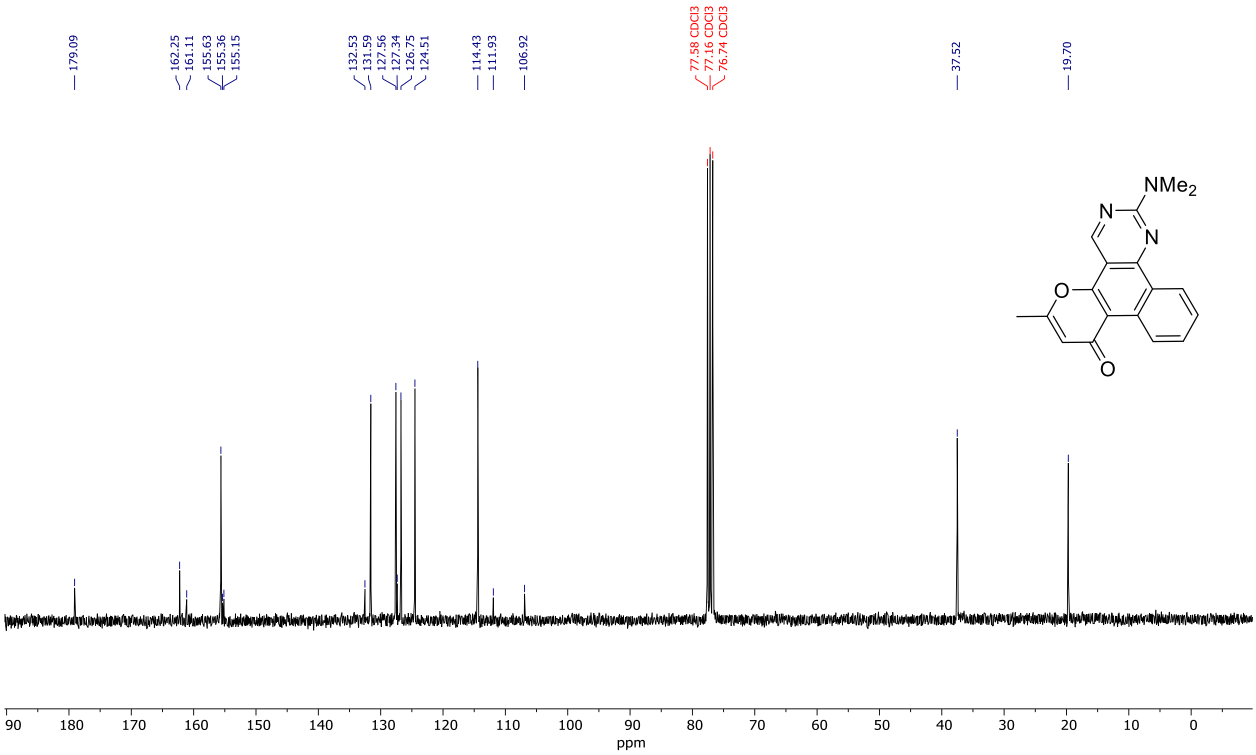

^{1}H NMR spectrum (300 MHz) of **12c** in $CDCl_3$

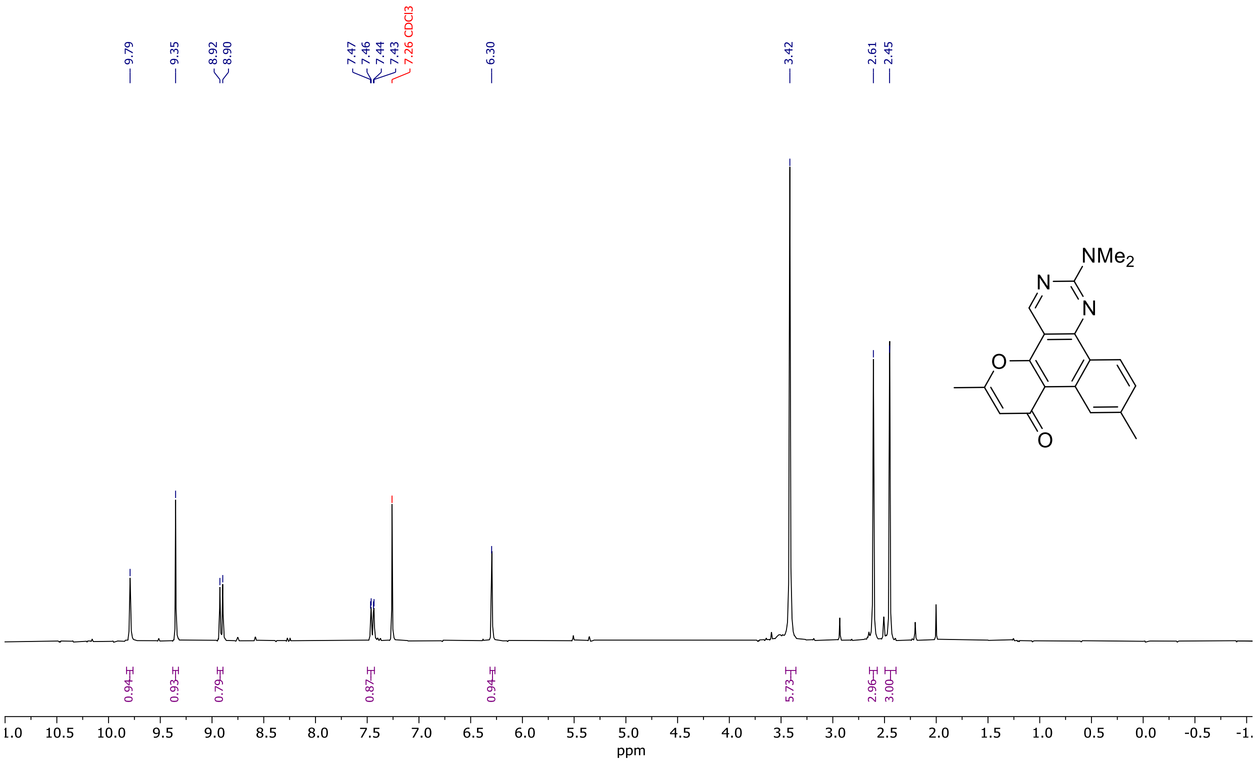

^{13}C {^{1}H} NMR spectrum (126 MHz) of **12c** in $CDCl_3$

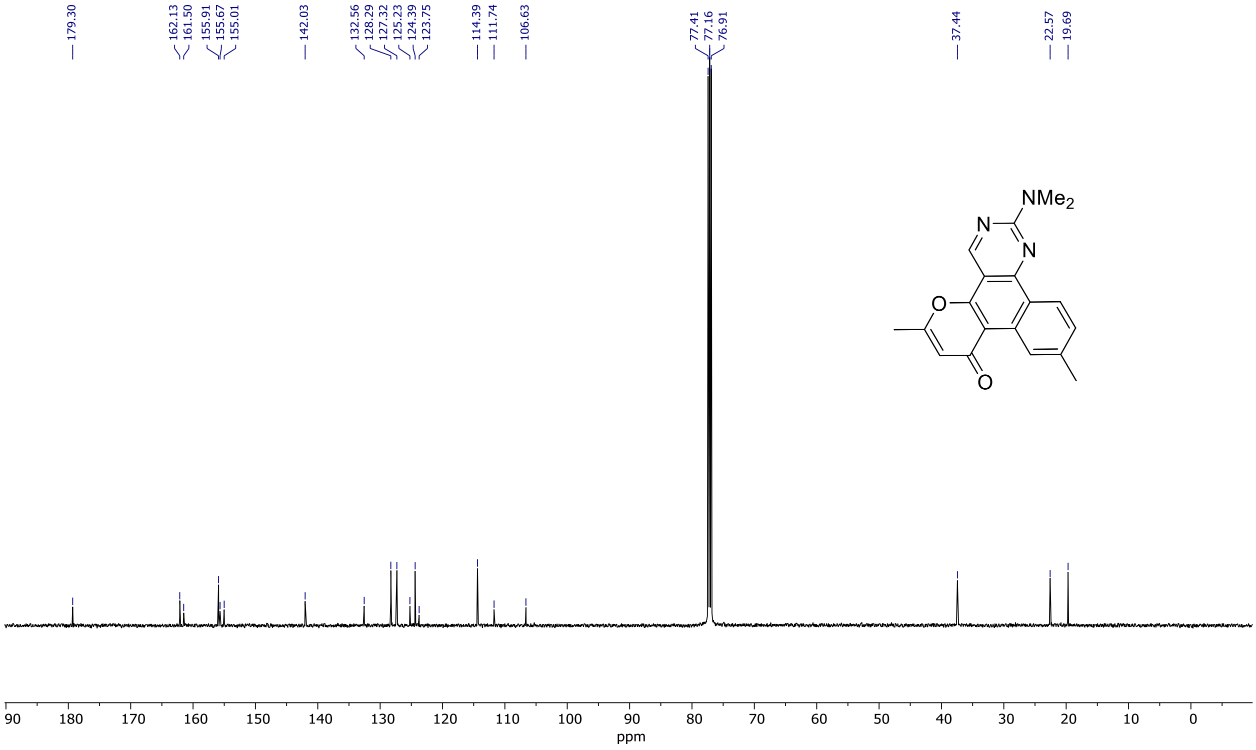

^{1}H NMR spectrum (300 MHz) of **12d** in $CDCl_3$

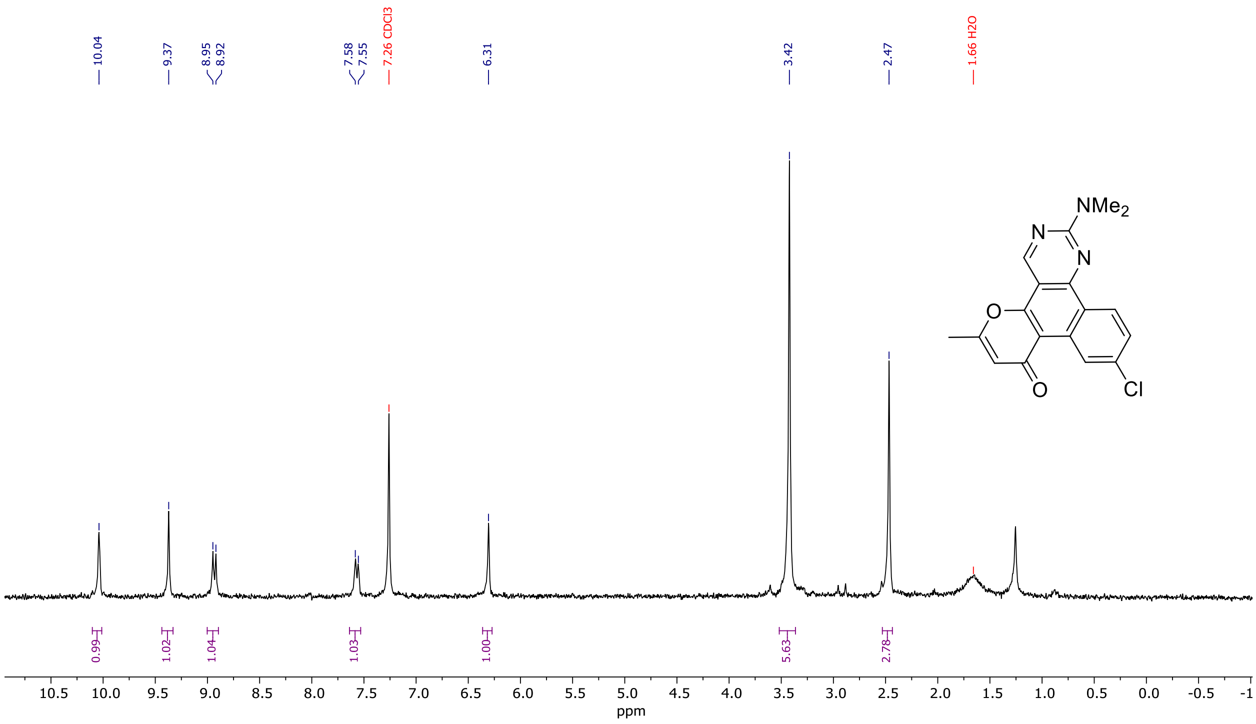

^{13}C {^{1}H} NMR spectrum (126 MHz) of **12d** in $CDCl_3$

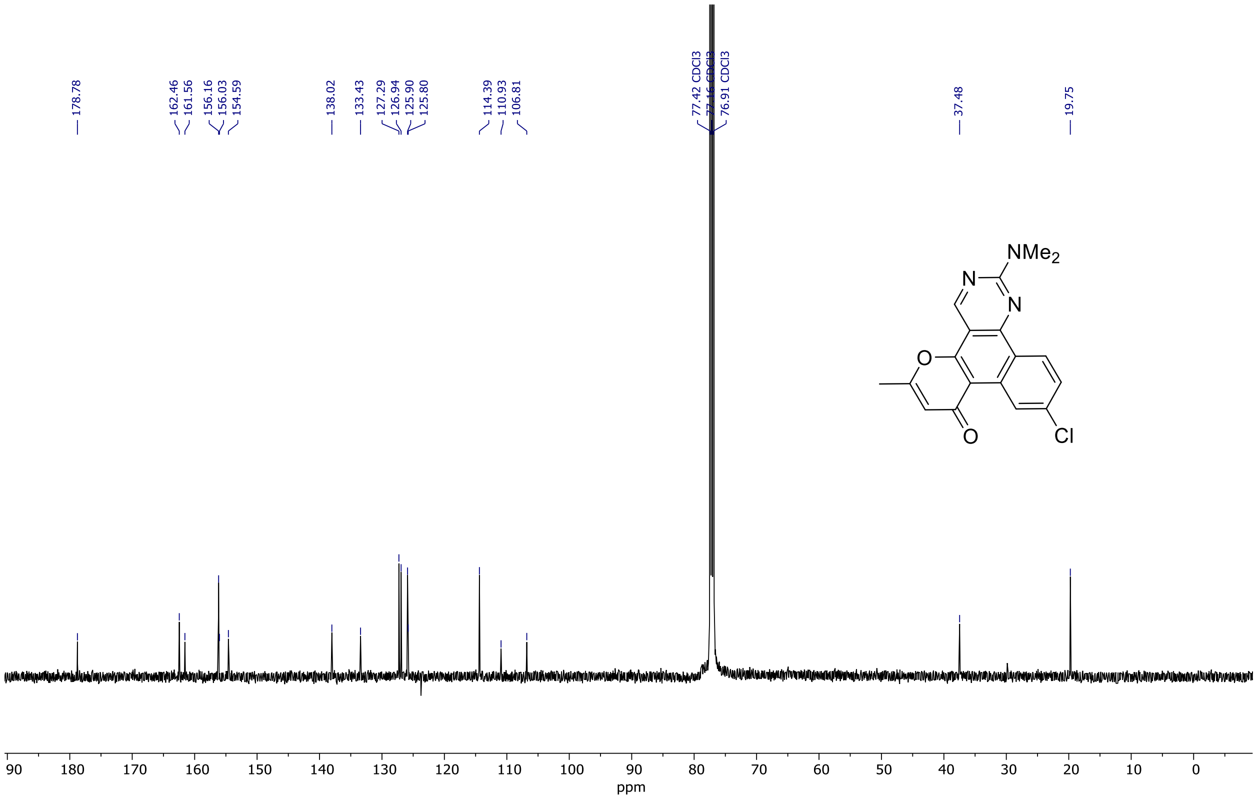

^{1}H NMR spectrum (300 MHz) of **12e** in $CDCl_3$

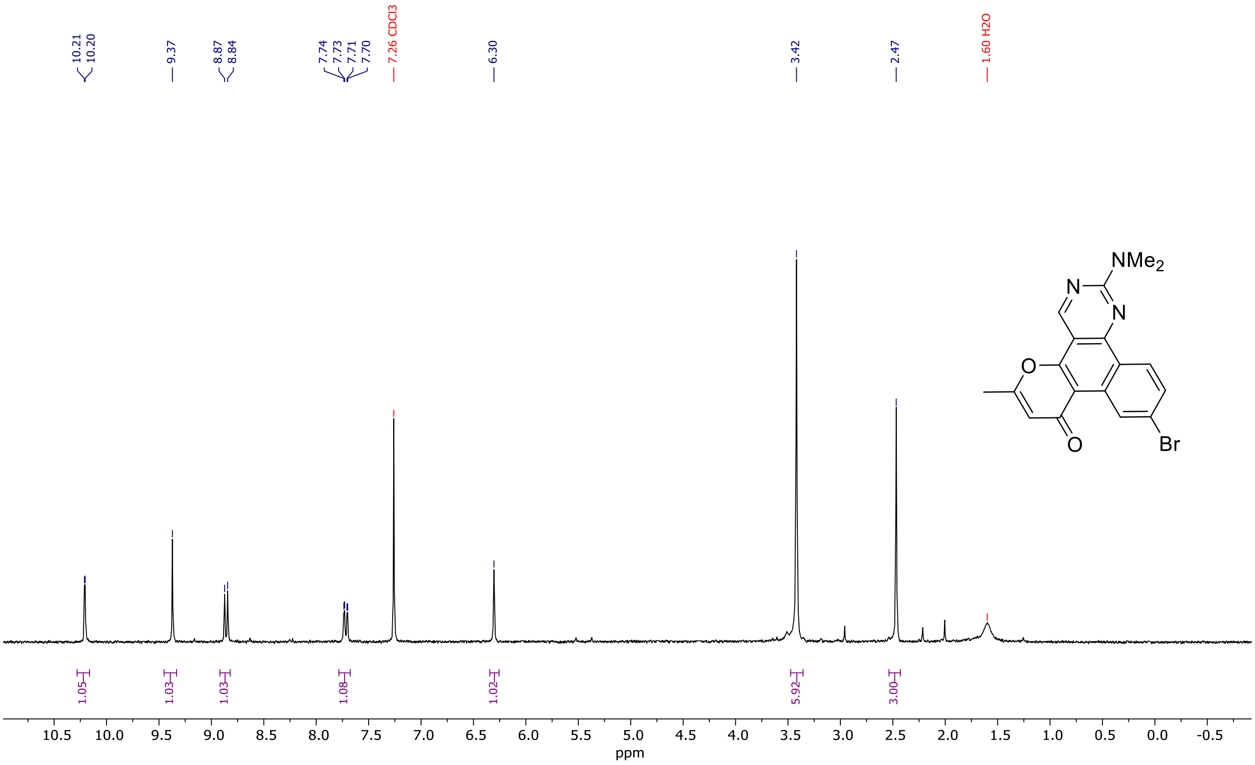

^{13}C {^{1}H} NMR spectrum (126 MHz) of **12e** in $CDCl_3$

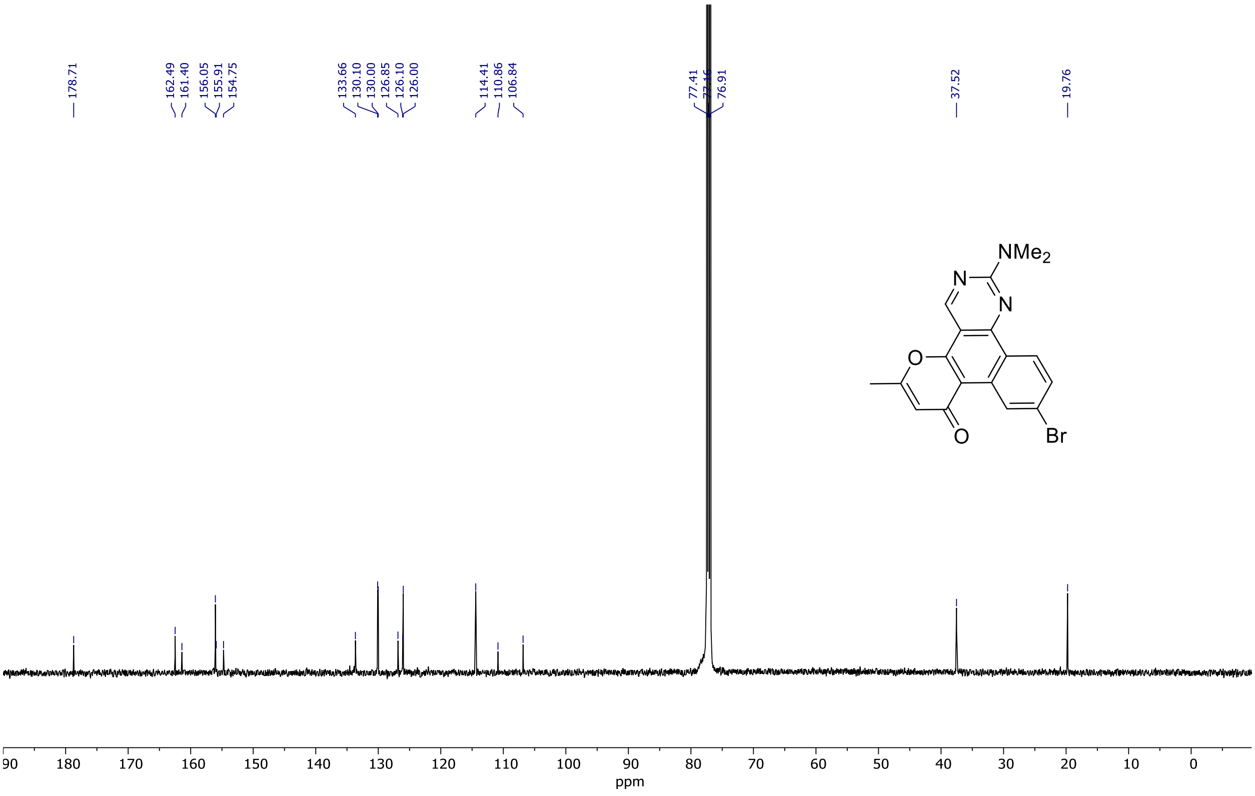

^1H NMR spectrum (300 MHz) of **12f** in DMSO-d_6

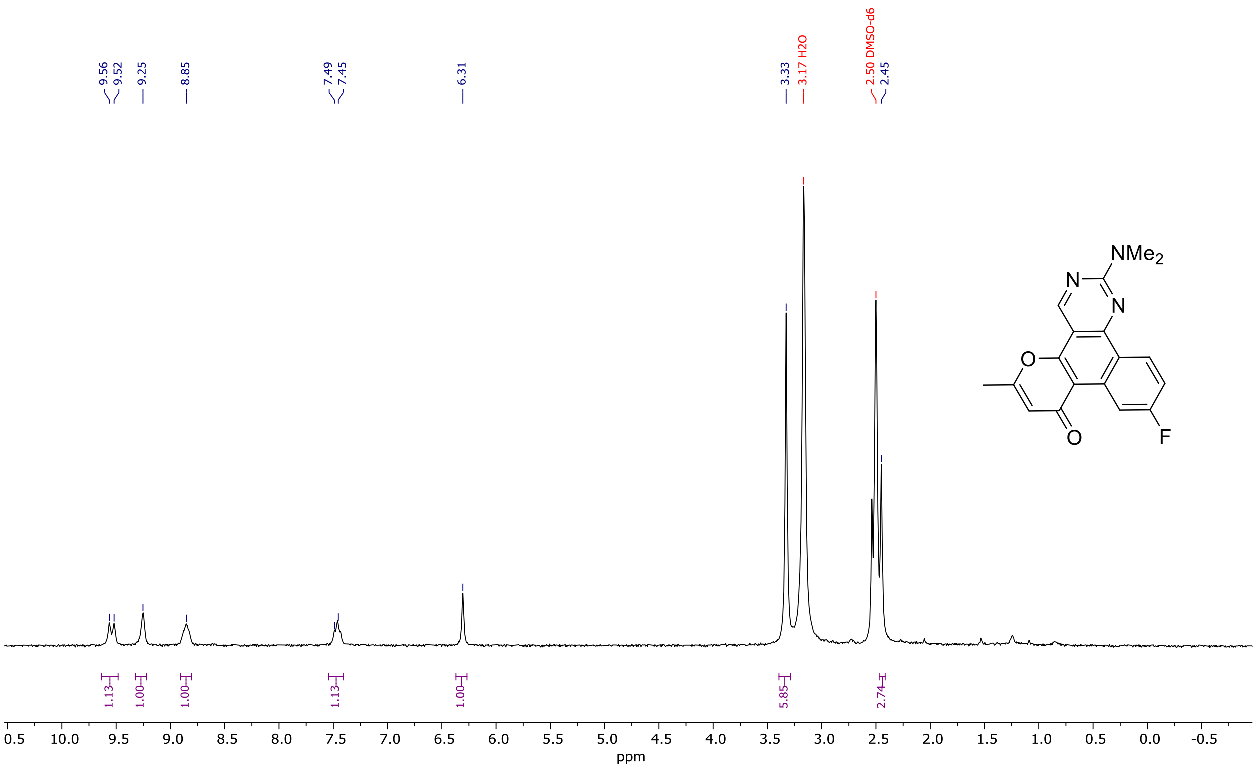

^{13}C {^1H} NMR spectrum (126 MHz) of **12f** in DMSO-d_6

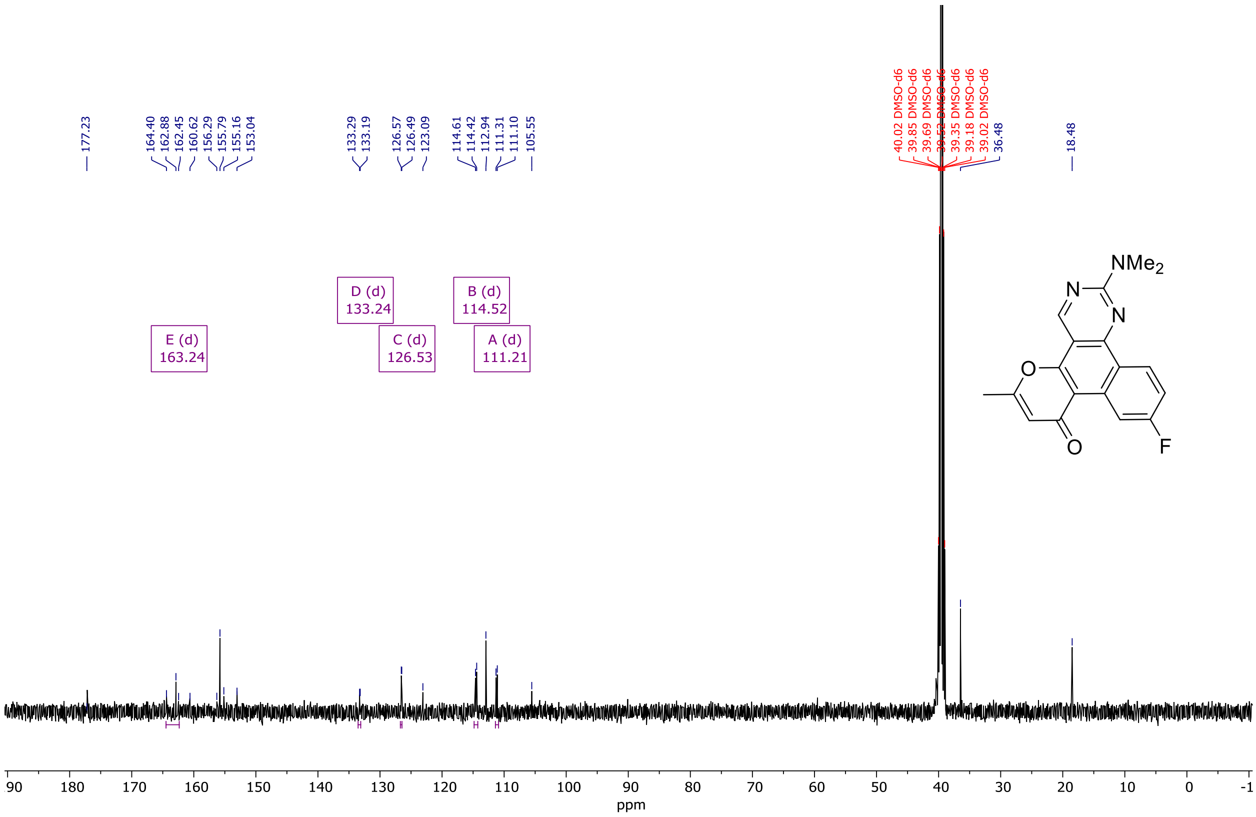

6. X-ray crystallographic data and refinement details

A crystallized specimen of **11a** contained crystals of two different shapes – rods and prisms, which appeared to be different polymorphic modifications. X-ray diffraction data for **11g**·0.5EtOH and for both modifications of **11a** were collected at 100K on a four-circle Rigaku Synergy S diffractometer equipped with a HyPix6000HE area-detector (kappa geometry, shutterless ω-scan technique), using monochromatized Cu Kα-radiation. The intensity data were integrated and analytically corrected for absorption and decay by the CrysAlisPro program.[2] The structures were solved by direct methods using SHELXT[3] and refined by full-matrix least-squares on F^2 using SHELXL-2018[4] in the OLEX2 program.[5] All non-hydrogen atoms were refined with individual anisotropic displacement parameters. Locations of all hydroxy hydrogen atoms in **11g** were found from the electron density-difference map; these hydrogen atoms were refined with individual isotropic displacement parameters. All other hydrogen atoms were placed in ideal calculated positions (C–H distance = 0.950 Å for aromatic, 0.980 Å for methyl, 0.990 Å for methylene, 1.000 Å for cyclopentadienyl, and 1.000 Å for tertiary hydrogen atoms) and refined as riding atoms with relative isotropic displacement parameters (taken as $U_{iso}(H)=1.5U_{eq}(C)$ for methyl, $1.2U_{eq}(C)$ for other hydrogen atoms). A rotating group model was applied for methyl groups. The crystal of the prism modification of **11a** was processed and refined as a regular non-merohedral twin.

Crystal data, data collection and structure refinement details are summarized in Table S1. The structures have been deposited at the Cambridge Crystallographic Data Center with the reference CCDC numbers 2248033-2248035; they also contain the supplementary crystallographic data. These data can be obtained free of charge from the CCDC *via* https://www.ccdc.cam.ac.uk/structures/ .

Table S1. Crystal data, data collection and structure refinement details.

Code	**11a**	**11a**	**11g**•0.5EtOH
Crystal shape	rod	prism	rod
Empirical formula	$C_{20}H_{21}N_3O_4$	$C_{20}H_{21}N_3O_4$	$C_{19}H_{19}N_3O_4$•0.5(C_2H_6O)
Formula weight	367.40	367.40	376.40
Temperature, K	99.9(3)	99.9(3)	100.0(1)
Wavelength, Å	1.54184	1.54184	1.54184
Crystal system	Monoclinic	Triclinic	Triclinic
Space group	$P2_1/c$	$P\bar{1}$	$P\bar{1}$
Unit cell dimensions			
a, Å	14.02520(10)	8.52217(11)	16.08584(4)
b, Å	12.13380(10)	9.33488(11)	20.06358(5)
c, Å	20.89040(10)	12.93524(16)	33.61021(8)
α, °	90	78.6790(10)	88.0844(2)
β, °	94.5520(10)	74.8488(11)	81.2905(2)
γ, °	90	62.9464(12)	80.7090(2)
Volume, $Å^3$	3543.89(4)	880.99(2) $Å^3$	10581.15(4)
Z / Z'	8 / 2	2 / 1	24 / 12
Density (calculated), $g \cdot cm^{-3}$	1.377	1.385	1.418
Absorption coefficient (μ), mm^{-1}	0.801	0.805	0.839
F(000)	1552	388	4776
Crystal size, mm	0.53×0.13×0.08	0.49×0.26×0.08	0.74×0.25×0.20
θ range, °	3.161-76.972	5.343-77.803	2.231-79.647
Indexranges	-15<=h<=17,	-9<=h<=10,	-20<=h<=20,
	-15<=k<=15,	-11<=k<=11,	-25<=k<=25,
	-26<=l<=26	-15<=l<=16	-39<=l<=42
Reflections			
Collected	76143	11169*	331136
Independent [R_{int}]	7475 [0.0440]	11169 [0*]	45527 [0.0417]
Observed (I>2σ)	6923	10441	44246
Completeness to $θ_{full}$ (67.68°)	1.000	0.996	0.999
T_{max} / T_{min}	0.953 / 0.787	0.941 / 0.761	0.874 / 0.694
Data / restraints / parameters	7475 / 0 / 497	11169 / 0 / 250	45527 / 1 / 3102
Goodness-of-fit on F^2	1.065	1.099	1.062
R1 / wR2 for I>2σ(I)	0.0587 / 0.1689	0.0607 / 0.1847	0.0429 / 0.1174
R1 / wR2 (all data)	0.0606 / 0.1715	0.0627 / 0.1878	0.0439 / 0.1183
$Δρ_{max}$ / $Δρ_{min}$, $ē \cdot Å^{-3}$	0.698 / -0.362	0.408 / -0.269	0.383 / -0.325
CCDC number	2248034	2248033	2248035

** Due to the refinement of **11a**(prism) as a regular non-merohedral twin, equivalent reflections were merged by a conventional way, setting the collected reflection number to be equal to the number of independent reflections and, therefore, making R_{int}=0.

The structure of **11a**

The asymmetric unit of the monoclinic rod-shaped modification of **11a** consists of two crystallographically independent molecules of **11a** (Figure S1). Their conformations are nearly identical (RMSD = 0.0443 Å), in which positions of atoms C16A/C16B and C19A/C19B slightly differ. The triclinic prism-shaped modification of **11a** contains only one independent molecule (Figure S2).

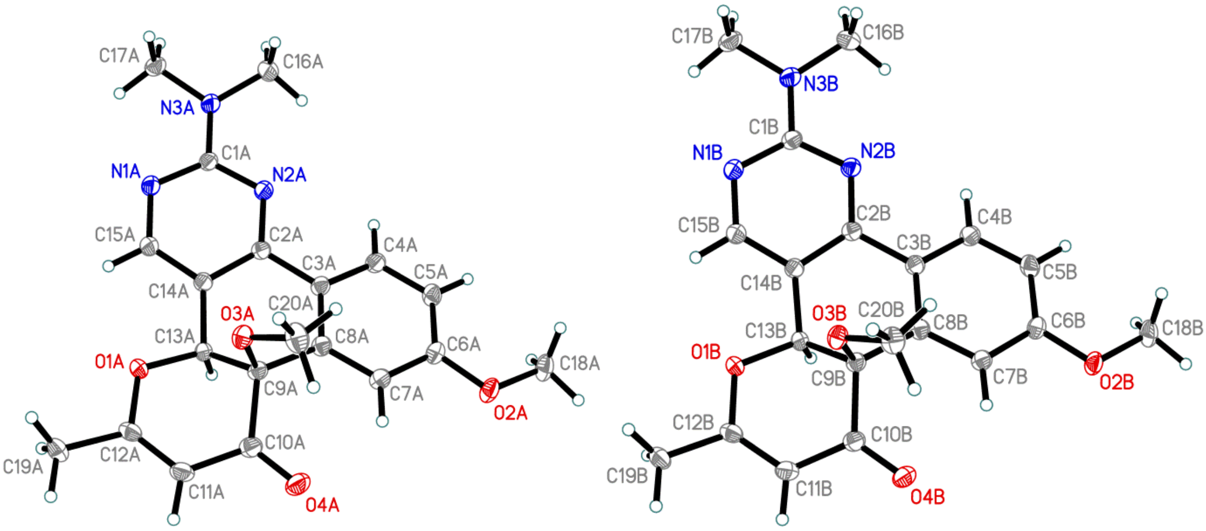

Figure S1. Two unique molecules of **11a** of its rod-shaped modification.

Table S2. Selected bond distances in **11a** (rod-shaped modification), Å

O1A-C12A	1.3485(14)	N2A-C2A	1.3354(15)	C6A-C7A	1.4021(17)
O1A-C13A	1.4375(13)	N3A-C1A	1.3577(15)	C7A-C8A	1.3872(17)
O2A-C6A	1.3652(15)	N3A-C16A	1.4501(16)	C8A-C9A	1.5324(16)
O2A-C18A	1.4349(15)	N3A-C17A	1.4522(15)	C9A-C10A	1.5514(16)
O3A-C9A	1.4385(14)	C2A-C3A	1.4729(16)	C9A-C13A	1.5298(16)
O3A-C20A	1.4290(15)	C2A-C14A	1.3999(16)	C10A-C11A	1.4544(17)
O4A-C10A	1.2227(16)	C3A-C4A	1.3950(16)	C11A-C12A	1.3468(18)
N1A-C1A	1.3524(15)	C3A-C8A	1.4156(16)	C12A-C19A	1.4890(17)
N1A-C15A	1.3329(16)	C4A-C5A	1.3890(17)	C13A-C14A	1.4917(15)
N2A-C1A	1.3526(15)	C5A-C6A	1.3914(18)	C14A-C15A	1.3811(16)
O1B-C12B	1.3500(14)	N2B-C2B	1.3333(15)	C6B-C7B	1.4041(16)
O1B-C13B	1.4365(13)	N3B-C1B	1.3549(15)	C7B-C8B	1.3873(16)
O2B-C6B	1.3647(14)	N3B-C16B	1.4512(16)	C8B-C9B	1.5337(16)
O2B-C18B	1.4335(14)	N3B-C17B	1.4522(16)	C9B-C10B	1.5507(16)
O3B-C9B	1.4372(14)	C2B-C3B	1.4721(16)	C9B-C13B	1.5299(16)
O3B-C20B	1.4271(15)	C2B-C14B	1.4003(16)	C10B-C11B	1.4537(17)
O4B-C10B	1.2246(15)	C3B-C4B	1.3950(16)	C11B-C12B	1.3470(18)
N1B-C1B	1.3550(16)	C3B-C8B	1.4170(16)	C12B-C19B	1.4905(17)
N1B-C15B	1.3323(16)	C4B-C5B	1.3901(17)	C13B-C14B	1.4919(15)
N2B-C1B	1.3513(15)	C5B-C6B	1.3898(17)	C14B-C15B	1.3808(16)

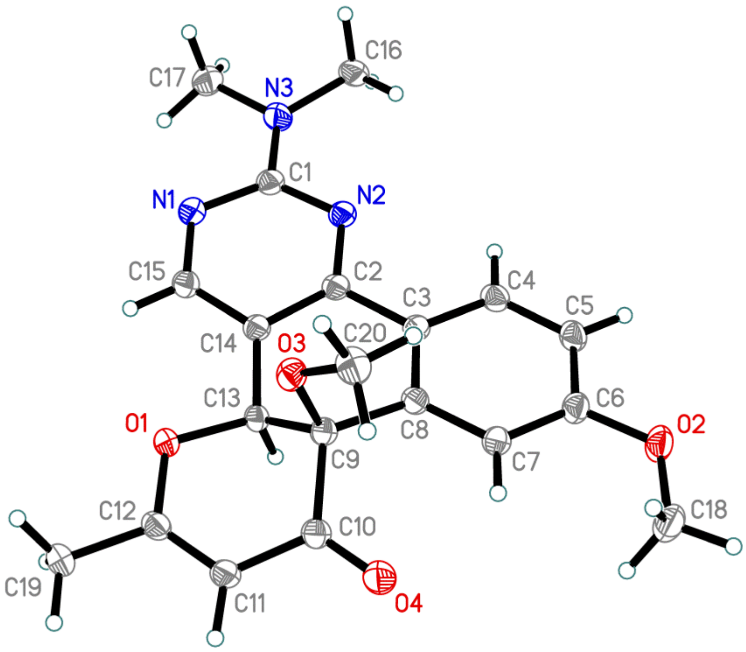

Figure S2. Molecule of **11a** of its prism-shaped modification.

Table S3. Selected bond distances in **11a** (prism-shaped modification), Å

O1-C12	1.3498(17)	N2-C2	1.3366(19)	C6-C7	1.394(2)
O1-C13	1.4445(16)	N3-C1	1.356(2)	C7-C8	1.400(2)
O2-C6	1.3620(19)	N3-C16	1.4555(19)	C8-C9	1.5345(19)
O2-C18	1.4249(19)	N3-C17	1.453(2)	C9-C10	1.5514(19)
O3-C9	1.4397(19)	C2-C3	1.476(2)	C9-C13	1.531(2)
O3-C20	1.4231(19)	C2-C14	1.397(2)	C10-C11	1.452(2)
O4-C10	1.2237(19)	C3-C4	1.403(2)	C11-C12	1.343(2)
N1-C1	1.355(2)	C3-C8	1.405(2)	C12-C19	1.494(2)
N1-C15	1.3330(19)	C4-C5	1.380(2)	C13-C14	1.4948(18)
N2-C1	1.353(2)	C5-C6	1.394(2)	C14-C15	1.391(2)

The structure of **11g**

The asymmetric unit of the crystal **11g**·½EtOH consists of 12 non-equivalent molecules and 6 ethanol molecules (Figure S3). One of the molecules **11g** is shown in Figure S4; bond distances for that molecule are listed in Table S4. The other eleven molecules have similar conformations, but the methoxy group in some molecules exhibits an opposite orientation. All molecules take part in formation hydrogen bonds, geometrical parameters for all hydrogen bonds are listed in Table S5.

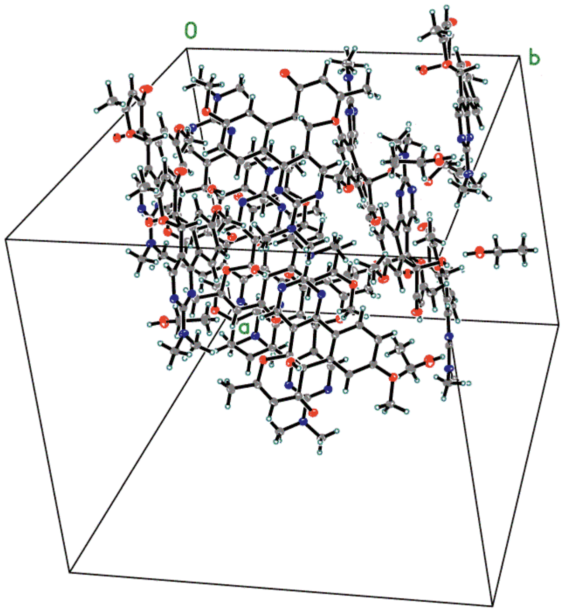

Figure S3. The asymmetric unit of the crystal **11g**.

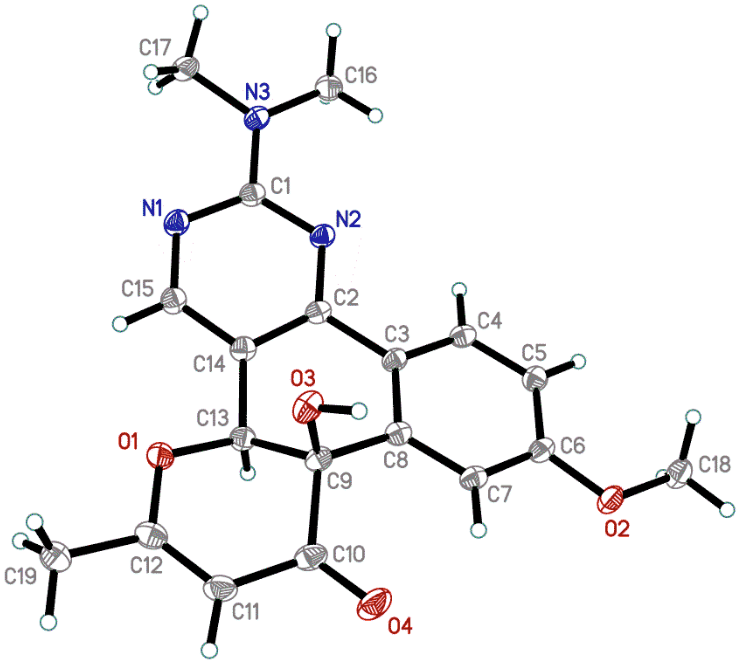

Figure S4. The structure of one of crystallographically unique molecules of **11g**.

Table S4. Selected bond distances for one molecule (as an example) in **11g**, Å

O1-C12	1.3482(14)	N2-C2	1.3339(15)	C6-C7	1.4012(17)
O1-C13	1.4383(14)	N3-C1	1.3553(15)	C7-C8	1.3888(16)
O2-C6	1.3632(14)	N3-C16	1.4516(16)	C8-C9	1.5339(16)
O2-C18	1.4302(15)	N3-C17	1.4543(15)	C9-C10	1.5505(16)
O3-H3O	0.89(2)	C2-C3	1.4738(16)	C9-C13	1.5324(16)
O3-C9	1.4277(14)	C2-C14	1.4010(16)	C10-C11	1.4473(18)

O4-C10	1.2244(16)	C3-C4	1.3929(16)	C11-C12	1.3461(19)
N1-C1	1.3598(16)	C3-C8	1.4189(16)	C12-C19	1.4890(17)
N1-C15	1.3443(15)	C4-C5	1.3904(16)	C13-C14	1.4974(15)
N2-C1	1.3505(15)	C5-C6	1.3950(16)	C14-C15	1.3754(17)

Table S4. Hydrogen bonds for **11g**·½EtOH, Å and °.

D-H...A	d(D-H)	d(H...A)	d(D...A)	<(DHA)
O3-H3O...O50	0.89(2)	1.77(2)	2.6459(13)	168.8(18)
O7-H7O...N28#1	0.898(19)	2.02(2)	2.9207(13)	176.9(17)
O11-H11O...N13#2	0.85(2)	1.97(2)	2.8193(13)	174.9(18)
O15-H15O...N16#3	0.88(2)	1.90(2)	2.7779(13)	177.0(17)
O19-H19O...N10	0.86(2)	1.91(2)	2.7651(13)	174.3(18)
O23-H23O...N1#1	0.93(2)	1.93(2)	2.8563(13)	173.9(17)
O27-H27O...N4#2	0.88(2)	1.97(2)	2.8440(13)	177.2(17)
O31-H31O...O54	0.89(2)	1.80(2)	2.6559(13)	161(2)
O35-H35O...N19	0.901(19)	1.872(19)	2.7708(13)	174.6(17)
O39-H39O...N34	0.93(2)	2.04(2)	2.9667(14)	176.1(18)
O43-H43O...N25	0.90(2)	1.90(2)	2.7959(13)	175.7(17)
O47-H47O...N31#4	0.97(2)	1.92(2)	2.8805(13)	172.6(18)
O49-H49O...O19#5	0.85(3)	2.09(3)	2.8925(13)	157(2)
O50-H50O...N22#6	0.91(2)	1.80(2)	2.6911(14)	163(2)
O51-H51O...O23#7	0.96(2)	1.88(2)	2.8397(13)	174(2)
O52-H52O...O13#8	0.85(3)	2.65(3)	3.3030(13)	135(2)
O52-H52O...O15#8	0.85(3)	2.09(3)	2.8727(13)	153(2)
O53-H53O...O11	0.88(2)	2.02(2)	2.8455(13)	155(2)
O54-H54O...N7	0.95(2)	1.86(2)	2.7513(14)	154(2)

Symmetry transformations to generate equivalent atoms: #1 x-1, y, z; #2 -x+1, -y+1, -z+1; #3 x, y-1, z; #4 -x+1, -y, -z; #5 -x+1, -y, -z+1; #6 -x+1, -y+1, -z; #7 x+1, y, z; #8 x, y+1, z.

7. References

1. Komogortsev A.N.; Lichitsky B.V.; Tretyakov A.D.; Fakhrutdinov A.N.; Dudinov A.A.; Krayushkin M.M. *J Heterocyclic Chem.* **2019**, 56, 3081–3087.

2. CrysAlisPro. Version 1.171.41. *Rigaku Oxford Diffraction*, **2021**.

3. Sheldrick G. M. *Acta Cryst.* **2015**, A71, 3-8.

4. Sheldrick G. M. *Acta Cryst.* **2015**, C71, 3-8.

5. Dolomanov O.V.; Bourhis L.J.; Gildea R.J.; Howard J.A.K.; Puschmann H. *J. Appl. Cryst.* **2009**, 42, 339-341.
